# SARS-CoV-2 RNAs are processed into 22-nt vsRNAs in Vero cells

**DOI:** 10.3389/fimmu.2022.1008084

**Published:** 2022-10-28

**Authors:** Yang Liu, Jian Rao, Yingjie Mi, Lan Chen, Lijuan Feng, Qi Li, Jianing Geng, Xianguang Yang, Xiangjiang Zhan, Lili Ren, Jinfeng Chen, Xiaoming Zhang

**Affiliations:** ^1^ State Key Laboratory of Integrated Management of Pest Insects and Rodents, Institute of Zoology, Chinese Academy of Sciences, Beijing, China; ^2^ Department of Life Sciences, Henan Normal University, Xinxiang, Henan, China; ^3^ CAS Center for Excellence in Biotic Interactions, University of Chinese Academy of Sciences, Beijing, China; ^4^ National Health Commission (NHC) Key Laboratory of Systems Biology of Pathogens and Christophe Mérieux Laboratory, Institute of Pathogen Biology, Chinese Academy of Medical Sciences & Peking Union Medical College, Beijing, China; ^5^ College of Life Sciences, Institute of Life Sciences and Green Development, Hebei University, Baoding, China; ^6^ State Key Laboratory of Microbial Resources, Institute of Microbiology, Chinese Academy of Sciences, Beijing, China; ^7^ Key Laboratory of Animal Ecology and Conservation Biology, Institute of Zoology, Chinese Academy of Sciences, Beijing, China; ^8^ Center for Excellence in Animal Evolution and Genetics, Chinese Academy of Sciences, Kunming, China; ^9^ Key Laboratory of Respiratory Disease Pathogenomics, Chinese Academy of Medical Sciences and Peking Union Medical College, Beijing, China

**Keywords:** SARS-CoV-2, microRNAs, virus-derived small RNAs, immunity, IFN

## Abstract

The severe acute respiratory syndrome coronavirus 2 (SARS-CoV-2) has caused the global pandemic, resulting in great fatalities around the world. Although the antiviral roles of RNA interference (RNAi) have been well studied in plants, nematodes and insects, the antiviral roles of RNAi in mammalians are still debating as RNAi effect is suspected to be suppressed by interferon (IFN) signaling pathways in most cell types. To determine the role of RNAi in mammalian resistance to SARS-CoV-2, we studied the profiling of host small RNAs and SARS-CoV-2 virus-derived small RNAs (vsRNAs) in the early infection stages of Vero cells, an IFN-deficient cell line. We found that host microRNAs (miRNAs) were dysregulated upon SARS-CoV-2 infection, resulting in downregulation of microRNAs playing antiviral functions and upregulation of microRNAs facilitating viral proliferations. Moreover, vsRNA peaked at 22 nt at negative strand but not the positive strand of SARS-CoV-2 and formed successive Dicer-spliced pattern at both strands. Similar characteristics of vsRNAs were observed in IFN-deficient cell lines infected with Sindbis and Zika viruses. Together, these findings indicate that host cell may deploy RNAi pathway to combat SARS-CoV-2 infection in IFN-deficient cells, informing the alternative antiviral strategies to be developed for patients or tissues with IFN deficiency.

## Introduction

The global pandemic of severe acute respiratory syndrome coronavirus 2 (SARS-CoV-2) has caused over 572,239,451 infections and 6,390,401 fatalities around the world since late 2019 (data collected from WHO website on July 25^th^, 2022). As an enveloped, positive sense, single-stranded RNA betacoronavirus, SARS-CoV-2 is a member of *Coronavirvidae* family, which consists of two other members, severe acute respiratory syndrome (SARS-CoV) emerged in 2002 and Middle East respiratory syndrome (MERS) in 2012 (1, 2). All these three coronaviruses are highly pathogenic and can cause severe respiratory diseases in human. However, different with SARS-CoV and MERS, which lead to 10% mortality for SARS-CoV and 37% for MERS, SARS-CoV-2 spreads more efficiently with a relatively lower level of mortality of 2%~3% (3, 4). SARS-CoV-2 shares the same receptor angiotensin-converting enzyme 2 (ACE2) with SARS-CoV, using spike (S) protein for ACE2 binding as well as host serine protease TMPRSS2 for S protein primming to achieve viral and cellular membrane fusion and finally cell entry (5). Symptoms accompanied as viral invasion are not limited to respiratory failure, but in multiple organ systems, including brain (6, 7), liver (8) and gastrointestinal tract (9). The cytokine storm stimulated by viral infection is one of the main death causes of SARS-CoV-2 (10). Besides, SARS-CoV-2 infection might induce the new-onset diabetes, suggesting the potential health risk exists upon viral infection (11).

Mammalian cells have evolved multiple antivirus mechanisms, among which the interferon (IFN) signaling-mediated innate immune responses are characterized as critical and effective pathway for antiviral defense. In these pathways, viral RNA is recognized by several receptors such as retinoic acid-inducible gene I (RIG-I) (12) and melanoma differentiation-associated gene 5 (MDA5) (13) and subsequently triggers the producing and secretion of type I IFNs (mainly IFN-α and IFN-β) (14), followed by activation of various interferon-stimulated genes (ISGs) against viral invasion (15, 16). RNA interference (RNAi) is another crucial antiviral mechanism in eukaryotes. It regulates gene expression through 20-30 nucleotide (nt) small RNAs (sRNAs) processed from double-stranded or base-paired RNA (17, 18). sRNAs can be classified into microRNAs (miRNAs), small interference RNAs (siRNAs) and piwi-interacting RNAs (piRNAs) based on their differences in biogenesis pathways (19). The accumulation of partial host miRNAs is significantly altered to modulate the interactions between hosts and viruses. Some viruses also encode miRNAs to facilitate their infections or proliferations (20, 21). RNAi plays major roles in plant resistance to viruses (22). Viral RNAs and DNAs in plants and invertebrates are targeted by vsRNAs to hinder the proliferations of viruses (18). However, the antiviral capacity of vsRNAs in mammalian remains much to debate (23, 24). Studies have demonstrated the antiviral ability of RNAi in undifferentiated cells such as oocytes, embryonic teratocarcinoma cell lines and mouse embryonic stem cells (mESCs) but not in highly differentiated somatic cells (25–27). This might be caused by the existence of IFN pathway that represses RNAi by inhibiting siRNA processing (28, 29). Prior studies suggest that suppression of IFN pathway is able to activate RNAi antivirus function in differentiated mouse cells (28, 30).

Recent studies found that SARS-CoV-2 genome encodes miRNAs and inhibit host immune response by targeting the IFN signaling pathway as well as metabolic pathways (31–33), which have been reported to be impaired in SARS-CoV-2 patients (34). However, most of the studies are performed in cell lines expressing interferon. The accumulation of SARS-CoV-2 vsRNAs and their possible regulatory roles in host-SARS-CoV-2 interaction when IFN crippled still remains unclear. To understand the function RNAi exerts in response to SARS-CoV-2 infection, we set out to construct the sRNA libraries and characterize the host miRNAs and viral sRNAs during the early stages of viral infection in Vero cells, which are uncapable of producing IFN-αand IFN-β (35). We found that the accumulation of host miRNAs related to pathological processes or antiviral defense is modulated upon the infection of SARS-CoV-2, indicating that host miRNAs may play important roles in host-SARS-CoV-2 interaction. We observed characteristics of Dicer processed vsRNAs at negative strand of SARS-CoV-2, including a predominant 22-nt peak, preference of uracil at 5’ terminal, and successive 22-nt reads. We then characterized the accumulation of viral sRNAs in other cell lines infected with distinct viruses. We found that these characteristics at negative strand of viral genome were also identified in IFN-deficient Vero cells infected with Sindbis virus (SINV) (36) as well as IFN-deficient neural stem cells infected with Zika virus (ZIKV) (37–39). Together, these results suggest that SARS-CoV-2 transcripts in Vero cells were likely to be processed into vsRNAs by Dicers and may play similar important antiviral roles as in plants and invertebrates. These observations will lead to development of alternative antiviral strategies against SARS-CoV-2 in patients with IFN-deficiency or tissues that lack IFN producing.

## Results

### Host sRNAs in Vero cells react to SARS-CoV-2

**Figure 1:**
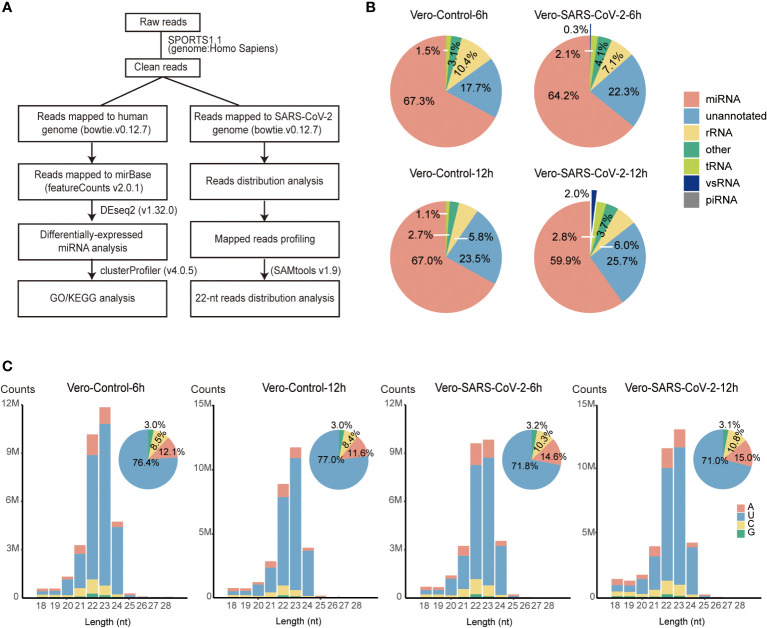
Overview of small RNAs in SARS-CoV-2-infected Vero cells. **(A)** The schematic workflow for small RNA analysis. **(B)** Fractions of small RNAs in control and virus-infected (6 hpi/12 hpi) Vero cells. **(C)** Size distribution of total sRNAs in control and virus-infected (6 hpi/12 hpi) Vero cells. Clean reads are aligned to the human reference. Mapped reads without matching to tRNA and rRNA are classified by length and are shown as read counts. Pie plots represent sRNAs initiated with A/U/C/G in control and virus-infected Vero cells.

To get a comprehensive profiling of sRNAs in IFN-deficient cell lines, we sequenced and analyzed sRNAs in Vero cells infected with SARS-CoV-2 at a multiplicity of infection of 0.1 (MOI = 0.1). Control and SARS-CoV-2 infected Vero cells were harvested at 6 and 12 hours post infection (hpi). sRNA libraries of two biological replicates were constructed and sequenced from both control and SARS-CoV-2 infected Vero cells. After trimming adapters and filtering low-quality reads, ~20-32 million clean reads were obtained for each library (**Table S1**). We evaluated the reproducibility of libraries using 2,652 known miRNAs obtained from miRbase (http://www.mirbase.org). The correlation analysis showed that miRNA transcription of two biological replicates were highly correlated (*R* ≥ 0.99, *P* < 2.2e-16, **Figure S1**). We then processed clean reads with small noncoding RNA analysis pipeline from SPORTS1.1, using the human (hg38) and viral (NC_045512.2) genomes as references (**Figure 1A** and **Table S1**). In total, 59.8%-69.8% of sRNA reads were annotated as miRNA in each sample. The rest sRNAs were annotated as rRNA (2.3%-12.8%), tRNA (1.0-2.9%), unannotated RNA (17.7%-26.0%) and vsRNA (0-2.3%) (**Table S1**). SARS-CoV-2 infected Vero cells showed decreased abundance of miRNA (67.3% vs. 64.2% at 6 hpi and 67.0% vs. 59.9% at 12 hpi) while increased accumulation of tRNA (1.5% vs. 2.1% at 6 hpi and 1.1% vs. 2.8% at 12 hpi), unannotated RNA (17.7% vs. 22.3% at 6 hpi and 23.5% vs. 25.7% at 12 hpi) and vsRNA (0% vs. 0.3% at 6 hpi and 0% vs. 2.0% at 12 hpi) compared to control cells (**Figure 1B** and **Table S1**). These results indicate that SARS-CoV-2 infection altered sRNA populations of Vero cells.

sRNAs length distribution peaked at 23 nt with 58.8%-63.0% sRNAs 22-23 nt in length, which is different from 22-nt main peaks that were observed in other cell lines infected with SARS-CoV-2 (**Figure 1C** and **Table S1**) (31, 40). Further investigation identified that the prompt accumulation of miR-21, combining with 23-nt miR-21 isoforms, accounting for 18.8%-27.2% of clean reads mapped to human genome (**Table S2**) (41). After removing these two miR-21 isomiRs, sRNA length distribution peaked back at 22 nt (**Figure S2A**). Most of sRNAs (71.0%-77.0%) initiated with “U” (**Figure 1C**). Together, these results suggest that the majority of host sRNAs were generated by Dicers through the canonical miRNA processing pathway.

### Host miRNAs react to SARS-CoV-2 infections in Vero cells

**Figure 2:**
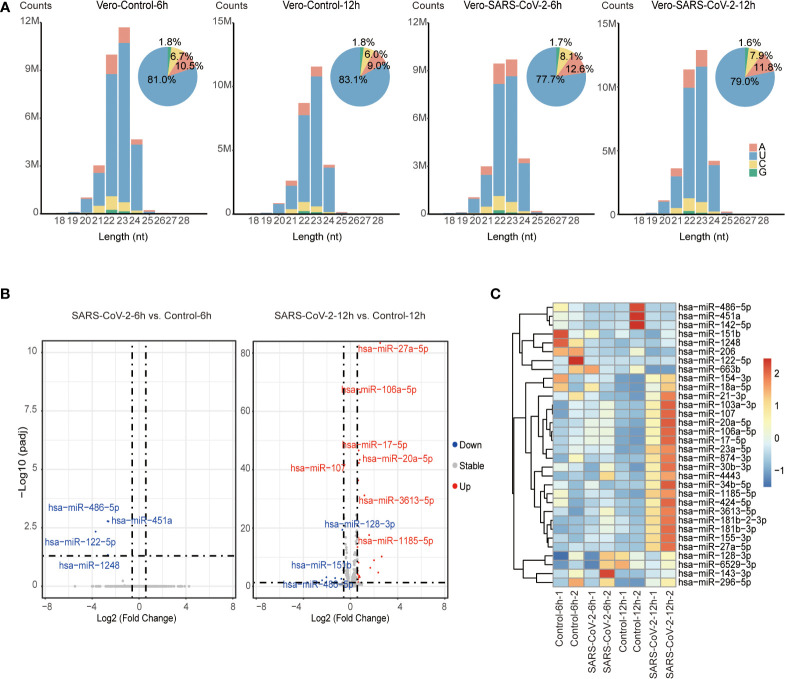
Identification of DE miRNAs in SARS-CoV-2 infected Vero cells. **(A)** Size distribution of miRNA reads in control and virus-infected (6 hpi/12 hpi) Vero cells. miRNA reads are classified by length and are shown as read counts. Pie plots represent miRNAs initiated with A/U/C/G in control and virus-infected Vero cells. **(B)** Volcano plot showing the differentially expressed miRNAs in Vero cells infected with SARS-CoV-2 6 hpi/12 hpi vs. controls (n=2). Red dots and blue dots respectively represent up-regulated and down-regulated miRNAs (fold change ≥ 1.5, adjusted *P* value ≤ 0.05). **(C)** Heatmap of human miRNA expression in control and virus-infected (6 hpi/12 hpi) Vero cells. Only miRNAs that are significantly changed are shown in heatmap.

To identify host miRNAs that response to SARS-CoV-2 infection, we explored miRNA expression pattern in virus-infected cells compared to their non-infected controls. In total, 27.1 to 33.7 million miRNA reads were obtained from combined control and SARS-CoV-2 libraries (**Table S1**). Similar to the total sRNA distribution, miRNA reads were enriched in 22-23 nt and peaked at 23-nt due to the high abundance of miR-21 as well as its 23-nt isoform miR-21+C (**Figure 2A** and **Table S2**). miRNA distribution peaked at 22 nt after trimming off all these 22 and 23-nt miR-21 isoforms (**Figure S2B**).

We then analyzed differentially expressed (DE) miRNAs between SARS-CoV-2-infected Vero cells and control cells. A total of 32 DE miRNAs including 22 up-regulated and 10 down-regulated miRNAs were identified by comparing the infected and uninfected sRNA libraries (**Figure 2B** and **Table S3A**). Among them, 4 down-regulated miRNAs were identified at 6 hpi, while 22 up-regulated and 7 down-regulated miRNAs were identified at 12 hpi. Only 1 miRNA, miR-486-5p, was dysregulated at both 6 hpi and 12 hpi. However, we observed that miRNAs that were altered at 12 hpi showed similar tendency but subtle changes at 6 hpi, suggesting miRNA responded to SARS-CoV-2 infection as early as 6 hpi (**Figure 2C**). Moreover, several dysregulated miRNA including 4 down-regulated miRNAs (miR-486-5p, miR-451a, miR-206, and miR-142-5p) and 5 up-regulated miRNAs (miR-155-3p, miR-27a-5p, miR-20a-5p, miR-17-5p, and miR-107) were associated with pathophysiology in response to viral replication (42–49). Among them, miR-486-5p, miR-451a, miR-142-5p, miR-27a-5p, miR-20a-5p, miR-17-5p have been reported to be dysregulated in SARS-CoV-2-infected patients (50–53), while others such as miR-206, miR-155-3p and miR-107 have not been associated with SARS-CoV-2 viral infection, suggesting that host miRNAs reacting at early stage of SARS-CoV-2 infection might play important roles in host-SARS-CoV-2 interaction.

### Viral infection-response pathways targeted by host DE miRNAs

**Figure 3:**
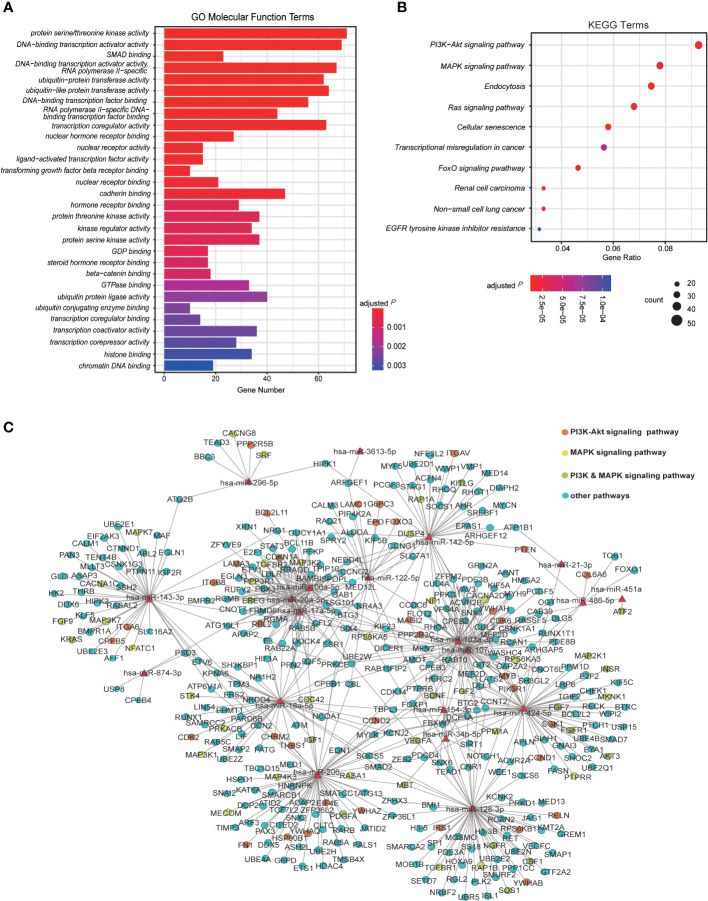
Analysis of DE miRNAs and their regulatory pathway. **(A)** The Gene Ontology (GO) analysis for the enriched functions of genes regulated by the host DE miRNAs induced by viral infection. The top 30 molecular function-related GO enrichment terms of target genes are shown. Terms with adjusted *P* ≤ 0.05 are recognized as significantly enriched GO terms. **(B)** The Kyoto Encyclopedia of Genes and Genomes (KEGG) analysis of target genes significantly regulated by DE miRNAs in Vero cells infected with SARS-CoV-2 6 hpi/12 hpi vs. controls (n=2). **(C)** The regulatory network of both up-regulated and down-regulated human miRNA and their target genes enriched in KEGG pathways. The red triangle nodes represent the miRNAs. The red dots are genes specifically regulating PI3K-Akt signaling pathway, yellows are genes specifically regulating MAPK signaling pathway, greens are genes in both pathways, and genes in other pathways are presented in blue.

To determine the potential roles of dysregulated host miRNAs during SARS-CoV-2 infection, we performed target gene prediction for dysregulated miRNAs and explored their functional implication in host-SARS-CoV-2 interaction. A total of 1,364 putative target genes were predicted with mirDIP (**Table S3B**). Of them 893 were modulated by up-regulated miRNAs, 629 were modulated by down-regulated miRNAs, and 158 target genes were modulated by both up-regulated and down-regulated miRNAs. GO and KEGG pathway enrichment analysis revealed 110 GO terms and 62 biological pathways impacted by dysregulated miRNAs during SARS-CoV-2 infection (**Table S3C** and **Table S3D**). In molecular function analysis of GO terms, an array of target genes was enriched in terms including “protein serine/threonine kinase activity”, “DNA-binding transcription activator activity”, “DNA-binding transcription factor activator activity, RNA polymerase II specific”, “SMAD binding”, “ubiquitin-protein (like) transferase activity” and “transcription coregulator activity”, indicating the transcription, degradation and catalytic activities altered by miRNA dysregulation (**Figure 3A** and **Table S3C**). To get a better reflection of the pathways that DE miRNA targets were involved in, we then performed KEGG analysis (**Figure 3B** and **Table S3D**). A variety of irritated genes were significantly enriched in phosphoinositol 3-kinase/serine-threonine kinase (PI3K-Akt) and mitogen-activated protein kinase (MAPK) signaling pathways, which are closely associated with cell cycle, stress response and cell proliferation (54–56). Besides, targeting genes are also enriched in Ras signaling pathway that is activated during hepatitis C virus (HCV) infection and promotes viral replication of HCV (**Figure 3B**) (57). Moreover, biological processes as endocytosis, senescence and tumorigenesis are also enriched in KEGG analysis.

To get a comprehensive view of the relationship between host miRNAs and their potential target genes, the interaction network of DE miRNAs and their targets enriched in KEGG pathways were then built up (**Figure 3C**). Intriguingly, partial of the up-regulated miRNAs shared numbers of common targets, of which genes in PI3K-Akt and MAPK pathways promised a large proportion (**Figure 3C** and **Table S3B**). In contrast, the down-regulated miRNAs appeared to share few targets with each other (**Figure 3C**). Taken together, these results suggest that host miRNAs in Vero cells may contribute the host-SARS-CoV-2 interaction by modulating viral infection responses and transcriptional reprogramming of host.

### 22-nt SARS-CoV-2 vsRNAs accumulate in Vero cells

**Figure 4:**
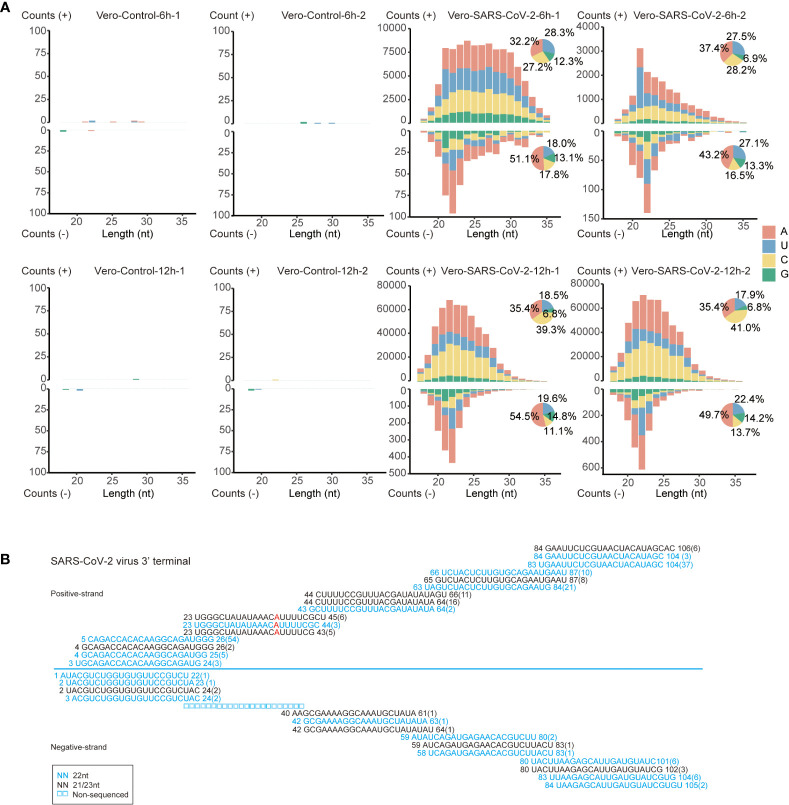
The inducement of RNAi in SARS-CoV-2 infected Vero cells. **(A)** Size distribution and abundance of SARS-CoV-2 vsRNAs sense and antisense reads from Vero cells at 6 and 12 hpi. Pie plots represent vsRNAs initiated with A/U/C/G in positive and negative strands. **(B)** Read sequences along the 3’-terminal sequence of SARS-CoV-2 viral genome. Read counts (in brackets), read length, non-sequenced reads and genomic position are indicated and mismatched bases are mentioned in red.

To determine the potential antiviral roles of RNAi in IFN-deficient Vero cells, we analyzed the accumulation of vsRNAs in the mock and SARS-CoV-2-infected sRNA libraries. Firstly, sRNAs that were specifically matched to positive or negative strand of viral genome were extracted. Both positive and negative-stranded sRNAs were distributed along viral genome (**Figure S3A**), which suited the key features of vsRNAs (27). These vsRNAs were particularly enriched at 3’ terminus of viral genome (**Figure S3A**). We then sorted those vsRNAs by their length and the size-distribution analysis were carried out on the basis of sRNA length. No dominant accumulating peaks were obtained for vsRNAs matched to the positive strand of SARS-CoV-2 genome, except for a 21-nt peak in one of the 6 hpi libraries after viral infection (**Figure 4A**). In contrast, a 22-nt accumulating peak was observed at negative strand of viral genome at both 6 hpi and 12 hpi (**Figure 4A**). The majority of negative-stranded 22-nt vsRNAs were more likely to initiate with “A” and “U”, together occupying a proportion of 65.7%-77.1%, which indicates the possibility that the 22-nt vsRNAs might be sorted into the Argonaute protein to take functions (**Figure 4A** and **Table S4**) (58).

Characteristics of 22-nt vsRNAs at negative strand of viral genome suggest that SARS-CoV-2 transcripts might be processed by host RNAi machineries in the early infection stages of Vero cells. We analyzed the size distribution of vsRNAs to determine their dicing pattern. Namely, the 21 to 23-nt vsRNAs are derived from both strands of viral genome. We also found that the 22-nt vsRNAs were highly enriched at the 3’ end of the SARS-CoV-2 genome (**Figure S3B**), forming the 20-nt perfect base-paired duplex with 2-nt overhang at 3’ terminal (**Figure 4B**). Besides, these 21 to 23-nt duplexes formed successive complementary pairs along the viral genome, which underlies the processing of these vsRNAs from viral double-stranded RNA replicative intermediate by Dicer (59). These results indicate that the SARS-CoV-2 genomic RNAs in Vero cells may be processed by host Dicer enzyme into 22-nt vsRNAs.

### 22-nt vsRNAs are enriched in IFN-deficient cell lines

The processing of SARS-CoV-2 genomic RNAs in Vero cells by Dicer enzyme into 22-nt vsRNAs may be either a specialty of SARS-CoV-2 or a specialty of the Vero cell. To test whether the accumulation of 22-nt vsRNAs is a common effect caused by SARS-CoV-2 infection, we performed vsRNA analysis using published small RNA data from various kinds of SARS-CoV-2 infected cell lines (**Figure S4** and **Table 1**) (31–33, 60, 61). The accumulations of SARS-CoV-2 vsRNAs in other cell lines, including Calu-3, A549-hACE2 and PC-9 were analyzed. No obvious dominant accumulation of 22-nt vsRNAs was observed in all cell examined, except that A549-hACE2 24 hpi, Calu-3 24 hpi (**Figure S4A**, **S4C**) cell lines exhibit abundant 22-nt vsRNA accumulation at negative strand. However, no signatures of 20-nt continuous complementary pairs or 2-nt 3’ overhang were observed in these libraries. These results argue against processing of SARS-CoV-2 genomic RNA into 21 to 23-nt vsRNAs, by Dicer in these cell lines.

**Table 1:** The appears of 22-nt negative-strand peak of vsRNAs in different IFN-deficient or normally-expressed cell lines infected with different viruses.

Cell type	Sample name	Host cell line	Virus	Virus genome	Peak characterization	Reference
**IFN normally-expressed cell line**	Calu-3-SARS-CoV-2-6h/24h/48h	Calu-3	SARS-CoV-2	(+)ssRNA	22-nt peak in negative strand at 24 hpi	(33)
	PC-9-SARS-CoV-2-6h/24h/36h	PC-9	SARS-CoV-2	(+)ssRNA	No prominent 22-nt peak	
	A549-hACE2-SARS-CoV-2-6h/24h/48h	A549-hACE2	SARS-CoV-2	(+)ssRNA	22-nt peak in negative strand at 24 hpi	
	A549-hACE2- SARS-CoV-2-24h/48h	A549-hACE2	SARS-CoV-2	(+)ssRNA	No prominent 22-nt peak	(32)
	Calu-3-SARS-CoV-2	Calu-3	SARS-CoV-2	(+)ssRNA	No prominent 22-nt peak	(31)
	Calu-3-SARS-CoV-2-4h/12h/24h	Calu-3	SARS-CoV-2	(+)ssRNA	22-nt peak in negative strand at 24 hpi	(60)
	Calu3_MOI10-SARS-CoV-2-24h/36h & Calu3_MOI01-SARS-CoV-2-48h	Calu-3	SARS-CoV-2	(+)ssRNA	No prominent 22-nt peak	(61)
	---	HEK293	SINV	(+)ssRNA	22-nt peak in negative strand at 24 hpi	(36)
**IFN-deficient cell line**	Vero-Sindbis	Vero	SINV	(+)ssRNA	Prominent 22-nt peak in negative strand	(36)
	---	Vero	ZIKV	(+)ssRNA	Prominent 22-nt peak in negative strand	(62)
	NSCs-Zika-1	Neural Stem Cell (NSC)	ZIKV	(+)ssRNA	Prominent 22-nt peak in negative strand	(37)
	NSCs-Zika-2	Neural Stem Cell (NSC)	ZIKV	(+)ssRNA	Prominent 22-nt peak in negative strand	(38)
	NSCs-Zika-3	Neural Stem Cell (NSC)	ZIKV	(+)ssRNA	Prominent 22-nt peak in negative strand	(39)
	NPCs-Zika	Neural Progenitor Cells (NPCs)	ZIKV	(+)ssRNA	Prominent 22-nt peak in negative strand	(63)

Therefore, the accumulation of 22-nt vsRNAs may be a specialty for Vero cell. We thus set to analyze the size distribution of vsRNAs in Vero cell lines infected with other viruses in previous reports. Similarly, the most abundant 22-nt vsRNA accumulation at negative strands of viral genome also exist in SINV and ZIKV-infected Vero cells **Table 1**, **S5**) (36, 62). Moreover, SINV exhibit similar continuous complementary pairs with 20-nt perfect base-pair and 2-nt 3’ overhang (**Figure S5**), whereas no defined peaks at specific length for vsRNAs matched to the positive strands of SINV were observed (36). Combined with the former findings on specific stimulation of 22-nt vsRNAs at both strands upon ZIKV infection in human neural stem cells (NSCs) and human neural progenitor cells (NPCs) (**Table 1**, **S5**) (37, 39, 63), in which the IFN signaling pathway is restricted, the accumulation of 22-nt vsRNAs in IFN-deficient cells is more likely to be induced.

## Discussion

This study aimed to reveal the coherently altered host miRNA and vsRNA profiles in innate immunity-deficient cell line upon early infection of SARS-CoV-2. We found that portion of host miRNA population varied along with SARS-CoV-2 proliferation. The targets of these miRNAs are enriched in transcription and catalytic activity, and viral infection responses. A predominant peak of 22-nt vsRNAs that match to the negative strand of SARS-CoV-2 genome was observed in Vero cells. We then observed similar distribution patterns of vsRNAs for several other viruses in other IFN-deficient cell lines.

Although no obvious increasement in miRNA proportion was observed after viral infection, the accumulations of some miRNAs were altered upon the infections of SARS-CoV-2. Among the down-regulated miRNAs, the only miRNA that was down-regulated at both 6 and 12 hpi is miR-486-5p. This miRNA has been reported to act as an oncogene in various cancers, promote proliferation, invasion and metastasis of multiple cancers (64, 65). Furthermore, a previous study has revealed the function miR-486-5p played in defense against influenza A virus (42). miR-451a was recently reported to inhibit hepatitis B virus replication through directly targeting activating transcription factor 2 gene (43), this miRNA was also down-regulated in our SARS-CoV-2 libraries. In agreement with our findings, miR-486-5p and miR-451a were also found significantly decreased in plasma samples of critical patients infected by SARS-CoV-2 (50). The down-regulation of miR-451a in cell-free circulating RNA of SARS-CoV-2 infected patients was also determined, which caused elevated accumulation of its downstream target, IL-6R, and probable cytokine storms (66). Except for miR-486-5p and miR-451a, other down-regulated miRNAs are also proven to take functions in viral infection. miR-206, for instance, elicits its anti-influenza A virus activity by targeting tankyrase 2 to restrict viral replication (44), and miR-142-5p, whose accumulation is significantly inhibited by ZIKV, acts as an antiviral factor during ZIKV infection (45). All these evidences have implied the strategy that viruses may adopt for better survival by down-regulating specific miRNAs, in order to repress the function of them in antivirus immunity.

On the other hand, SARS-CoV-2 may increase the expression of specific part of miRNAs to repress the antiviral immune response of host cells, benefiting viral infection and replication. Of the up-regulated miRNAs, one common miRNA, miR-155-3p, has been reported to participate in various pathological processes, including tumor formation, proliferation, progression as well as influenza A viral infection (46, 67, 68), and was also found significantly up-regulated in both SARS-CoV and SARS-CoV-2 infected Calu-3 cells in a previous study (60), implying a small partial of conserved miRNAs triggered by coronavirus infection. In addition, miR-27a-5p, miR-20a-5p, miR-17-5p and miR-107 are of the most abundant and intensively studied miRNAs. These miRNAs, however, tend to play inhibitory roles in antiviral immunity. miR-27a-5p accumulation is reported to be elevated in Porcine reproductive and respiratory syndrome virus-infected MARC-145 cells and enhances viral replication to a large extent (47). In addition, miR-20a-5p and miR17-5p are found to be up-regulated in influenza A virus-infected patients and extracellular vesicles of lung epithelial tissue, and miR17-5p is proven to promote influenza A virus replication (48, 69). Similarly, miR-107 is demonstrated to regulate negatively in response to both DNA and RNA viral infection (49). Therefore, the specifical alteration of miRNAs implicates that SARS-CoV-2 may manage to repress host antivirus immunity for its pathogenicity.

We then found that PI3K-Akt, MAPK and Ras signaling pathways are enriched in our KEGG analysis of miRNA target analysis (**Figure 3B**). PI3K-Akt and MAPK signaling pathway are key signaling pathways that participate in multiple fundamental cellular biological processes and has been demonstrated to enhance viral replication in multiple viruses, including MERS (70), enterovirus 71 (71) and ZIKV (72). Considering that the inhibition of PI3K/AKT/mTOR and MAPK/ERK pathway by kinase inhibitors suppresses MERS replication *in vitro*, and the fact that MERS and SARS-CoV-2 share great similarity in viral genome (73), the enrichment of DE miRNA targets in these two pathways also provides a new strategy as pharmacological targets in SARS-CoV-2 treatment (74). Activated during HCV infection, Ras signaling pathway promotes HCV replication (57). Our GO analysis showed that transcription and catalytic activity might be altered by miRNA dysregulation (**Figure 3A**). Since viral infection may cause the reprogramming of cellular physiological and biochemical processes, it makes sense that some of the target genes were also related to biological processes as endocytosis, senescence and tumorigenesis (**Figure 3B**). Intriguingly, no IFN signaling pathway genes showed any enrichment in both GO and KEGG analysis, nor did the downstream ISGs trigged by interferons, for the absence of IFN system of Vero cells would not give rise to stimulation of various ISGs.

Recent studies have proved the existence of virus-encoded miRNAs and their function in immune response. A miRNA-like sRNA encoded by SARS-CoV-2 ORF7a transcript, CoV2-miR-O7a, was discovered to target host ISG-Basic 11 Leucine Zipper ATF-Like Transcription Factor 2, subsequently attenuating its accumulation to suppress the interferon signaling pathway (33). Another study additionally verified the two isoforms of CoV2-miR-O7a.1 and CoV2-miR-O7a.2 to be produced by Dicer and then loaded into Argonaute proteins, and could act as host miRNAs and target the 3’UTR of ISGs, in order to restrain interferon-mediated innate immune response (32). Interestingly, the predominant isoform CoV2-miR-O7a.2 that mainly took functions was found in our libraries, suggesting that SARS-CoV-2-encoded miRNAs may exist and in part take effects in Vero cells.

Despite of the better understanding of host miRNA profile variation upon SARS-CoV-2 infection and SARS-CoV-2 encoded miRNAs, little is known about whether SARS-CoV-2 triggers vsRNA producing in mammalian cells. Past studies have also indicated the suppression of antiviral RNAi by IFN system (28), and the inhibition of IFN signaling pathway unveiled the presence of antiviral RNAi (30). Antiviral RNAi was detected in both mESCs infected with picornavirus encephalomyocarditis virus and suckling mice infected with VSR ablated Nodamura virus (27, 75), showing the underlying antiviral effect RNAi might take when IFN pathway was repressed. Our findings reveal the producing of SARS-CoV-2-derived 22-nt vsRNAs matched to the negative but not the positive strand of SARS-CoV-2 genome in Vero cells. Moreover, these SARS-CoV-2 vsRNAs formed successive complementary pairs along viral genome, which suggests the existence of antiviral RNAi that was thought to be abandoned by mammalian cells. Moreover, similar distribution patterns were obtained for SINV in Vero cell lines. The 22-nt in-length vsRNAs implicates the canonical cleavage by Dicer protein, however, the activation of RNAi needs to be experimentally confirmed.

Previous studies have demonstrated the presence of antiviral RNAi when IFN signaling pathway is inhibited (30). Therefore, the accumulation of SARS-CoV-2 vsRNAs in interferon-deficient Vero cells indicates that the malfunctions of IFN signaling pathways may lead the function of antiviral RNAi and the generation of 22-nt vsRNAs. To test this hypothesis, we subsequently summarized the accumulation mode of vsRNAs in other interferon-deficient cell lines that infected with distinct viruses in published sRNA libraries (**Table 1**) (36–39, 62, 63). The vsRNA profiling of ZIKV in IFN-deficient cell lines, including Vero cells, neural stem cells and human neural progenitor cells, displays similar accumulation mode of 22-nt vsRNAs at negative strand of ZIKV genome (**Table 1**) (37–39, 62, 63). Similarly, no dominant accumulating peaks were obtained for Zika vsRNAs matched to the positive strands of viral genome, except for ZIKV-infected human neural progenitor cells. Moreover, ZIKV also exhibits similar continuous complementary pairs with 20-nt perfect base-pair and 2-nt 3’ overhang both in cell culture as well as central neuron system where immune responses to viral infection are highly regulated (37, 62, 63). In a word, these evidences suggest that the 22-nt vsRNAs tend to be stimulated in IFN-deficient cells upon infection of multiple RNA viruses.

Intriguingly, vsRNAs with 22 nt in length appeared to be of the most abundant among those distributed along the antisense of SARS-CoV-2 viral genome in Calu-3 and A549-hACE2 cells, the IFN-competent cell lines, after 24 hours viral infection (**Figure S4** and **Table 1**) (33, 60). This might be explained by former studies, which have demonstrated the inhibitory effects of SARS-CoV-2 on type I IFN production as well as downstream signaling pathway *via* its structural and nonstructural proteins, such as NSP13, NSP14 and ORF6 (76, 77). The antagonism of SARS-CoV-2 on type I IFN responses could in part cancel the inhibition of interferon on antiviral RNAi, making the generation of antiviral RNAi of mammalian cells possible to be observed. Moreover, positive strand RNA viruses like SINV and ZIKV also induce the generation of 22-nt vsRNAs in other IFN-competent cells and tissues. For example, vsRNA peaked at 22-nt at negative strand of SINV genome in another IFN-competent cell line-HEK293 cell line (36, 62), and 22-nt vsRNAs significantly accumulate at both strands of ZIKV genome in ZIKV-infected hindlimb muscle tissue of suckling mice (62). Both viruses were also reported to elicit antagonizing effect on IFN response through nonstructural proteins (78, 79), nsP2 for SINV and NS1 for ZIKV, that might account for the stimulation of suspected antiviral RNAi in IFN-competent cells and tissues.

Taken together, our study further supports the antiviral roles of RNAi in mammalian cells. However, the 22-nt vsRNAs stimulated by SARS-CoV-2 infection in Vero cells appeared to be in low amount (**Figure 4**), giving us a caution that to what extent these vsRNAs may function in antiviral RNAi. Besides, as our findings are limited to Vero cells which are isolated from kidney epithelial cells of African green monkey, whether similar pattern occurs in SARS-CoV-2 infected human cell lines needs to be elucidated. Recent study reported the life-threatening pneumonia caused by SARS-CoV-2 of an unvaccinated child with inborn ISG-IRF9-deficiency, which resulted from impaired type I and III interferon immunity (80). Our study reflects the antiviral response that may happen when the critical antiviral IFN pathway is in absence, this would provide considerable practical implications for the clinical treatment of SARS-CoV-2.

## Materials and methods

### Cell culture and virus infection

African green monkey kidney epithelial Vero (ATCC, CCL-81) were maintained at 37°C, 5% CO_2_ in Dulbecco’s modified Eagle’s medium (DMEM, Gibco) with 10% fetal bovine serum (FBS, HyClone) and penicillin-streptomycin (GENOM). SARS-CoV-2, strain code IPBCAMS_BJ95 (https://ngdc.cncb.ac.cn/, accession No: GVHAORV01000000), was isolated from a pharyngeal swab from a COVID-19 patient. Virus titer was determined by using the plaque assay with Vero cells. For SARS-CoV-2 infection, the Vero cells were briefly washed with DMEM at 16 h after seeding, and incubated with SARS-CoV-2 for 1 h at a multiplicity of infection (MOI) of 0.1, then supplemented with DMEM maintenance medium containing 1% BSA and penicillin-streptomycin. Cells were cultured at 37°C in a 5% CO_2_ incubator for additional 6 and 12 hours. Cultured cells were washed twice with PBS before collection. All experiments involving live SARS-CoV-2 were performed in Biosafety Level 3 (BSL-3) facility.

### RNA preparation for sRNA sequencing

Total RNA was isolated from non-infected and infected (6 hpi and 12 hpi) Vero cell according to the previous methods with mere modification. In brief, using TRIzol reagent (Thermo Fisher) in BSL-3 laboratory following the manufacturer’s instruction. In brief, 2.5 × 10^5^ infected cells were lysed with 1 mL TRIzol reagent, mixed with 400 μL chloroform then vortexed for 1 min. After incubation on ice for 5 min, the mixture was centrifuged at 12,000 × g (at 4°C) for 20 min. The aqueous layer was extracted and mixed with 2.5 volume of ethanol for precipitation at −20°C overnight, followed by recovering through centrifugation, washing with 75% ethanol, air-drying and dissolving with DEPC-treated water.

After quality evaluation by Nanodrop spectrophotometer, the integrity of RNA concentration was subsequently examined on bioanalyzer. With the RNA integrity number (RIN) > 7.0, RNA concentration was utilized for sRNA library preparation. In general, 1 μg of total RNA was used for single library construction using NEBNext^®^ Multiplex Small RNA Library Prep Set for Illumina (NEB) following the manufacturer’s instructions. All sRNA libraries were prepared in biological duplicates. The libraries were qualified by Agilent 2100 Bioanalyzer system and then sequenced on NextseqCN500 Illumina platform.

### SRNA sequencing

Raw reads were trimmed of adapters by SPORTS1.1 (with the parameter sports.pl -i -p -g -m -r -t -w -o -k -L 50 -M 1 -e -f -a -y AGATCGGAAGAGCACACGTCTGAACTCCAGTCAC) to remove low quality reads (81). Cleaned reads were mapped to human (Build version GRCh38, NCBI) reference genome with bowtie v0.12.7 with parameters allowing 1-bp mismatch (-v 1 -k 10 -S -t –p). Reads that did not align to human genome were mapped to SARS-CoV-2 (assembly Jul.2020/NC_045512.2, NCBI) reference genome using bowtie with parameters (-v 1 -k 10 -S -t –p). Mapped reads on sense and antisense strands were extracted to perform downstream analyses.

### The differentially expressed miRNA analysis

The expression of miRNAs was measured using featureCounts v2.0.1 in subread with standard parameters (82) using annotations from miRbase (83). miRNA expression levels were normalized as counts per million (CPM) of total aligned miRNA reads. The CPM level was used to measure the expression level of each mature miRNA. DEseq2 v1.32.0 in R package was applied for identifying differentially expressed miRNAs (84). miRNAs with fold change ≥ 1.5, adjusted *P* value ≤ 0.05 were recognized as DE miRNAs.

### Targets prediction of the DE miRNAs

We used mirDIP (http://ophid.utoronto.ca/mirDIP/) to predict human target genes of DE miRNAs based on the sequence matching and free energy assessment of miRNA-3’UTR binding (85). The prediction parameters were performed as follows, score class-Very High, integrated Score ≥ 0.6. The overlapped results from multiple databases were considered as DE miRNA targets.

### Gene ontology and KEGG pathway analysis of target genes

For better understanding of the function miRNAs played through targeting human genome, Gene Ontology (GO) and the Kyoto Encyclopedia of Genes and Genomes (KEGG) analysis of target genes were performed through the clusterProfiler v4.0.5 package in R with adjusted *P* value ≤ 0.05 as threshold for significance.

### Size distribution

The size distribution of virus-derived sRNAs were performed using in-house python script. Reads mapping to human genome and viral genome of SARS-CoV-2 were extracted by SAMtools v1.9 (86) and computed with python script. For host small RNAs, mapped reads range from 18 nt to 28 nt were displayed in bar plot. For virus-derived sRNAs, mapped reads range from 18 nt to 35 nt were displayed in bar plot.

## Data availability statement

The data presented in the study are deposited in the National Center for Biotechnology Information SRA, accession number SRR18355593-SRR18355600, or BioProject, accession number, PRJNA792694, (https://www.ncbi.nlm.nih.gov/bioproject/PRJNA792694). All study data are available in the main text or supplementary materials.

## Author contributions

XMZ, LR, and XJZ initiated the project; XMZ, LR, and JC designed the experiments; and JR and LC performed virus culture in biosafety level 3 lab and conducted the experiments; YM, YL, LF, and JG performed the bioinformatic analysis. XMZ, JC, LR, YL, JR, XJZ, QL, and XY wrote the paper. All authors have read and approved the manuscript for publication. YL, JR, and YM contributed equally to this work. All authors contributed to the article and approved the submitted version.

## Funding

This work is supported by the National Key Plan for Scientific Research and Development of China (2019YFC1200504), National Natural Science Foundation of China (NSFC 32090012 and 31900224), Beijing Municipal Natural Science Foundation (5202017), National Key Plan for Scientific Research and Development of China (2021YFC2600103), Strategic Priority Research program of the CAS (XDPB16), National Natural Science Foundation of China (91954105), Programs of CAS (ZDBS-LY-SM027 and KJZD-SW-L11), and Open Research Fund Program of The State Key Laboratory of Integrated Management of Pest Insects and Rodents (ChineseIPM2007 and IPM2108).

## Acknowledgment

We thank YLi, YLiu, Jianfei Shi, and Haiyan Xu (Institute of Zoology, Chinese Academy of Sciences, Beijing, China) for constructive comments.

## Conflict of interest

The authors declare that the research was conducted in the absence of any commercial or financial relationships that could be construed as a potential conflict of interest.

## Publisher’s note

All claims expressed in this article are solely those of the authors and do not necessarily represent those of their affiliated organizations, or those of the publisher, the editors and the reviewers. Any product that may be evaluated in this article, or claim that may be made by its manufacturer, is not guaranteed or endorsed by the publisher.

## Supplementary material

Supplementary Figure 1 Description of sRNA libraries in control and SARS-CoV-2-infected Vero cells. (A) Correlation of miRNA reads between two biological replicates in control and SARS-CoV-2-infected (6 hpi/12 hpi) Vero cells. (B) PCA plot of miRNA-seq libraries of control and virus-infected (6 hpi/12 hpi) Vero cells.

Supplementary Figure 2 The major length of host small RNAs without miR-21 concentrates in 22 and 23 nt. (A) Size distribution of total small RNAs without 22 and 23-nt miR-21 isoforms in control and virus-infected (6 hpi/12 hpi) Vero cells. Clean reads are aligned to the human reference. Mapped reads without matching to tRNA and rRNA are analyzed to remove 22 and 23-nt miR-21 isoforms. The remaining sRNAs are classified by length and are shown as read counts. Pie plots represent sRNAs initiated with A/U/C/G in control and virus-infected Vero cells. (B) Size distribution of host miRNAs without 22 and 23-nt miR-21 isoforms in control and virus-infected (6 hpi/12 hpi) Vero cells. The population of miRNAs are classified by length and are shown as read counts. Pie plots represent miRNAs initiated with A/U/C/G in control and virus-infected Vero cells.

Supplementary Figure 3 The accumulation of vsRNAs along SARS-CoV-2 genome in Vero cell line. **(A)** Distribution of 18-35 nt virus-derived sRNAs mapping to positive and negative strand of SARS-CoV-2 genome in control and virus-infected (6 hpi/12 hpi) Vero cells. The small RNA abundance is presented as reads counts along the viral genome (n=2). **(B)** Distribution of 22-nt virus-derived sRNAs mapping to positive and negative strand of SARS-CoV-2 genome in control and virus-infected (6 hpi/12 hpi) Vero cells. The 22-nt small RNA abundance is presented as reads counts along the viral genome. Arrows represent the locus where read sequences are analyzed (See **Figure 4B**) (n=2).

Supplementary Figure 4 The size distribution of vsRNAs along viral genome in SARS-CoV-2 infected cell lines. **(A)** Size distribution and abundance of 18-35 nt vsRNAs in SARS-CoV-2 infected Calu-3, PC-9, A549-hACE2 cells at different post-infection time points from Pawlica et al., 2021. Pie plots represent vsRNAs initiated with A/U/C/G at positive and negative strands. **(B)** Size distribution and abundance of 18-35 nt vsRNAs in SARS-CoV-2 infected A549-hACE2 cells and Calu-3 cells at different post-infection time points from Singh et al., 2022 and Meng et al., 2021. Pie plots represent vsRNAs initiated with A/U/C/G at negative strand. **(C)** Size distribution and abundance of 18-35 nt vsRNAs in SARS-CoV-2 infected Calu-3 at 4, 12, 24 hpi from Wyler et al., 2021. Pie plots represent vsRNAs initiated with A/U/C/G at positive and negative strands. **(D)** Size distribution and abundance of 18-35 nt vsRNAs in SARS-CoV-2 infected Calu-3 at 24, 36 and 48 hpi from Kim et al., 2021. Pie plots represent vsRNAs initiated with A/U/C/G in positive and negative strands.

Supplementary Figure 5 SINV forms continuous 20-nt perfect complementary base pairs with 2-nt 3’ overhang. Read sequences along the 5’-terminal 120 nt sequence of SINV genome. Read counts (in brackets), read length and genomic position are indicated and mismatched bases are **mentioned in red.**

## References

[1] Chen Y Guo Y Pan YH Zhao ZJ . Structure analysis of the receptor binding of 2019-nCoV. Biochem Biophys Res Commun (2020) 525(1):135–40. doi: 10.1016/j.bbrc.2020.02.071 PMC7092824 32081428

[2] Munster VJ Koopmans M van Doremalen N van Riel D de Wit E . A novel coronavirus emerging in China - key questions for impact assessment. New Engl J Med (2020) 382(8):692–4. doi: 10.1056/NEJMp2000929 31978293

[3] Wang C Horby PW Hayden FG Gao GF . A novel coronavirus outbreak of global health concern. Lancet (2020) 395(10223):470–3. doi: 10.1016/S0140-6736(20)30185-9 PMC7135038 31986257

[4] Wu F Zhao S Yu B Chen YM Wang W Song ZG . A new coronavirus associated with human respiratory disease in China. Nature (2020) 579(7798):265–9. doi: 10.1038/s41586-020-2202-3 PMC7094943 32015508

[5] Hoffmann M Kleine-Weber H Schroeder S Kruger N Herrler T Erichsen S . SARS-CoV-2 cell entry depends on ACE2 and TMPRSS2 and is blocked by a clinically proven protease inhibitor. Cell (2020) 181(2):271–280.e8. doi: 10.1016/j.cell.2020.02.052 32142651 PMC7102627

[6] Helms J Meziani F . Neurologic features in severe SARS-CoV-2 infection. New Engl J Med (2020) 382(23):2268–70. doi: 10.1056/NEJMc2008597 PMC7179967 32294339

[7] Pleasure SJ Green AJ Josephson SA . The spectrum of neurologic disease in the severe acute respiratory syndrome coronavirus 2 pandemic infection neurologists move to the frontlines. JAMA Neurol (2020) 77(6):679–80. doi: 10.1001/jamaneurol.2020.1065 32275291

[8] Zhang C Shi L Wang F-S . Liver injury in COVID-19: management and challenges. Lancet Gastroenterol Hepatol (2020) 5(5):428–30. doi: 10.1016/S2468-1253(20)30057-1 PMC7129165 32145190

[9] Goyal P Choi JJ Safford MM . Clinical characteristics of covid-19 in new York city. New Engl J Med (2020) 382(24):2372–4. doi: 10.1056/NEJMc2010419 PMC7182018 32302078

[10] Huang C Wang Y Li X . Clinical features of patients infected with 2019 novel coronavirus in wuhan, China. Lancet (2020) 395(10223):496–6. doi: 10.1016/S0140-6736(20)30183-5 PMC7159299 31986264

[11] Bornstein SR Gallwitz B Kellerer M Ludwig B Muller-Wieland D Neu A . Practical recommendations for diabetes management in patients with COVID-19 disease. Diabetol Und Stoffwechsel (2020) 15(3):241–6. doi: 10.1055/a-1159-1486

[12] Goubau D Deddouche S Reis e Sousa C . Cytosolic sensing of viruses. Immunity (2013) 38(5):855–69. doi: 10.1016/j.immuni.2013.05.007 PMC7111113 23706667

[13] Wu J Chen ZJ . Innate immune sensing and signaling of cytosolic nucleic acids. Annu Rev Immunol (2014) 32:461–88. doi: 10.1146/annurev-immunol-032713-120156 24655297

[14] Schlee M Hartmann G . Discriminating self from non-self in nucleic acid sensing. Nat Rev Immunol (2016) 16(9):566–80. doi: 10.1038/nri.2016.78 PMC7097691 27455396

[15] McFadden MJ Gokhale NS Horner SM . Protect this house: cytosolic sensing of viruses. Curr Opin Virol (2017) 22:36–43. doi: 10.1016/j.coviro.2016.11.012 27951430 PMC5346041

[16] Schneider WM Chevillotte MD Rice CM . Interferon-stimulated genes: A complex web of host defenses. Annu Rev Immunol (2014) 32:513–45. doi: 10.1146/annurev-immunol-032713-120231 PMC4313732 24555472

[17] Maillard PV van der Veen AG Poirier EZ Sousa CRE . Slicing and dicing viruses: antiviral RNA interference in mammals. EMBO J (2019) 38(8):e100941. doi: 10.15252/embj.2018100941 30872283 PMC6463209

[18] Guo Z Li Y Ding S-W . Small RNA-based antimicrobial immunity. Nat Rev Immunol (2019) 19(1):31–44. doi: 10.1038/s41577-018-0071-x 30301972

[19] Kim VN Han J Siomi MC . Biogenesis of small RNAs in animals. Nat Rev Mol Cell Biol (2009) 10(2):126–39. doi: 10.1038/nrm2632 19165215

[20] Kincaid RP Sullivan CS . Virus-encoded microRNAs: An overview and a look to the future. PloS Pathog (2012) 8(12):e1003018. doi: 10.1371/journal.ppat.1003018 23308061 PMC3534370

[21] Wang M Yu F Wu W Wang Y Ding H Qian LL . Epstein-Barr Virus-encoded microRNAs as regulators in host immune responses. Int J Biol Sci (2018) 14(5):565–76. doi: 10.7150/ijbs.24562 PMC5968849 29805308

[22] Huang J Yang M Zhang X . The function of small RNAs in plant biotic stress response. J Integr Plant Biol (2016) 58(4):312–27. doi: 10.1111/jipb.12463 26748943

[23] Berkhout B . RNAi-mediated antiviral immunity in mammals. Curr Opin Virol (2018) 32:9–14. doi: 10.1016/j.coviro.2018.07.008 30015014

[24] Ding SW Han QX Wang JY Li WX . Antiviral RNA interference in mammals. Curr Opin Immunol (2018) 54:109–14. doi: 10.1016/j.coi.2018.06.010 PMC6196099 30015086

[25] Billy E Brondani V Zhang HD Muller U Filipowicz W . Specific interference with gene expression induced by long, double-stranded RNA in mouse embryonal teratocarcinoma cell lines. Proc Natl Acad Sci USA (2001) 98(25):14428–33. doi: 10.1073/pnas.261562698 PMC64698 11724966

[26] Poirier EZ Buck MD Chakravarty P Carvalho J Frederico B Cardoso A . An isoform of dicer protects mammalian stem cells against multiple RNA viruses. Science (2021) 373(6551):231–6. doi: 10.1126/science.abg2264 PMC7611482 34244417

[27] Maillard PV Ciaudo C Marchais A Li Y Jay F Ding SW . Antiviral RNA interference in mammalian cells. Science (2013) 342(6155):235–8. doi: 10.1126/science.1241930 PMC3853215 24115438

[28] van der Veen AG Maillard PV Schmidt JM Lee SA Deddouche-Grass S Borg A . The RIG-i-like receptor LGP2 inhibits dicer-dependent processing of long double-stranded RNA and blocks RNA interference in mammalian cells. EMBO J (2018) 37(4):e97479. doi: 10.15252/embj.201797479 29351913 PMC5813259

[29] Zhang YQ Xu Y Dai YP Li Z Wang JX Ye Z . Efficient dicer processing of virus-derived double-stranded RNAs and its modulation by RIG-i-like receptor LGP2. PloS Pathog (2021) 17(8):e1009790. doi: 10.1371/journal.ppat.1009790 34343211 PMC8362961

[30] Maillard PV Van der Veen AG Deddouche-Grass S Rogers NC Merits A Sousa CRE . Inactivation of the type I interferon pathway reveals long double-stranded RNA-mediated RNA interference in mammalian cells. EMBO J (2016) 35(23):2505–18. doi: 10.15252/embj.201695086 PMC5167344 27815315

[31] Meng F Siu GKH Mok BWY Sun JH Fung KSC Lam JYW . Viral MicroRNAs encoded by nucleocapsid gene of SARS-CoV-2 are detected during infection, and targeting metabolic pathways in host cells. Cells (2021) 10(7):1762. doi: 10.3390/cells10071762 34359932 PMC8307234

[32] Singh M Chazal M Quarato P Bourdon L Malabat C Vallet T . A virus-derived microRNA targets immune response genes during SARS-CoV-2 infection. EMBO Rep (2021) 23(2):e54341. doi: 10.15252/embr.202154341 34914162 PMC8811647

[33] Pawlica P Yario TA White S Wang JH Moss WN Hui P . SARS-CoV-2 expresses a microRNA-like small RNA able to selectively repress host genes. Proc Natl Acad Sci USA (2021) 118(52):e2116668118. doi: 10.1073/pnas.2116668118 34903581 PMC8719879

[34] Zhang Q Bastard P Liu ZY Le Pen J Moncada-Velez M Chen J . Inborn errors of type I IFN immunity in patients with life-threatening COVID-19. Science (2020) 370(6515):eabd4570. doi: 10.1126/science.abd4570 32972995 PMC7857407

[35] Desmyter J Melnick JL Rawls WE . Defectiveness of interferon production and of rubella virus interference in a line of African green monkey kidney cells (Vero). J Virol (1968) 2(10):955–61. doi: 10.1128/jvi.2.10.955-961.1968 PMC375423 4302013

[36] Girardi E Chane-Woon-Ming B Messmer M Kaukinen P Pfeffer S . Identification of RNase l-dependent, 3 '-End-Modified, viral small RNAs in sindbis virus-infected mammalian cells. Mbio (2013) 4(6):e00698–13. doi: 10.1128/mBio.00698-13 PMC3870239 24255120

[37] Zeng JX Luo ZF Dong SP Xie XC Liang XY Yan YZ . Functional mapping of AGO-associated zika virus-derived small interfering RNAs in neural stem cells. Front Cell Infect Microbiol (2021) 11:628887. doi: 10.3389/fcimb.2021.628887 33718276 PMC7946837

[38] Zeng JX Dong SP Luo ZF Xie XC Fu BS Li P . The zika virus capsid disrupts corticogenesis by suppressing dicer activity and miRNA biogenesis. Cell Stem Cell (2020) 27(4):618–32. doi: 10.1016/j.stem.2020.07.012 PMC7541724 32763144

[39] Dang JW Tiwari SK Qin Y Rana TM . Genome-wide integrative analysis of zika-Virus-Infected neuronal stem cells reveals roles for MicroRNAs in cell cycle and stemness. Cell Rep (2019) 27(12):3618–28. doi: 10.1016/j.celrep.2019.05.059 PMC6687627 31216479

[40] Zhang C Liu C Jiang L Cui LB Li CY Song GX . Verification of SARS-CoV-2-Encoded small RNAs and contribution to infection-associated lung inflammation. Chinese Medical J (2022), 135(15):1858–1860. doi: 10.1097/CM9.0000000000002059 PMC9521766 35838380

[41] Boele J Persson H Shin JW Ishizu Y Newie IS Sokilde R . PAPD5-mediated 3 ' adenylation and subsequent degradation of miR-21 is disrupted in proliferative disease. Proc Natl Acad Sci United States America (2014) 111(31):11467–72. doi: 10.1073/pnas.1317751111 PMC4128123 25049417

[42] Peng S Wang J Wei ST Li CF Zhou K Hu J . Endogenous cellular MicroRNAs mediate antiviral defense against influenza a virus. Mol Therapy-Nucleic Acids (2018) 10:361–75. doi: 10.1016/j.omtn.2017.12.016 PMC5862538 29499948

[43] Hao Q Wang QH Qian HZ Jiang J Liu X Xia W . Identification and functional characterization of miR-451a as a novel plasma-based biomarker for occult hepatitis b virus infection. Microbial Pathog (2021) 161(Pt A):105233. doi: 10.1016/j.micpath.2021.105233 34626767

[44] Bamunuarachchi G Yang XY Huang CQ Liang YR Guo YJ Liu L . MicroRNA-206 inhibits influenza a virus replication by targeting tankyrase 2. Cell Microbiol (2021) 23(2):e13281. doi: 10.1111/cmi.13281 33099847 PMC8279255

[45] Seong R-K Lee JK Cho GJ Kumar M Shin OS . mRNA and miRNA profiling of zika virus-infected human umbilical cord mesenchymal stem cells identifies miR-142-5p as an antiviral factor. Emerg Microbes Infect (2020) 9(1):2061–75. doi: 10.1080/22221751.2020.1821581 PMC7534337 32902370

[46] Choi E-J Kim HB Baek YH Kim EH Pascua PNQ Park SJ . Differential microRNA expression following infection with a mouse-adapted, highly virulent avian H5N2 virus. BMC Microbiol (2014) 14:252. doi: 10.1186/s12866-014-0252-0 25266911 PMC4189662

[47] Liang ZP Wang L Wu H Singh D Zhang XX . Integrative analysis of microRNA and mRNA expression profiles in MARC-145 cells infected with PRRSV. Virus Genes (2020) 56(5):610–20. doi: 10.1007/s11262-020-01786-w 32785889

[48] Scheller N Herold S Kellner R Bertrams W Jung AL Janga H . Proviral MicroRNAs detected in extracellular vesicles from bronchoalveolar lavage fluid of patients with influenza virus-induced acute respiratory distress syndrome. J Infect Dis (2019) 219(4):540–3. doi: 10.1093/infdis/jiy554 30239899

[49] Xu R Yu SS Yao RR Tang RC Liang JW Pang XW . Interferon-inducible LINC02605 promotes antiviral innate responses by strengthening IRF3 nuclear translocation. Front Immunol (2021) 12:755512. doi: 10.3389/fimmu.2021.755512 34804040 PMC8602795

[50] De Gonzalo-Calvo D Benitez ID Pinilla L Carratala A Moncusi-Moix A Gort-Paniello C . Circulating microRNA profiles predict the severity of COVID-19 in hospitalized patients. Trans Res (2021) 236:147–59. doi: 10.1016/j.trsl.2021.05.004 PMC8149473 34048985

[51] Fayyad-Kazan M Makki R Skafi N El Homsi M Hamade A El Majzoub R . Circulating miRNAs: Potential diagnostic role for coronavirus disease 2019 (COVID-19). Infect Genet Evol (2021) 94:105020. doi: 10.1016/j.meegid.2021.105020 34343725 PMC8325559

[52] Vasuri F Ciavarella C Collura S Mascoli C Valente S Degiovanni A . Adventitial microcirculation is a major target of SARS-CoV-2-Mediated vascular inflammation. Biomolecules (2021) 11(7):1063. doi: 10.3390/biom11071063 34356687 PMC8301851

[53] Sardar R Satish D Gupta D . Identification of novel SARS-CoV-2 drug targets by host MicroRNAs and transcription factors Co-regulatory interaction network analysis. Front Genet (2020) 11:571274. doi: 10.3389/fgene.2020.571274 33173539 PMC7591747

[54] Meloche S Pouyssegur J . The ERK1/2 mitogen-activated protein kinase pathway as a master regulator of the G1- to s-phase transition. Oncogene (2007) 26(22):3227–39. doi: 10.1038/sj.onc.1210414 17496918

[55] Yang HW Chung M Kudo T Meyer T . Competing memories of mitogen and p53 signalling control cell-cycle entry. Nature (2017) 549(7672):404–8. doi: 10.1038/nature23880 PMC6544019 28869970

[56] Peltier J O'Neill A Schaffer DV . PI3K/Akt and CREB regulate adult neural hippocampal progenitor proliferation and differentiation. Dev Neurobiol (2007) 67(10):1348–61. doi: 10.1002/dneu.20506 17638387

[57] Zhang Q Gong R Qu J Zhou YJ Liu WY Chen MZ . Activation of the Ras/Raf/MEK pathway facilitates hepatitis c virus replication via attenuation of the interferon-JAK-STAT pathway. J Virol (2012) 86(3):1544–54. doi: 10.1128/JVI.00688-11 PMC3264379 22114332

[58] Frank F Sonenberg N Nagar B . Structural basis for 5 '-nucleotide base-specific recognition of guide RNA by human AGO2. Nature (2010) 465(7299):818–22. doi: 10.1038/nature09039 20505670

[59] Li Y Basavappa M Lu J Dong S Cronkite DA Prior JT . Induction and suppression of antiviral RNA interference by influenza a virus in mammalian cells. Nat Microbiol (2017) 2(3):16250. doi: 10.1038/nmicrobiol.2016.250 PMC5488270 27918527

[60] Wyler E Mosbauer K Franke V Diag A Gottula LT Arsie R . Transcriptomic profiling of SARS-CoV-2 infected human cell lines identifies HSP90 as target for COVID-19 therapy. Iscience (2021) 24(3):102151. doi: 10.1016/j.isci.2021.102151 33585804 PMC7866843

[61] Kim D Kim S Park J Chang HR Chang J Ahn J . A high-resolution temporal atlas of the SARS-CoV-2 translatome and transcriptome. Nat Commun (2021) 12(1):5120. doi: 10.1038/s41467-021-25361-5 34433827 PMC8387416

[62] Zhang Y Li Z Ye Z Xu Y Wang BB Wang CC . The activation of antiviral RNA interference not only exists in neural progenitor cells but also in somatic cells in mammals. Emerg Microbes Infect (2020) 9(1):1580–9. doi: 10.1080/22221751.2020.1787798 PMC7473182 32576094

[63] Xu YP Qiu Y Zhang BY Chen GL Chen Q Wang M . Zika virus infection induces RNAi-mediated antiviral immunity in human neural progenitors and brain organoids. Cell Res (2019) 29(4):265–73. doi: 10.1038/s41422-019-0152-9 PMC6461993 30814679

[64] Hanna JA Garcia MR Lardennois A Leavey PJ Maglic D Fagnan A . PAX3-FOXO1 drives miR-486-5p and represses miR-221 contributing to pathogenesis of alveolar rhabdomyosarcoma. Oncogene (2018) 37(15):1991–2007. doi: 10.1038/s41388-017-0081-3 29367756 PMC5895609

[65] Lopez-Bertoni H Kotchetkov IS Mihelson N Lal B Rui Y Ames H . A Sox2:miR-486-5p axis regulates survival of GBM cells by inhibiting tumor suppressor networks. Cancer Res (2020) 80(8):1644–55. doi: 10.1158/0008-5472.CAN-19-1624 PMC7165043 32094299

[66] Yang P Zhao YZ Li J Liu CY Zhu LN Zhang J . Downregulated miR-451a as a feature of the plasma cfRNA landscape reveals regulatory networks of IL-6/IL-6R-associated cytokine storms in COVID-19 patients. Cell Mol Immunol (2021) 18(4):1064–6. doi: 10.1038/s41423-021-00652-5 PMC7907785 33637960

[67] Tang B Lei BA Qi GY Liang XS Tang F Yuan SG . MicroRNA-155-3p promotes hepatocellular carcinoma formation by suppressing FBXW7 expression. J Exp Clin Cancer Res (2016) 35(1):93. doi: 10.1186/s13046-016-0371-6 27306418 PMC4910248

[68] Wu X Wang YY Yu TF Nie E Hu Q Wu WN . Blocking MIR155HG/miR-155 axis inhibits mesenchymal transition in glioma. Neuro-Oncology (2017) 19(9):1195–205. doi: 10.1093/neuonc/nox017 PMC5570208 28371892

[69] Zhu Z Qi YH Ge AH Zhu YF Xu K Ji H . Comprehensive characterization of serum MicroRNA profile in response to the emerging avian influenza a (H7N9) virus infection in humans. Viruses-Basel (2014) 6(4):1525–39. doi: 10.3390/v6041525 PMC4014708 24699363

[70] Kindrachuk J Ork B Hart BJ Mazur S Holbrook MR Frieman MB . Antiviral potential of ERK/MAPK and PI3K/AKT/mTOR signaling modulation for middle East respiratory syndrome coronavirus infection as identified by temporal kinome analysis. Antimicrobial Agents Chemother (2015) 59(2):1088–99. doi: 10.1128/AAC.03659-14 PMC4335870 25487801

[71] Wong WR Chen YY Yang SM Chen YL Horng JT . Phosphorylation of PI3K/Akt and MAPK/ERK in an early entry step of enterovirus 71. Life Sci (2005) 78(1):82–90. doi: 10.1016/j.lfs.2005.04.076 16150462 PMC7094582

[72] Scaturro P Stukalov A Haas DA Cortese M Draganova K Plaszczyca A . An orthogonal proteomic survey uncovers novel zika virus host factors. Nature (2018) 561(7722):253–7. doi: 10.1038/s41586-018-0484-5 30177828

[73] Rabaan AA Al-Ahmed SH Haque S Sah R Tiwari R Malik YS . SARS-CoV-2, SARS-CoV, and MERS-COV: A comparative overview. Le infezioni med (2020) 28(2):174–84. 32275259

[74] Basile MS Cavalli E McCubrey J Hernandez-Bello J Munoz-Valle JF Fagone P . The PI3K/Akt/mTOR pathway: A potential pharmacological target in COVID-19. Drug Discov Today (2021) 27(3):848–56. doi: 10.1016/j.drudis.2021.11.002 PMC8574122 34763066

[75] Li Y Lu JF Han YH Fan XX Ding SW . RNA Interference functions as an antiviral immunity mechanism in mammals. Science (2013) 342(6155):231–4. doi: 10.1126/science.1241911 PMC3875315 24115437

[76] Yuen CK Lam JY Wong WM Mak LF Wang XH Chu H . SARS-CoV-2 nsp13, nsp14, nsp15 and orf6 function as potent interferon antagonists. Emerg Microbes Infect (2020) 9(1):1418–28. doi: 10.1080/22221751.2020.1780953 PMC7473193 32529952

[77] Xia HJ Cao ZG Xie XP Zhang XW Chen JYC Wang HL . Evasion of type I interferon by SARS-CoV-2. Cell Rep (2020) 33(1):108234. doi: 10.1016/j.celrep.2020.108234 32979938 PMC7501843

[78] Yin J Gardner CL Burke CW Ryman KD Klimstra WB . Similarities and differences in antagonism of neuron Alpha/Beta interferon responses by Venezuelan equine encephalitis and sindbis alphaviruses. J Virol (2009) 83(19):10036–47. doi: 10.1128/JVI.01209-09 PMC2748036 19641001

[79] Xia HJ Luo HL Shan C Muruato AE Nunes BTD Medeiros DBA . An evolutionary NS1 mutation enhances zika virus evasion of host interferon induction. Nat Commun (2018) 9(1):414. doi: 10.1038/s41467-017-02816-2 29379028 PMC5788864

[80] Levy R Zhang P Bastard P Dorgham K Melki I Hadchouel A . Monoclonal antibody-mediated neutralization of SARS-CoV-2 in an IRF9-deficient child. Proc Natl Acad Sci USA (2021) 118(45):e2114390118. doi: 10.1073/pnas.2114390118 34702736 PMC8609338

[81] Shi JC Ko EA Sanders KM Chen Q Zhou T. SPORTS1.0: A tool for annotating and profiling non-coding RNAs optimized for rRNA- and tRNA-derived small RNAs. Genomics Proteomics Bioinf (2018) 16(2):144–51. doi: 10.1016/j.gpb.2018.04.004 PMC6112344 29730207

[82] Liao Y Smyth GK Shi W . featureCounts: an efficient general purpose program for assigning sequence reads to genomic features. Bioinformatics (2014) 30(7):923–30. doi: 10.1093/bioinformatics/btt656 24227677

[83] Kozomara A Griffiths-Jones S . miRBase: integrating microRNA annotation and deep-sequencing data. Nucleic Acids Res (2011) 39:D152–7. doi: 10.1093/nar/gkq1027 PMC3013655 21037258

[84] Love MI Huber W Anders S . Moderated estimation of fold change and dispersion for RNA-seq data with DESeq2. Genome Biol (2014) 15(12):550. doi: 10.1186/s13059-014-0550-8 25516281 PMC4302049

[85] Tokar T Pastrello C Rossos AEM Abovsky M Hauschild AC Tsay M . mirDIP 4.1-integrative database of human microRNA target predictions. Nucleic Acids Res (2018) 46(D1):D360–70. doi: 10.1093/nar/gkx1144 PMC5753284 29194489

[86] Li H Handsaker B Wysoker A Fennell T Ruan J Homer N . The sequence Alignment/Map format and SAMtools. Bioinformatics (2009) 25(16):2078–9. doi: 10.1093/bioinformatics/btp352 PMC2723002 19505943

