# Supplementary figures and images for: SARS-CoV-2 RNAs are processed into 22-nt vsRNAs in Vero cells
Source: Front Immunol. 2022 Oct 28;13:1008084. doi: 10.3389/fimmu.2022.1008084 (PMC9650353; doi:10.3389/fimmu.2022.1008084)

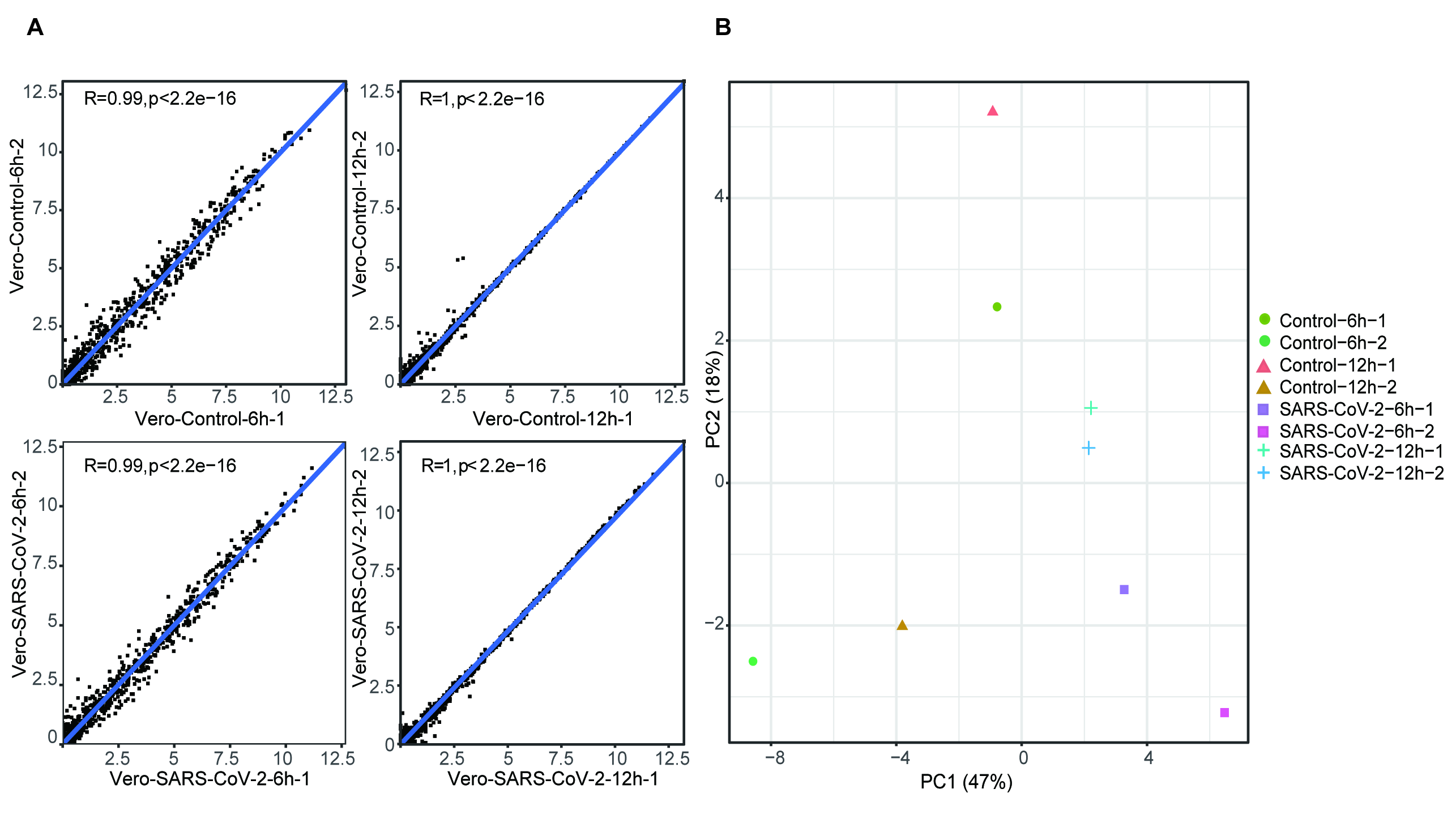

Supplement: Supplementary Figure 1 — Description of sRNA libraries in control and SARS-CoV-2-infected Vero cells. (A) Correlation of miRNA reads between two biological replicates in control and SARS-CoV-2-infected (6 hpi/12 hpi) Vero cells. (B) PCA plot of miRNA-seq libraries of control and virus-infected (6 hpi/12 hpi) Vero cells. [file DataSheet_1.zip › Data Sheet 1/Figure S1.tif]

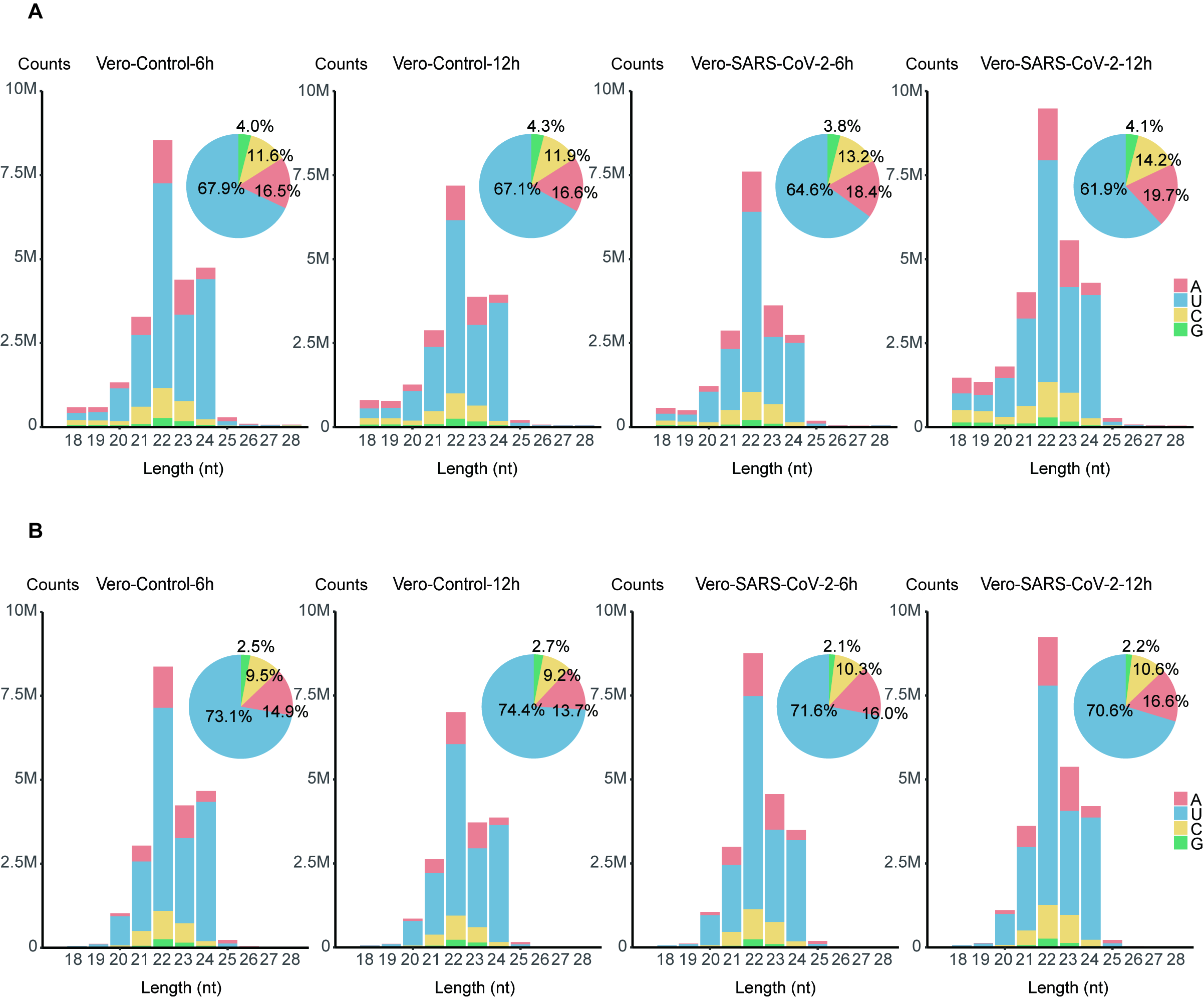

Supplement: Supplementary Figure 1 — Description of sRNA libraries in control and SARS-CoV-2-infected Vero cells. (A) Correlation of miRNA reads between two biological replicates in control and SARS-CoV-2-infected (6 hpi/12 hpi) Vero cells. (B) PCA plot of miRNA-seq libraries of control and virus-infected (6 hpi/12 hpi) Vero cells. [file DataSheet_1.zip › Data Sheet 1/Figure S2.tif]

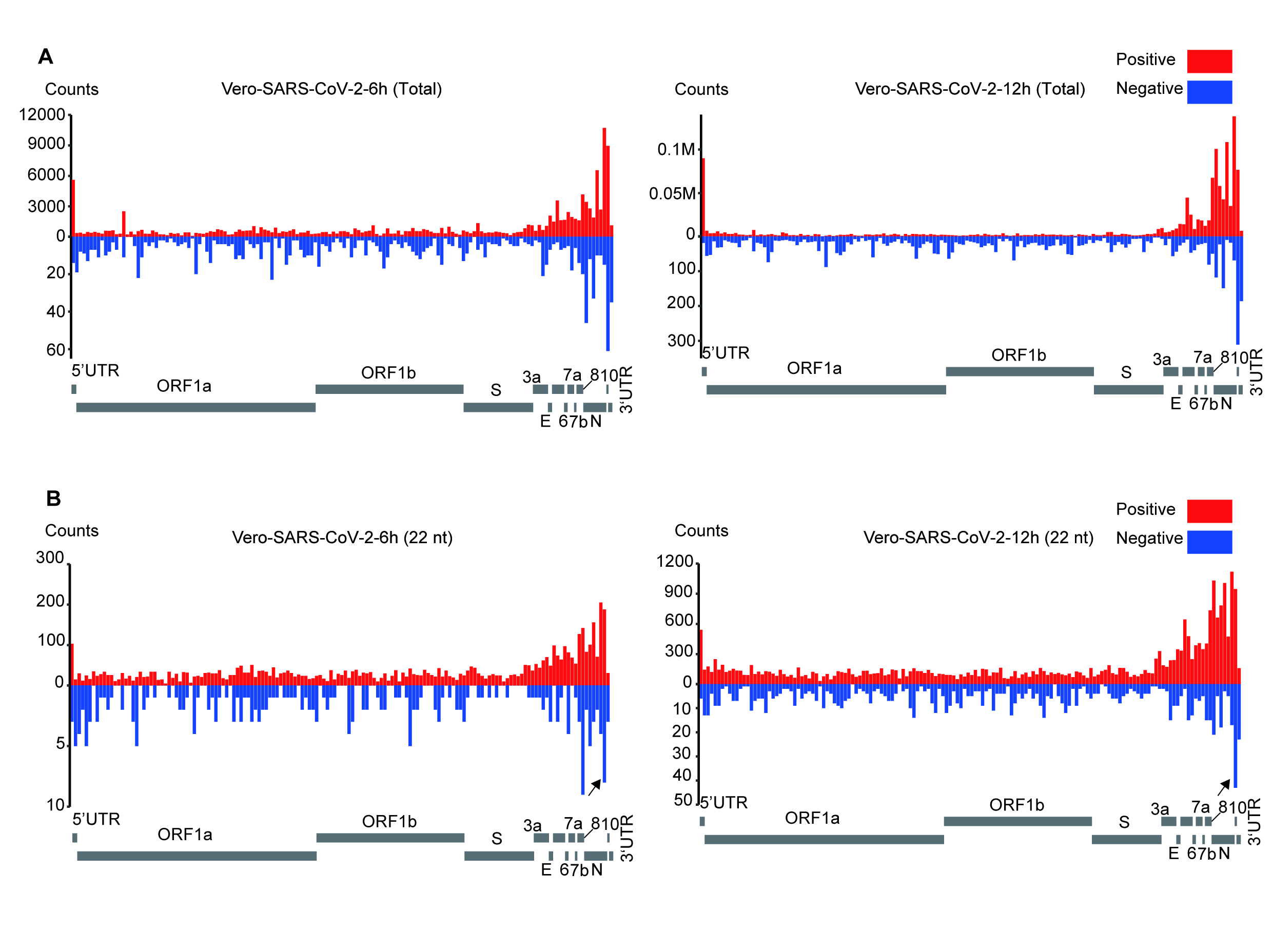

Supplement: Supplementary Figure 1 — Description of sRNA libraries in control and SARS-CoV-2-infected Vero cells. (A) Correlation of miRNA reads between two biological replicates in control and SARS-CoV-2-infected (6 hpi/12 hpi) Vero cells. (B) PCA plot of miRNA-seq libraries of control and virus-infected (6 hpi/12 hpi) Vero cells. [file DataSheet_1.zip › Data Sheet 1/Figure S3.tif]

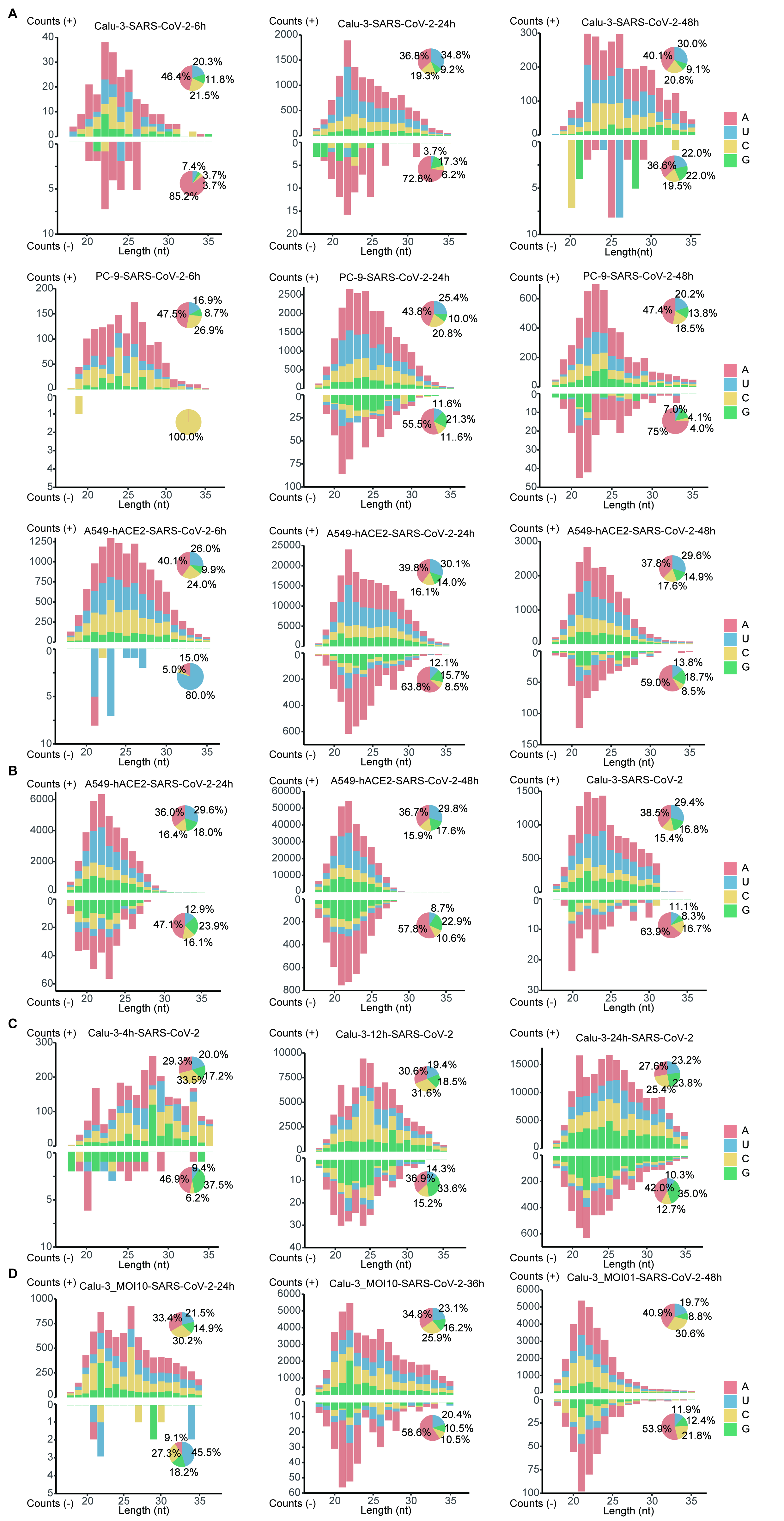

Supplement: Supplementary Figure 1 — Description of sRNA libraries in control and SARS-CoV-2-infected Vero cells. (A) Correlation of miRNA reads between two biological replicates in control and SARS-CoV-2-infected (6 hpi/12 hpi) Vero cells. (B) PCA plot of miRNA-seq libraries of control and virus-infected (6 hpi/12 hpi) Vero cells. [file DataSheet_1.zip › Data Sheet 1/Figure S4.tif]

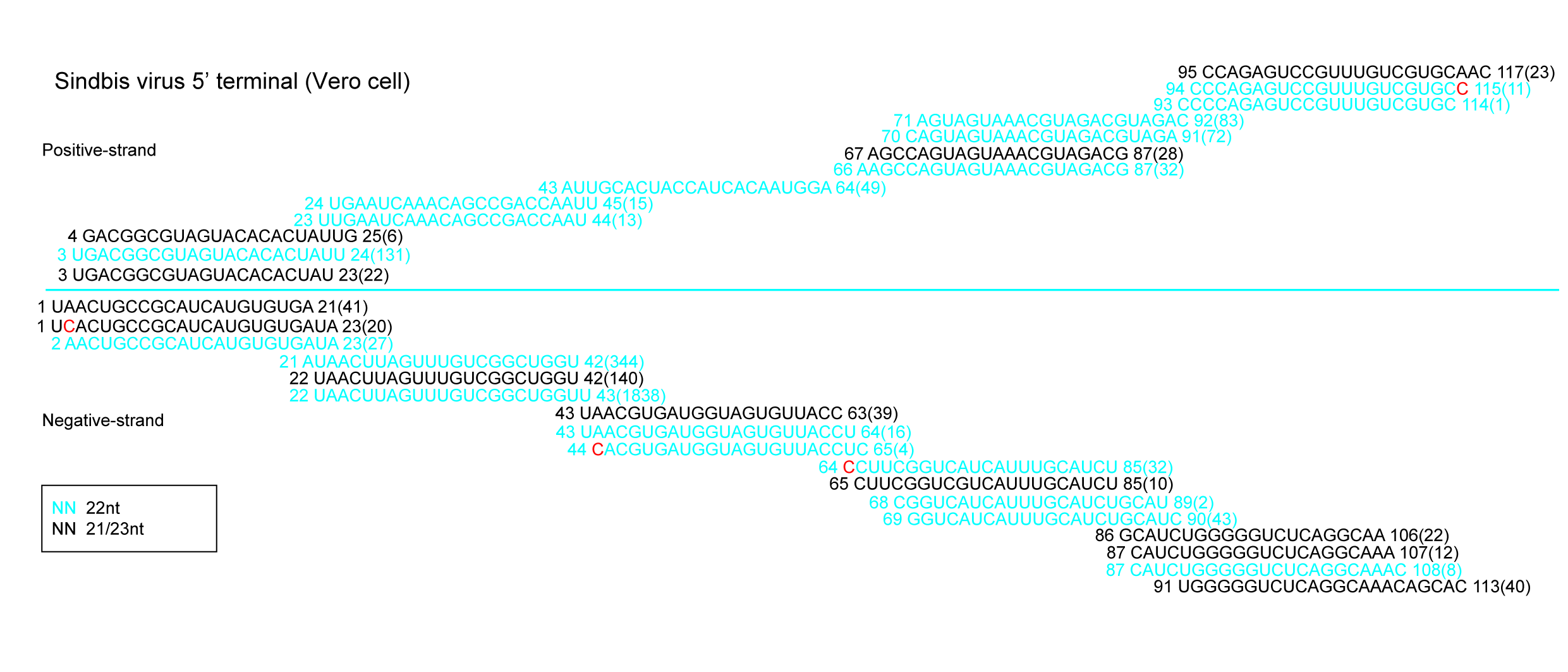

Supplement: Supplementary Figure 1 — Description of sRNA libraries in control and SARS-CoV-2-infected Vero cells. (A) Correlation of miRNA reads between two biological replicates in control and SARS-CoV-2-infected (6 hpi/12 hpi) Vero cells. (B) PCA plot of miRNA-seq libraries of control and virus-infected (6 hpi/12 hpi) Vero cells. [file DataSheet_1.zip › Data Sheet 1/Figure S5.tif]

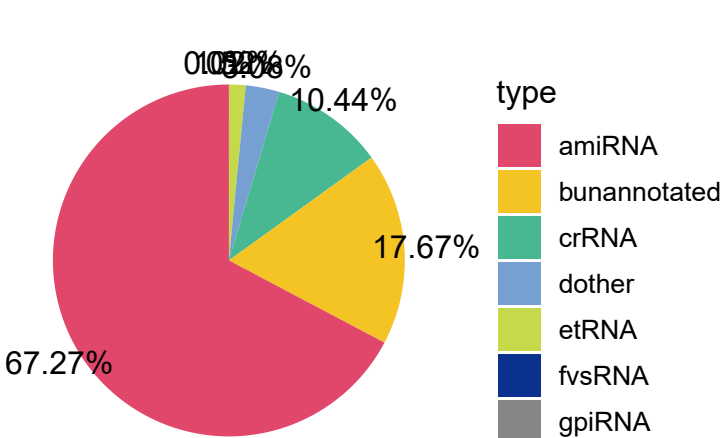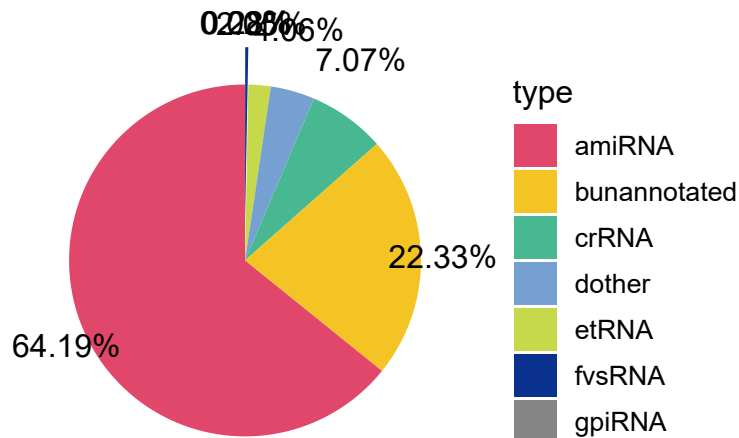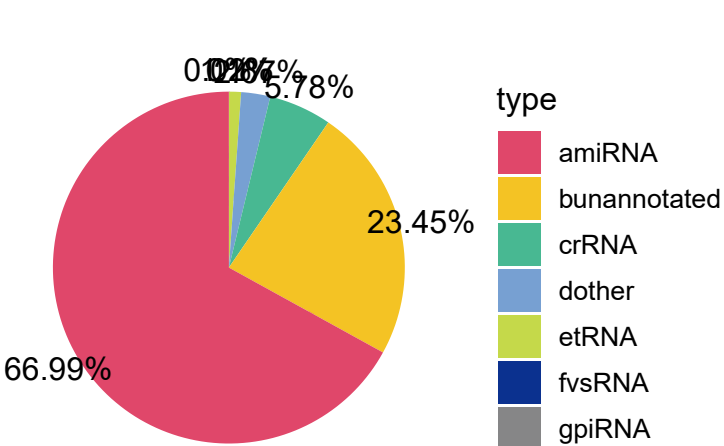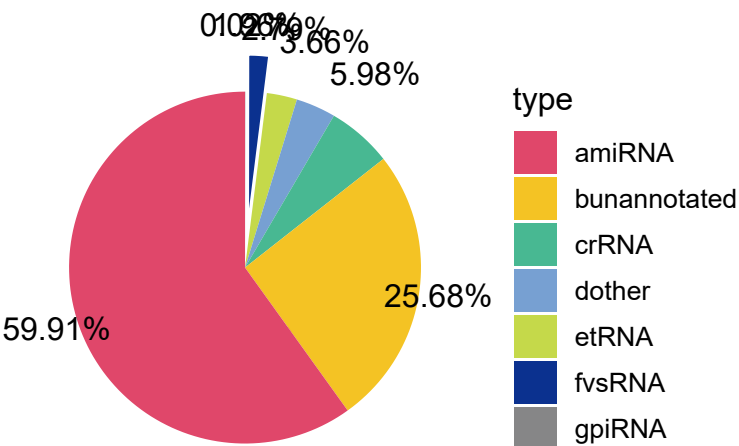

Supplement: Supplementary Figure 1 — Description of sRNA libraries in control and SARS-CoV-2-infected Vero cells. (A) Correlation of miRNA reads between two biological replicates in control and SARS-CoV-2-infected (6 hpi/12 hpi) Vero cells. (B) PCA plot of miRNA-seq libraries of control and virus-infected (6 hpi/12 hpi) Vero cells. [file DataSheet_1.zip › Data Sheet 1/SARS-CoV-2 source code & data/SARS-CoV-2 source code/fig1/1B/output/pie-new.pdf]

Vero-Control-6h

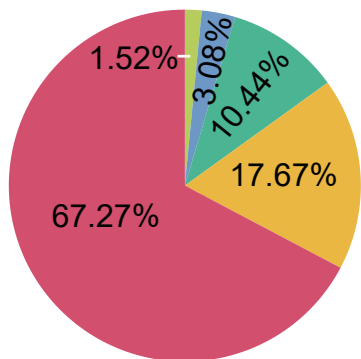

Vero-SARS-CoV-2-6h

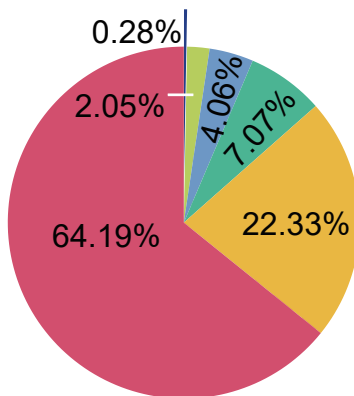

Vero-Control-12h

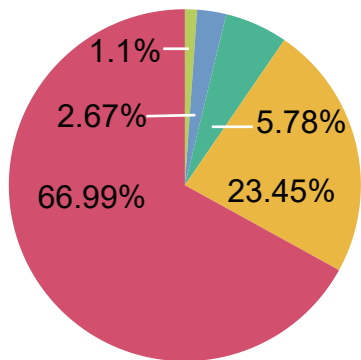

Vero-SARS-CoV-2-12h

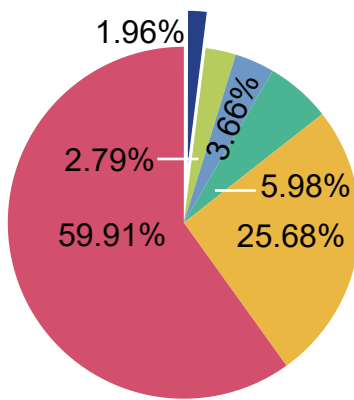

type

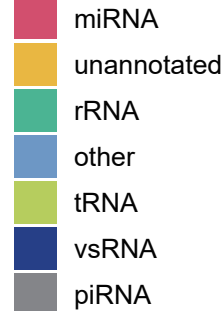

Supplement: Supplementary Figure 1 — Description of sRNA libraries in control and SARS-CoV-2-infected Vero cells. (A) Correlation of miRNA reads between two biological replicates in control and SARS-CoV-2-infected (6 hpi/12 hpi) Vero cells. (B) PCA plot of miRNA-seq libraries of control and virus-infected (6 hpi/12 hpi) Vero cells. [file DataSheet_1.zip › Data Sheet 1/SARS-CoV-2 source code & data/SARS-CoV-2 source code/fig1/1B/output/pie-new_AI.pdf]

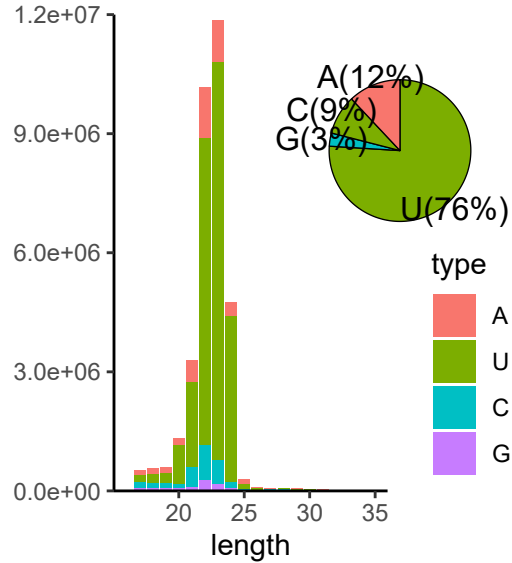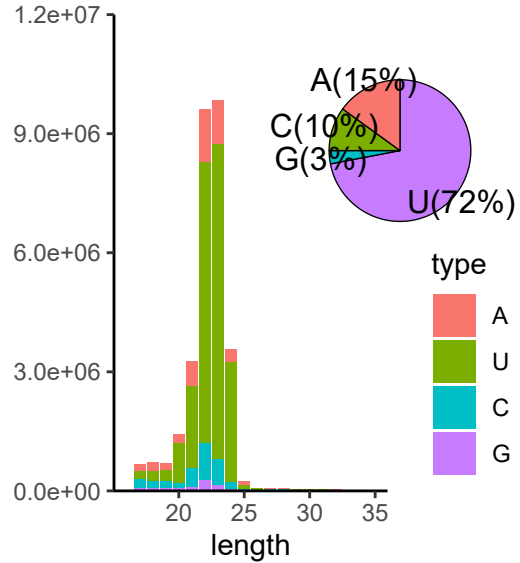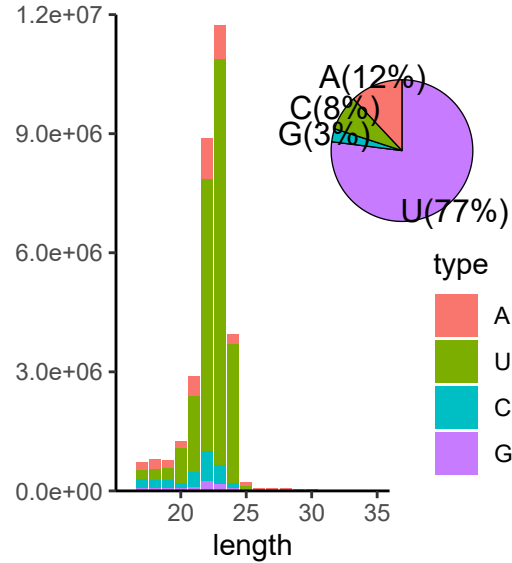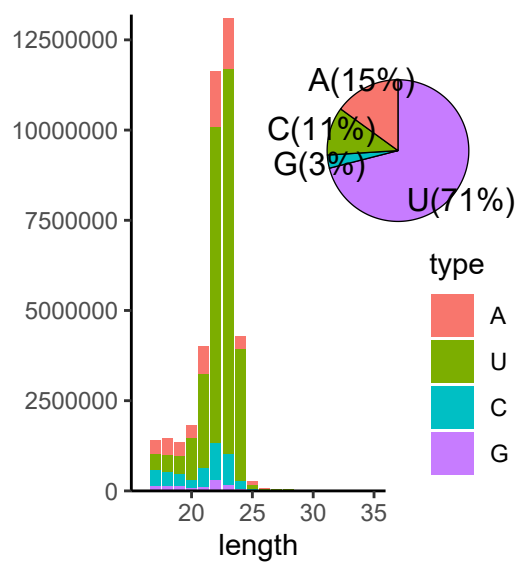

Supplement: Supplementary Figure 1 — Description of sRNA libraries in control and SARS-CoV-2-infected Vero cells. (A) Correlation of miRNA reads between two biological replicates in control and SARS-CoV-2-infected (6 hpi/12 hpi) Vero cells. (B) PCA plot of miRNA-seq libraries of control and virus-infected (6 hpi/12 hpi) Vero cells. [file DataSheet_1.zip › Data Sheet 1/SARS-CoV-2 source code & data/SARS-CoV-2 source code/fig1/1C/output/nostr.pdf]

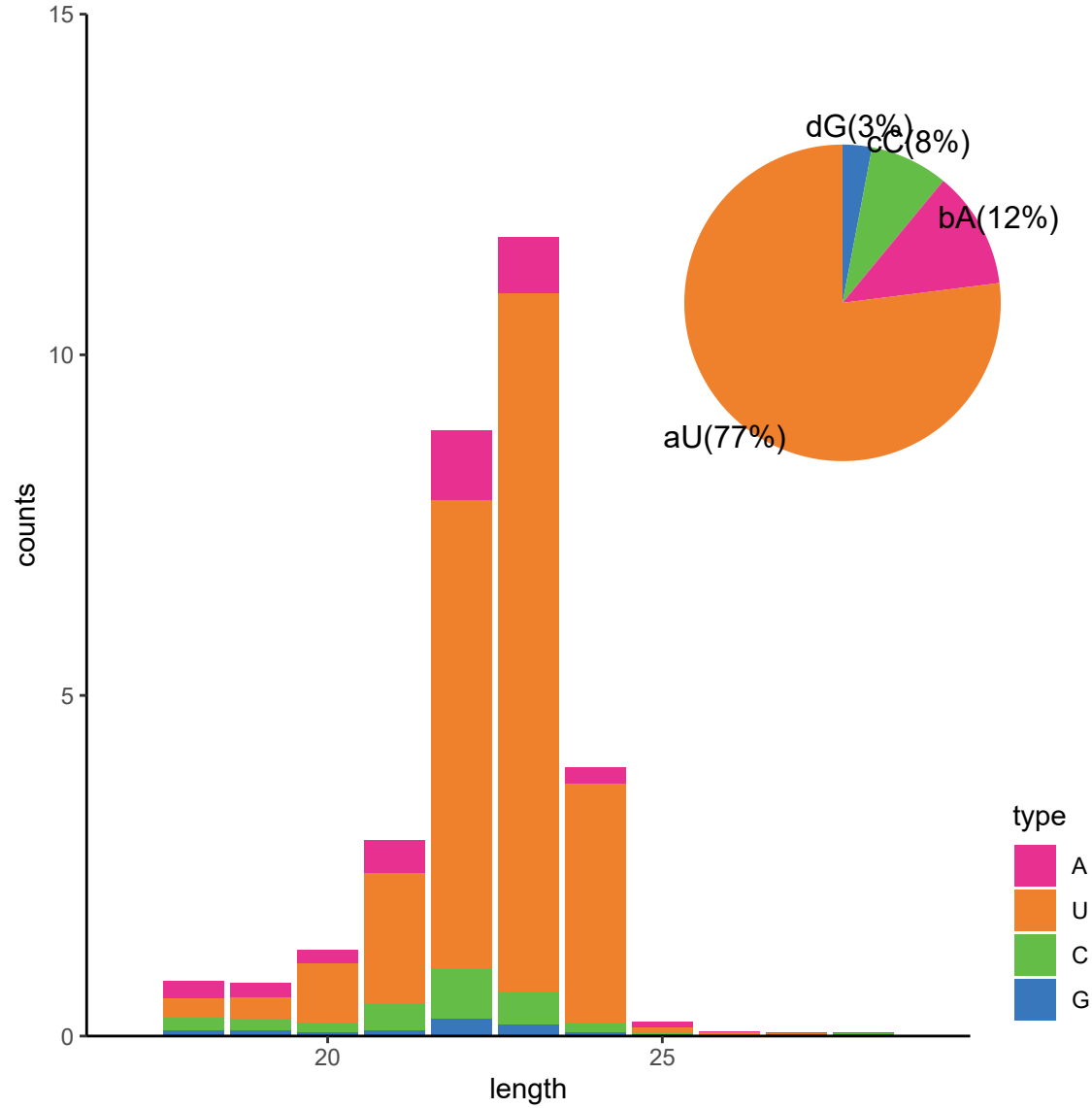

Supplement: Supplementary Figure 1 — Description of sRNA libraries in control and SARS-CoV-2-infected Vero cells. (A) Correlation of miRNA reads between two biological replicates in control and SARS-CoV-2-infected (6 hpi/12 hpi) Vero cells. (B) PCA plot of miRNA-seq libraries of control and virus-infected (6 hpi/12 hpi) Vero cells. [file DataSheet_1.zip › Data Sheet 1/SARS-CoV-2 source code & data/SARS-CoV-2 source code/fig1/1C/output/vero_conrol12h.pdf]

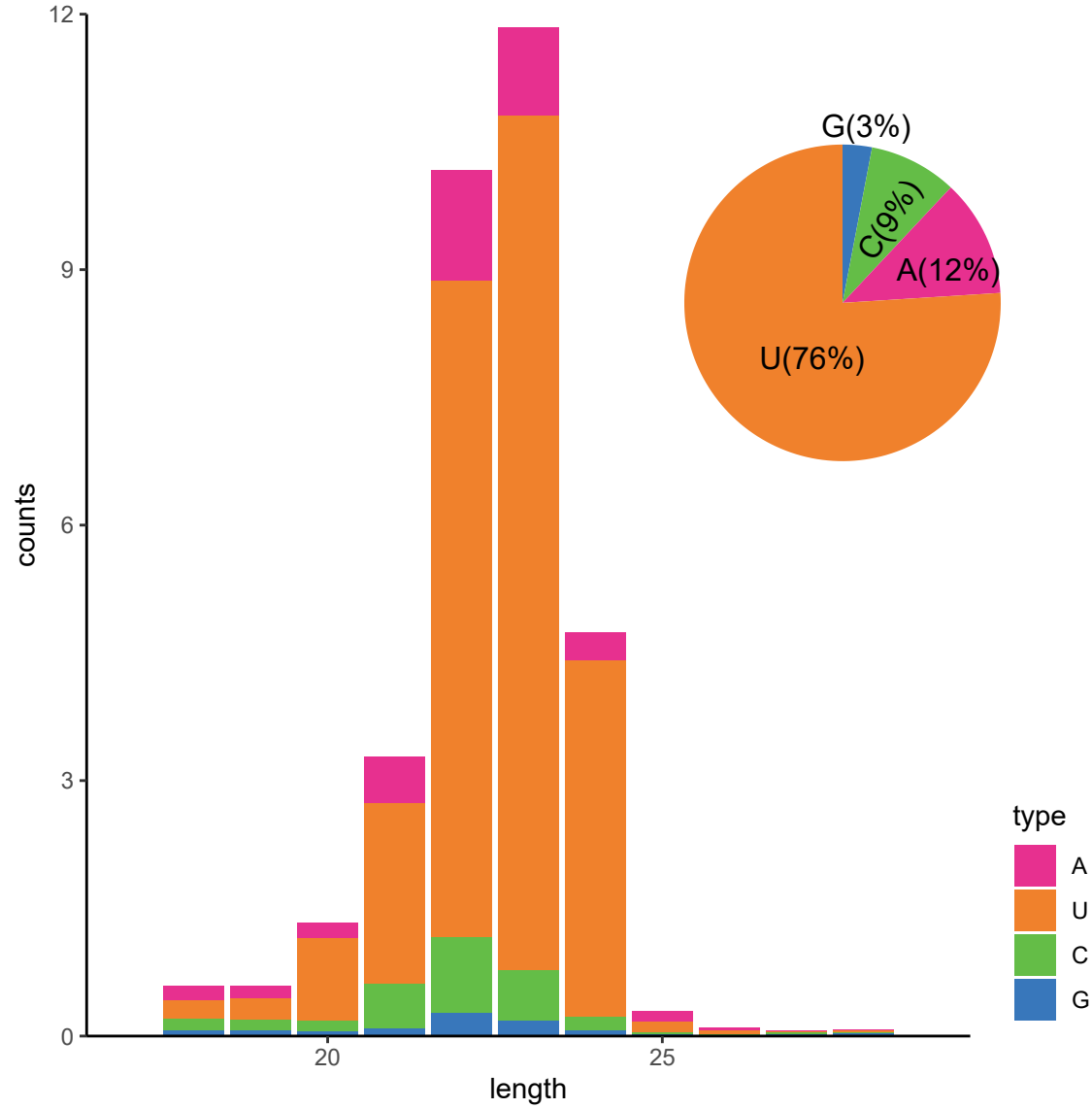

Supplement: Supplementary Figure 1 — Description of sRNA libraries in control and SARS-CoV-2-infected Vero cells. (A) Correlation of miRNA reads between two biological replicates in control and SARS-CoV-2-infected (6 hpi/12 hpi) Vero cells. (B) PCA plot of miRNA-seq libraries of control and virus-infected (6 hpi/12 hpi) Vero cells. [file DataSheet_1.zip › Data Sheet 1/SARS-CoV-2 source code & data/SARS-CoV-2 source code/fig1/1C/output/vero_conrol6h.pdf]

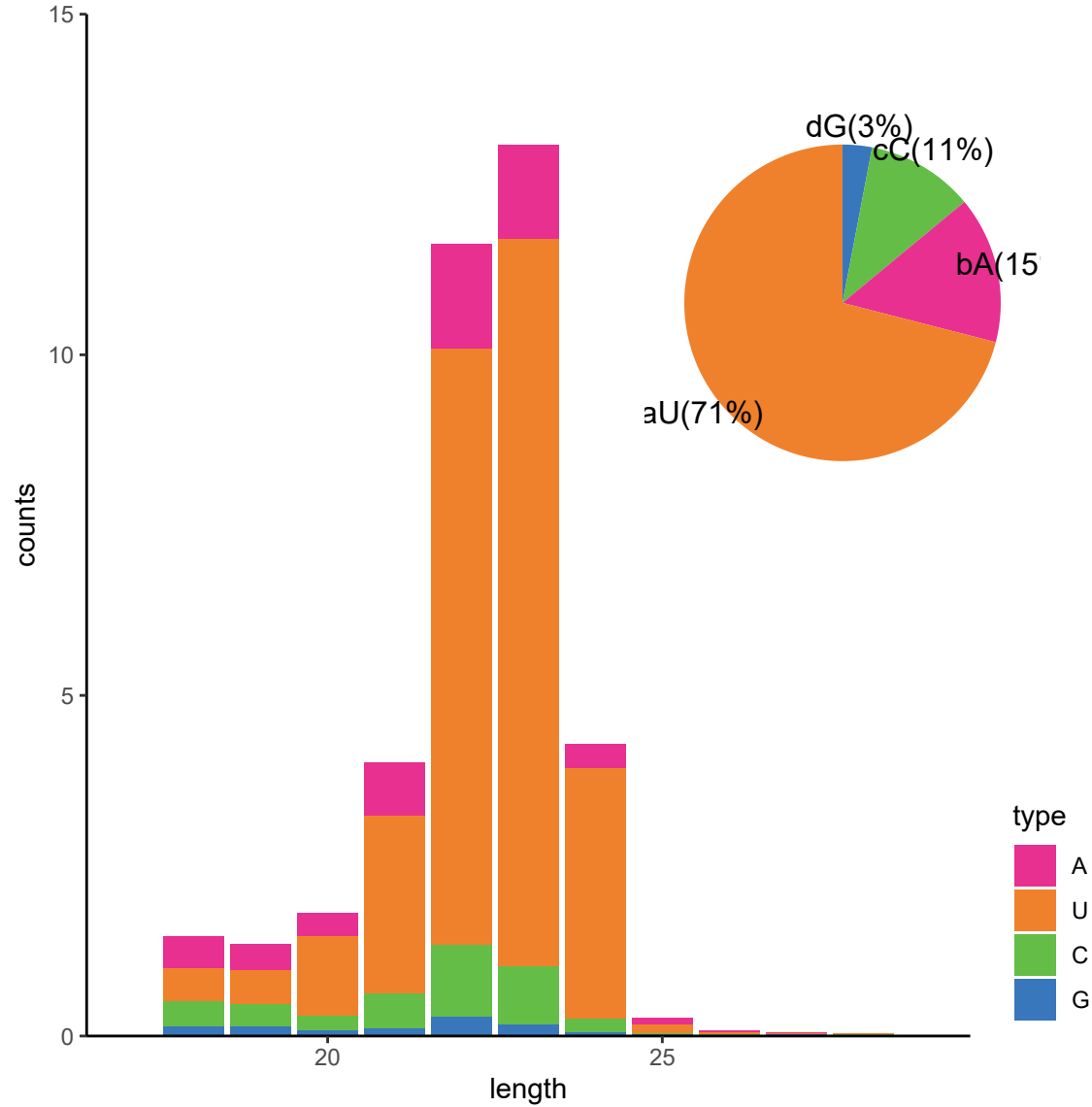

Supplement: Supplementary Figure 1 — Description of sRNA libraries in control and SARS-CoV-2-infected Vero cells. (A) Correlation of miRNA reads between two biological replicates in control and SARS-CoV-2-infected (6 hpi/12 hpi) Vero cells. (B) PCA plot of miRNA-seq libraries of control and virus-infected (6 hpi/12 hpi) Vero cells. [file DataSheet_1.zip › Data Sheet 1/SARS-CoV-2 source code & data/SARS-CoV-2 source code/fig1/1C/output/vero_SARS12h.pdf]

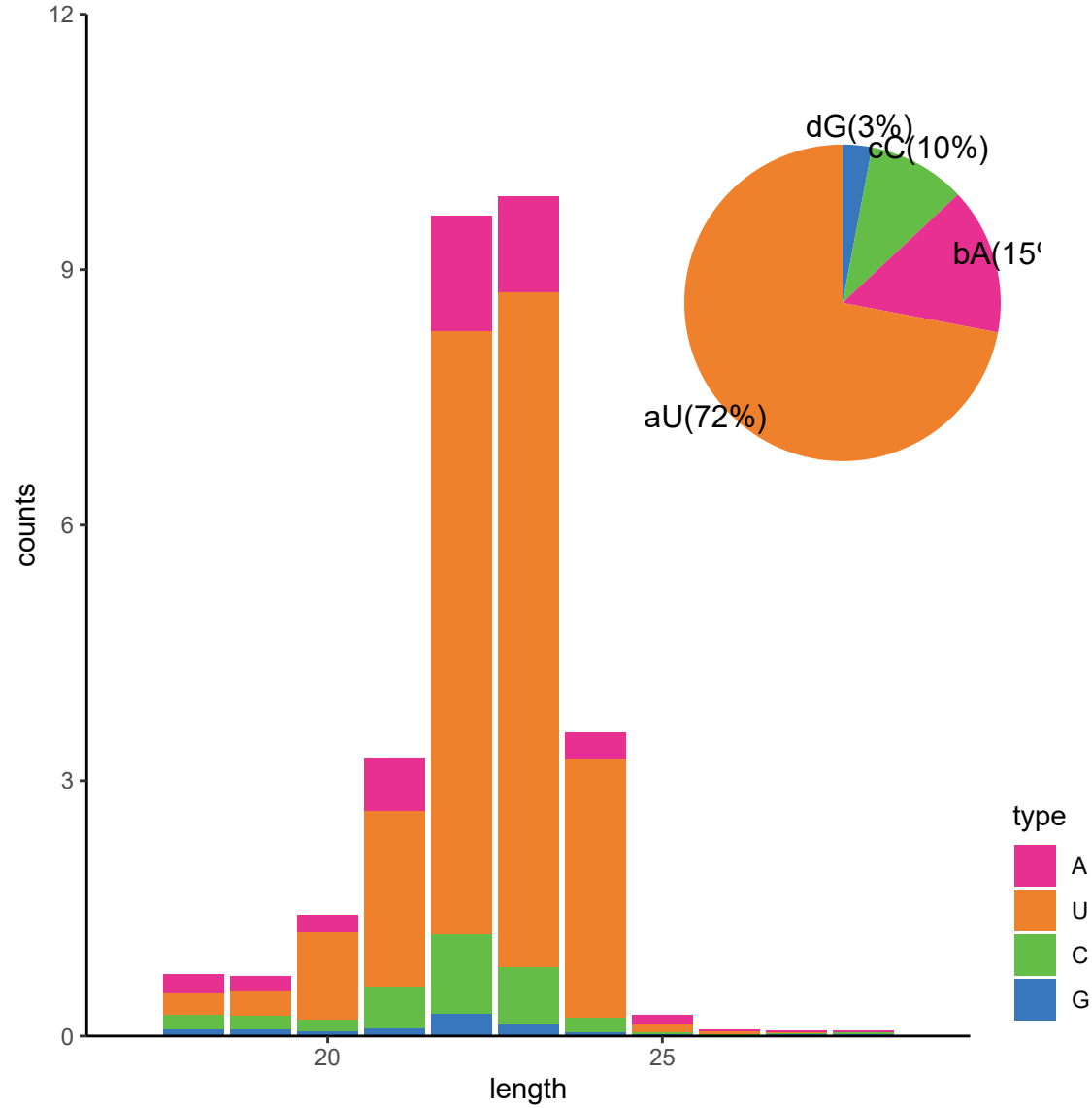

Supplement: Supplementary Figure 1 — Description of sRNA libraries in control and SARS-CoV-2-infected Vero cells. (A) Correlation of miRNA reads between two biological replicates in control and SARS-CoV-2-infected (6 hpi/12 hpi) Vero cells. (B) PCA plot of miRNA-seq libraries of control and virus-infected (6 hpi/12 hpi) Vero cells. [file DataSheet_1.zip › Data Sheet 1/SARS-CoV-2 source code & data/SARS-CoV-2 source code/fig1/1C/output/vero_SARS6h.pdf]

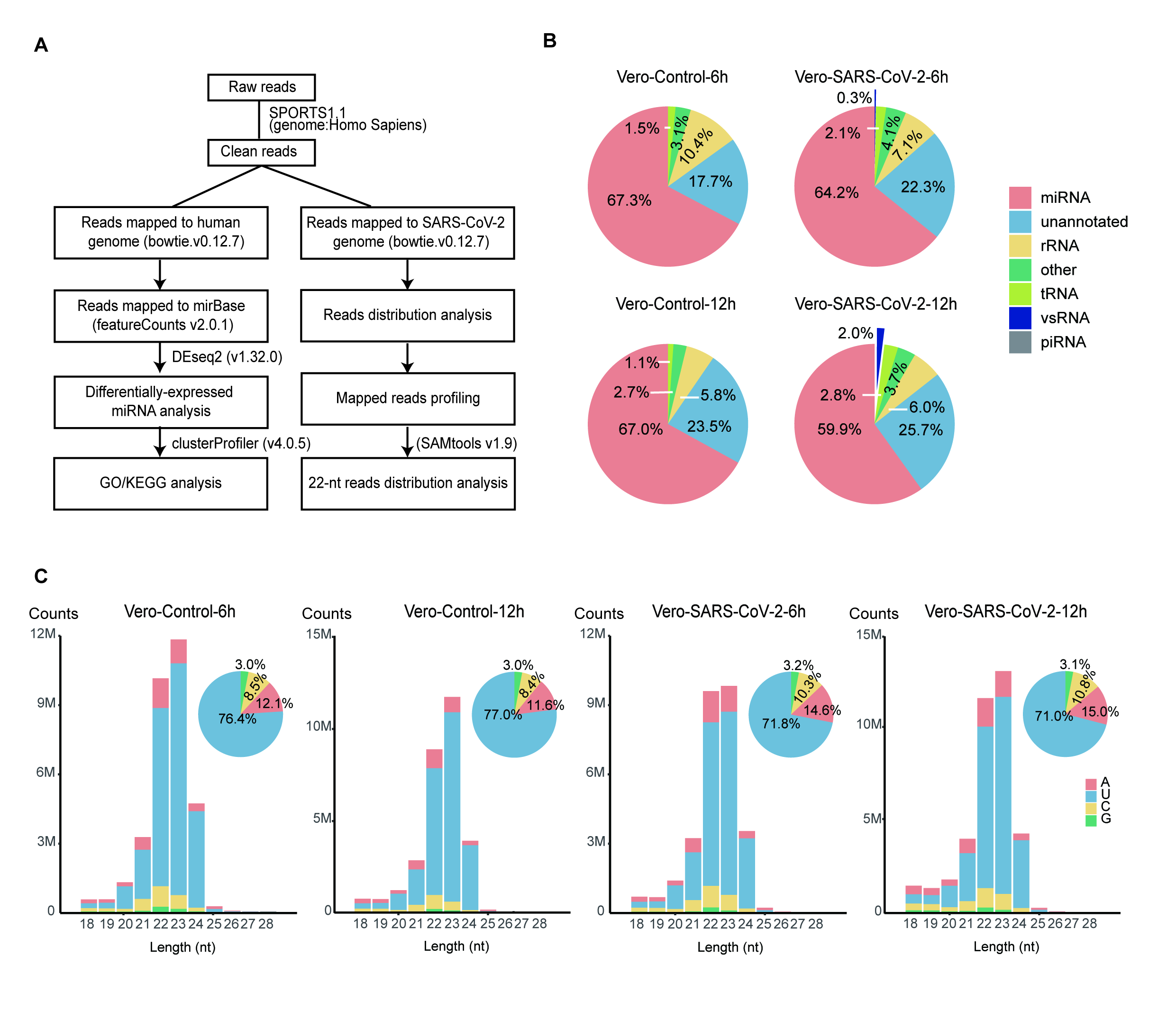

Supplement: Supplementary Figure 1 — Description of sRNA libraries in control and SARS-CoV-2-infected Vero cells. (A) Correlation of miRNA reads between two biological replicates in control and SARS-CoV-2-infected (6 hpi/12 hpi) Vero cells. (B) PCA plot of miRNA-seq libraries of control and virus-infected (6 hpi/12 hpi) Vero cells. [file DataSheet_1.zip › Data Sheet 1/SARS-CoV-2 source code & data/SARS-CoV-2 source code/fig1/Figure1.tif]

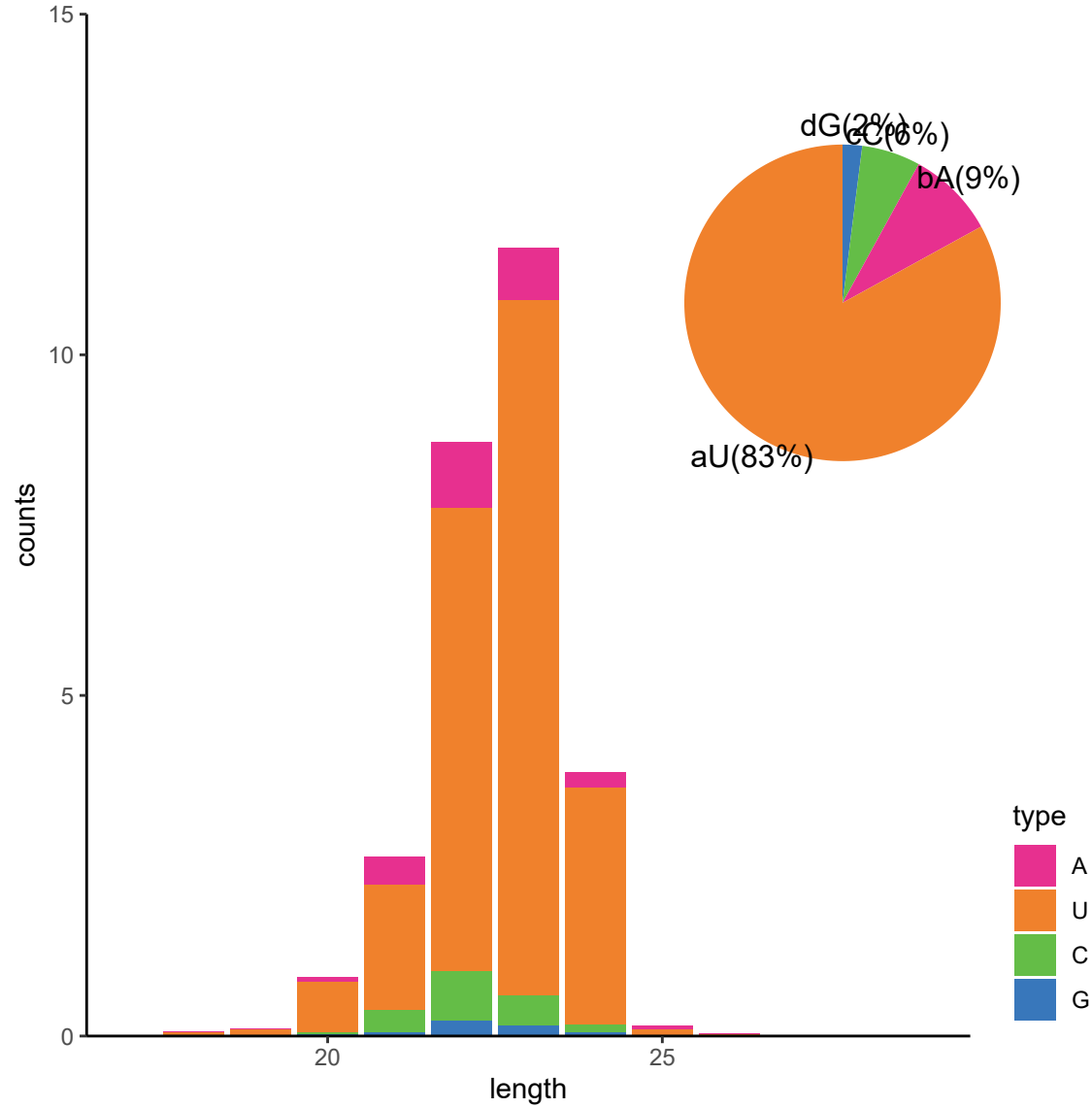

Supplement: Supplementary Figure 1 — Description of sRNA libraries in control and SARS-CoV-2-infected Vero cells. (A) Correlation of miRNA reads between two biological replicates in control and SARS-CoV-2-infected (6 hpi/12 hpi) Vero cells. (B) PCA plot of miRNA-seq libraries of control and virus-infected (6 hpi/12 hpi) Vero cells. [file DataSheet_1.zip › Data Sheet 1/SARS-CoV-2 source code & data/SARS-CoV-2 source code/fig2/2A/output/Vero_control-12h-miRNA.pdf]

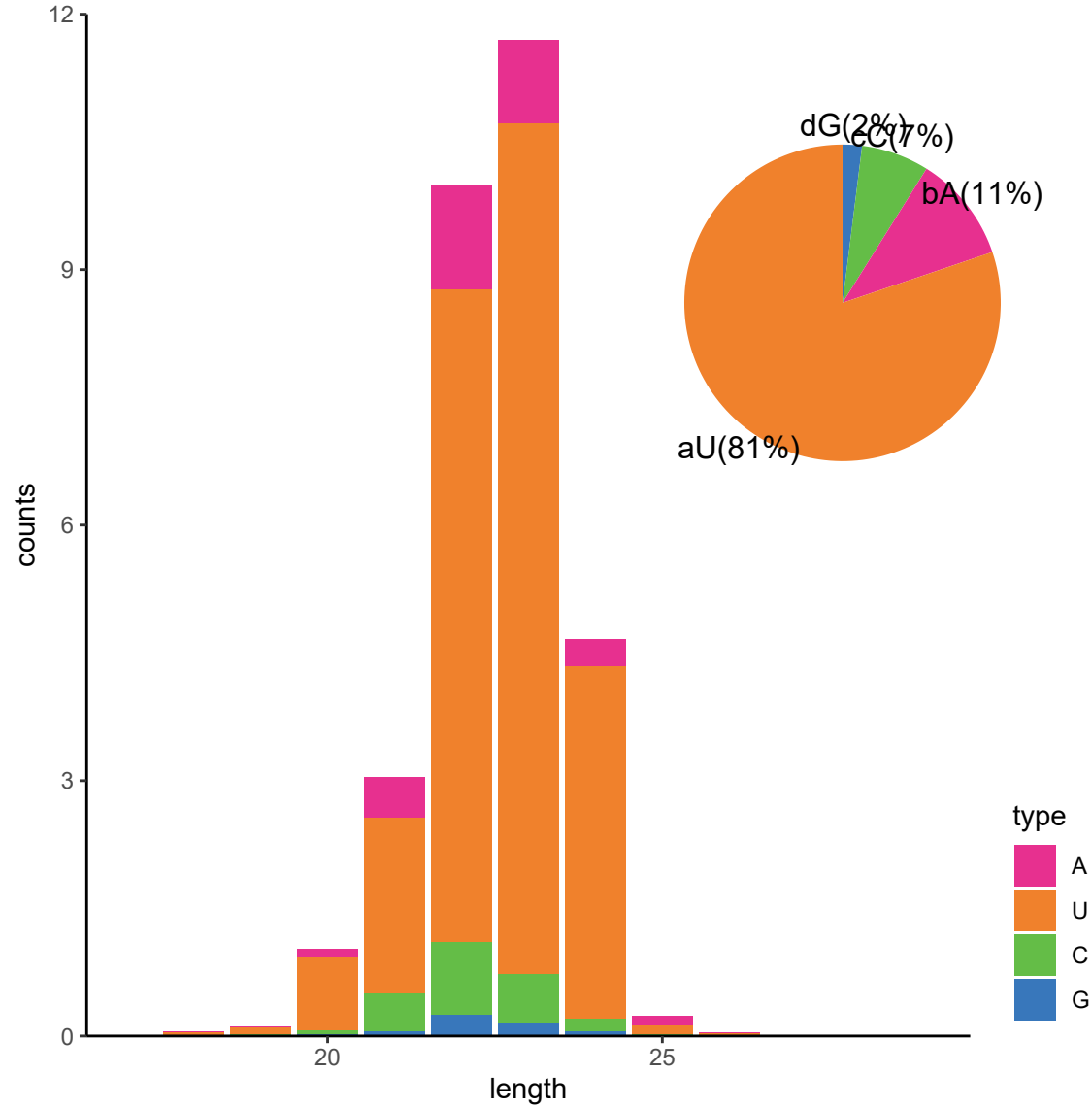

Supplement: Supplementary Figure 1 — Description of sRNA libraries in control and SARS-CoV-2-infected Vero cells. (A) Correlation of miRNA reads between two biological replicates in control and SARS-CoV-2-infected (6 hpi/12 hpi) Vero cells. (B) PCA plot of miRNA-seq libraries of control and virus-infected (6 hpi/12 hpi) Vero cells. [file DataSheet_1.zip › Data Sheet 1/SARS-CoV-2 source code & data/SARS-CoV-2 source code/fig2/2A/output/Vero_control-6h-miRNA.pdf]

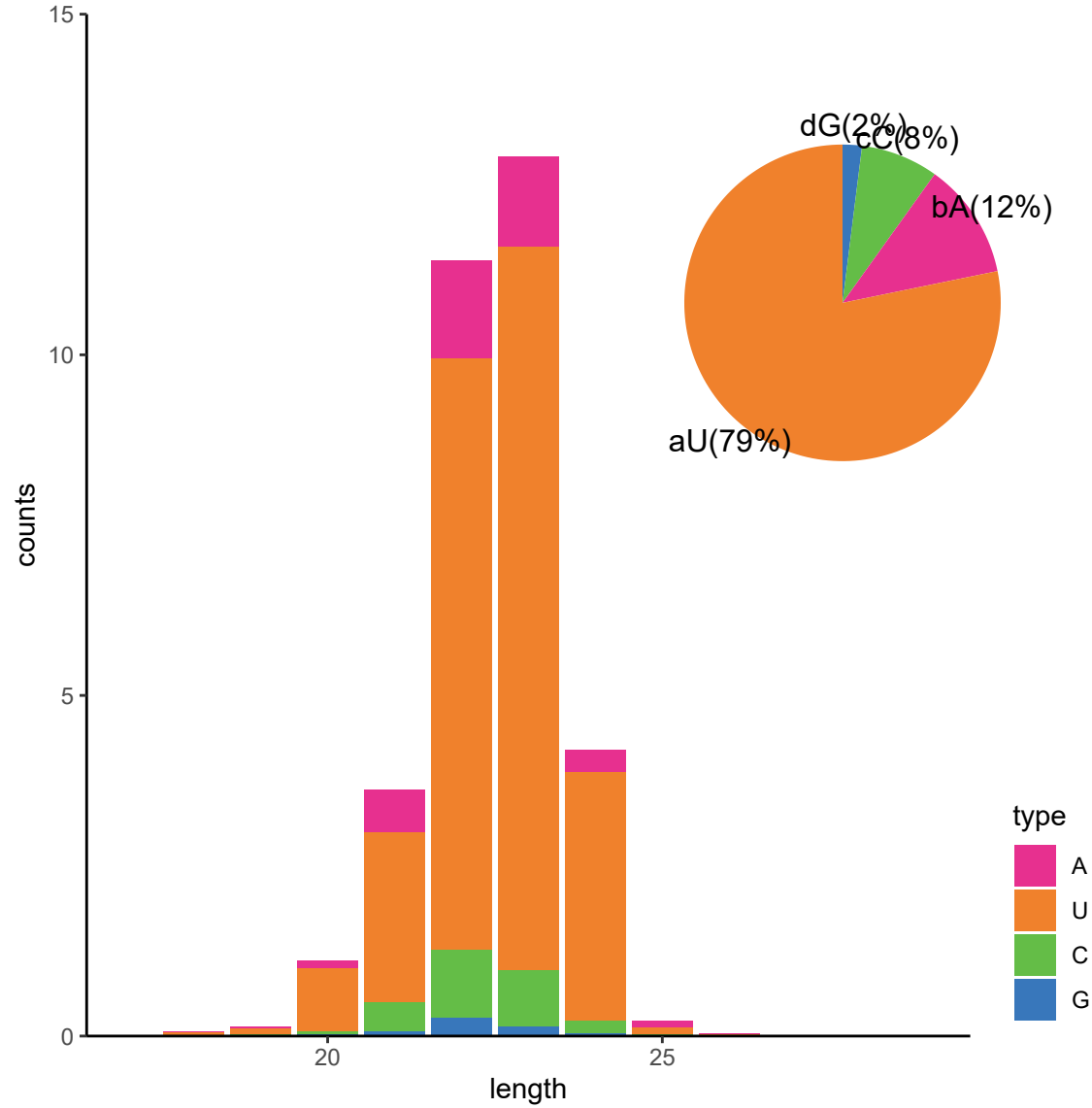

Supplement: Supplementary Figure 1 — Description of sRNA libraries in control and SARS-CoV-2-infected Vero cells. (A) Correlation of miRNA reads between two biological replicates in control and SARS-CoV-2-infected (6 hpi/12 hpi) Vero cells. (B) PCA plot of miRNA-seq libraries of control and virus-infected (6 hpi/12 hpi) Vero cells. [file DataSheet_1.zip › Data Sheet 1/SARS-CoV-2 source code & data/SARS-CoV-2 source code/fig2/2A/output/Vero_SARS-CoV-2-12h-miRNA.pdf]

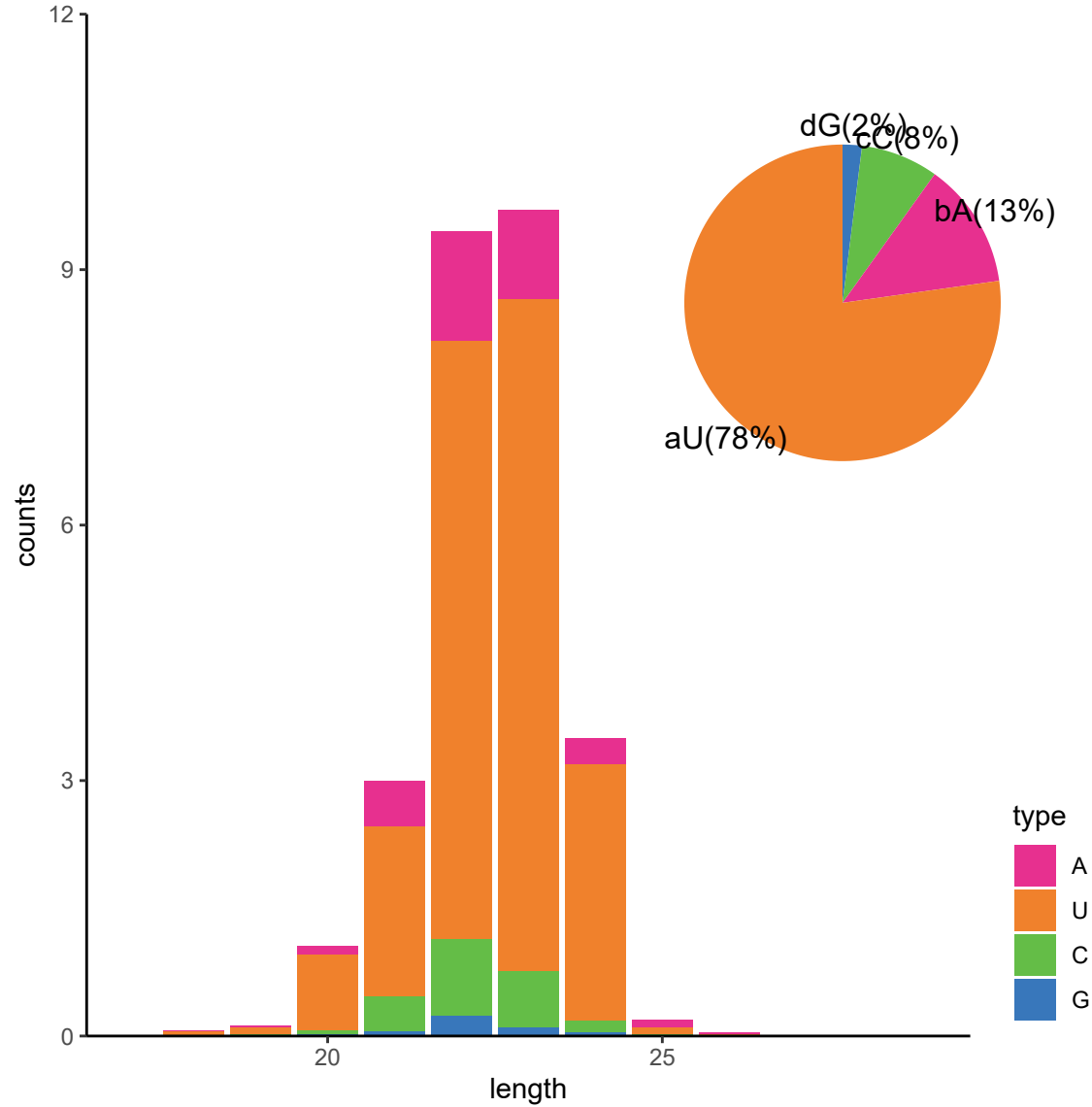

Supplement: Supplementary Figure 1 — Description of sRNA libraries in control and SARS-CoV-2-infected Vero cells. (A) Correlation of miRNA reads between two biological replicates in control and SARS-CoV-2-infected (6 hpi/12 hpi) Vero cells. (B) PCA plot of miRNA-seq libraries of control and virus-infected (6 hpi/12 hpi) Vero cells. [file DataSheet_1.zip › Data Sheet 1/SARS-CoV-2 source code & data/SARS-CoV-2 source code/fig2/2A/output/Vero_SARS-CoV-2-6h-miRNA.pdf]

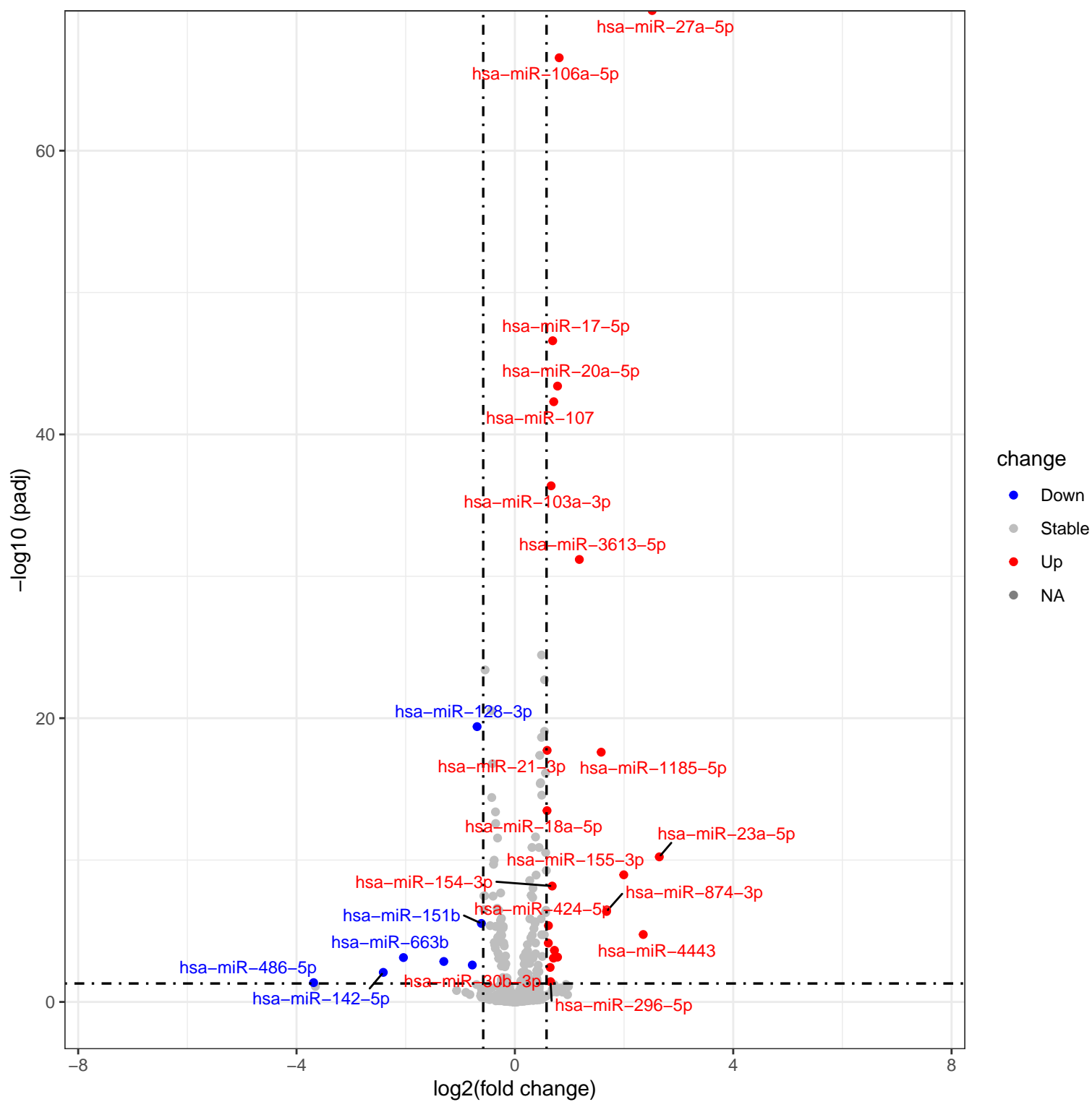

Supplement: Supplementary Figure 1 — Description of sRNA libraries in control and SARS-CoV-2-infected Vero cells. (A) Correlation of miRNA reads between two biological replicates in control and SARS-CoV-2-infected (6 hpi/12 hpi) Vero cells. (B) PCA plot of miRNA-seq libraries of control and virus-infected (6 hpi/12 hpi) Vero cells. [file DataSheet_1.zip › Data Sheet 1/SARS-CoV-2 source code & data/SARS-CoV-2 source code/fig2/2B/output/SARS-Cov-2 vs Control-12h-volcano.pdf]

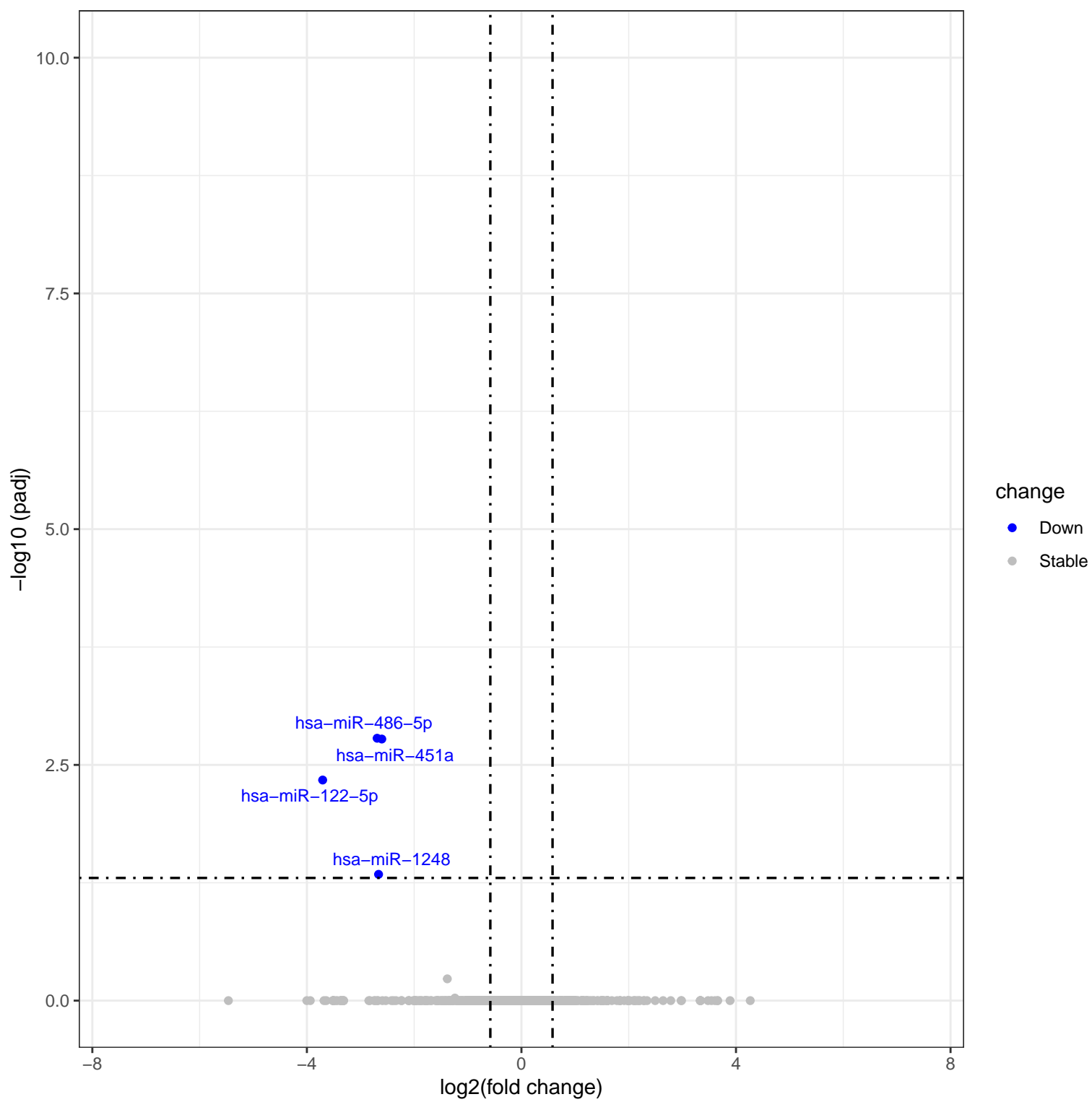

Supplement: Supplementary Figure 1 — Description of sRNA libraries in control and SARS-CoV-2-infected Vero cells. (A) Correlation of miRNA reads between two biological replicates in control and SARS-CoV-2-infected (6 hpi/12 hpi) Vero cells. (B) PCA plot of miRNA-seq libraries of control and virus-infected (6 hpi/12 hpi) Vero cells. [file DataSheet_1.zip › Data Sheet 1/SARS-CoV-2 source code & data/SARS-CoV-2 source code/fig2/2B/output/SARS-Cov-2 vs Control-6h-volcano.pdf]

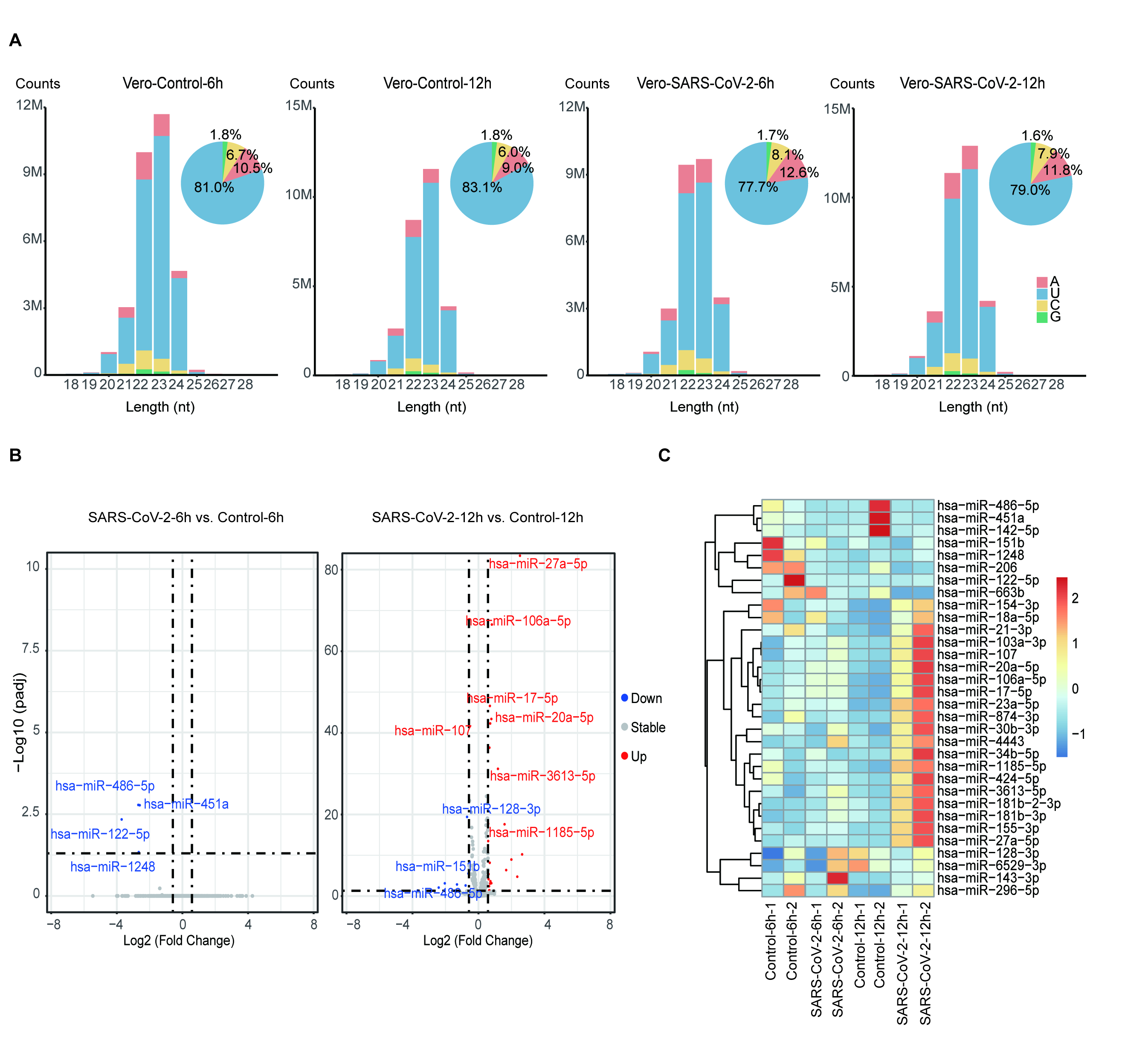

Supplement: Supplementary Figure 1 — Description of sRNA libraries in control and SARS-CoV-2-infected Vero cells. (A) Correlation of miRNA reads between two biological replicates in control and SARS-CoV-2-infected (6 hpi/12 hpi) Vero cells. (B) PCA plot of miRNA-seq libraries of control and virus-infected (6 hpi/12 hpi) Vero cells. [file DataSheet_1.zip › Data Sheet 1/SARS-CoV-2 source code & data/SARS-CoV-2 source code/fig2/Figure2.tif]

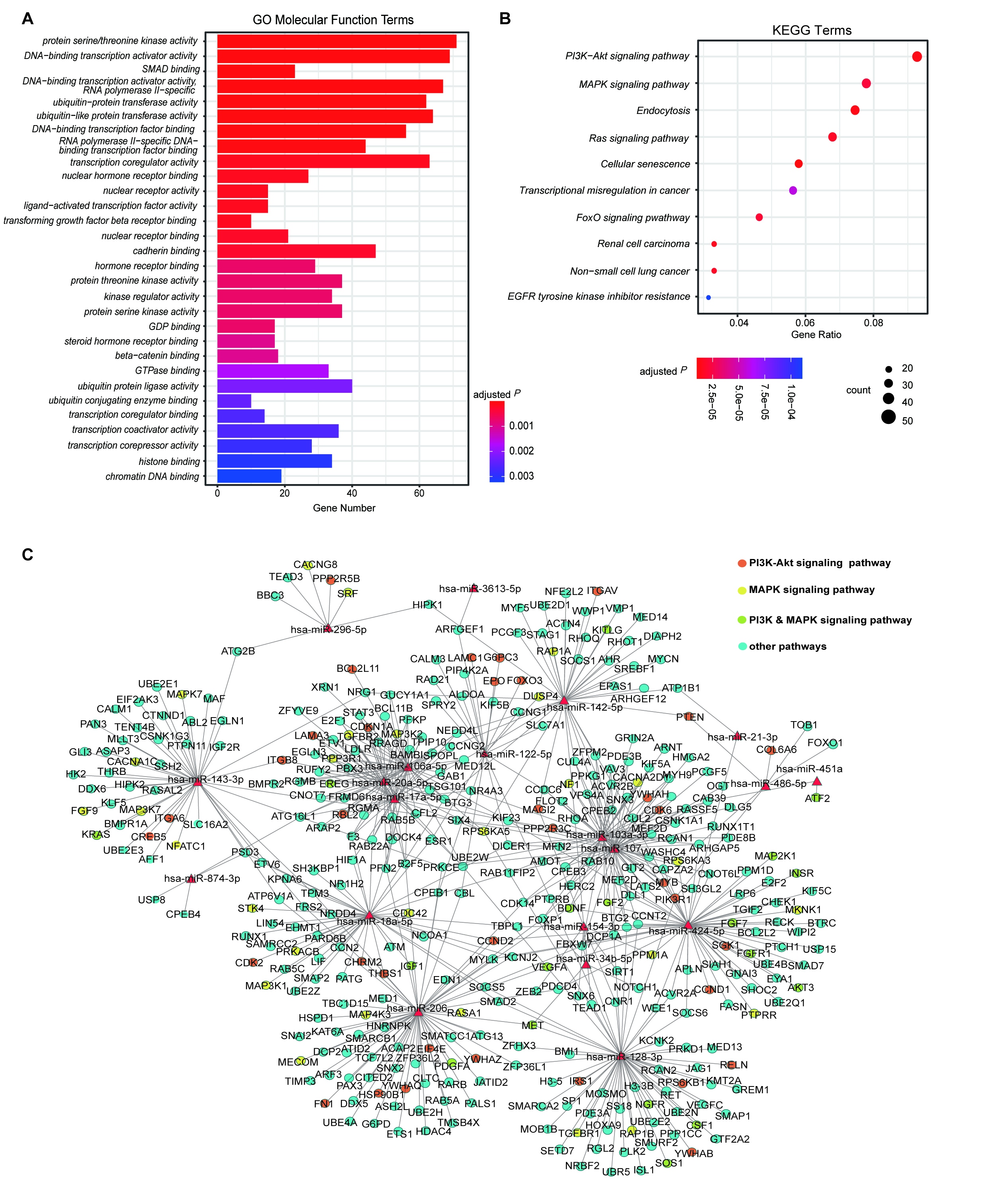

Supplement: Supplementary Figure 1 — Description of sRNA libraries in control and SARS-CoV-2-infected Vero cells. (A) Correlation of miRNA reads between two biological replicates in control and SARS-CoV-2-infected (6 hpi/12 hpi) Vero cells. (B) PCA plot of miRNA-seq libraries of control and virus-infected (6 hpi/12 hpi) Vero cells. [file DataSheet_1.zip › Data Sheet 1/SARS-CoV-2 source code & data/SARS-CoV-2 source code/fig3/Figure3.tif]

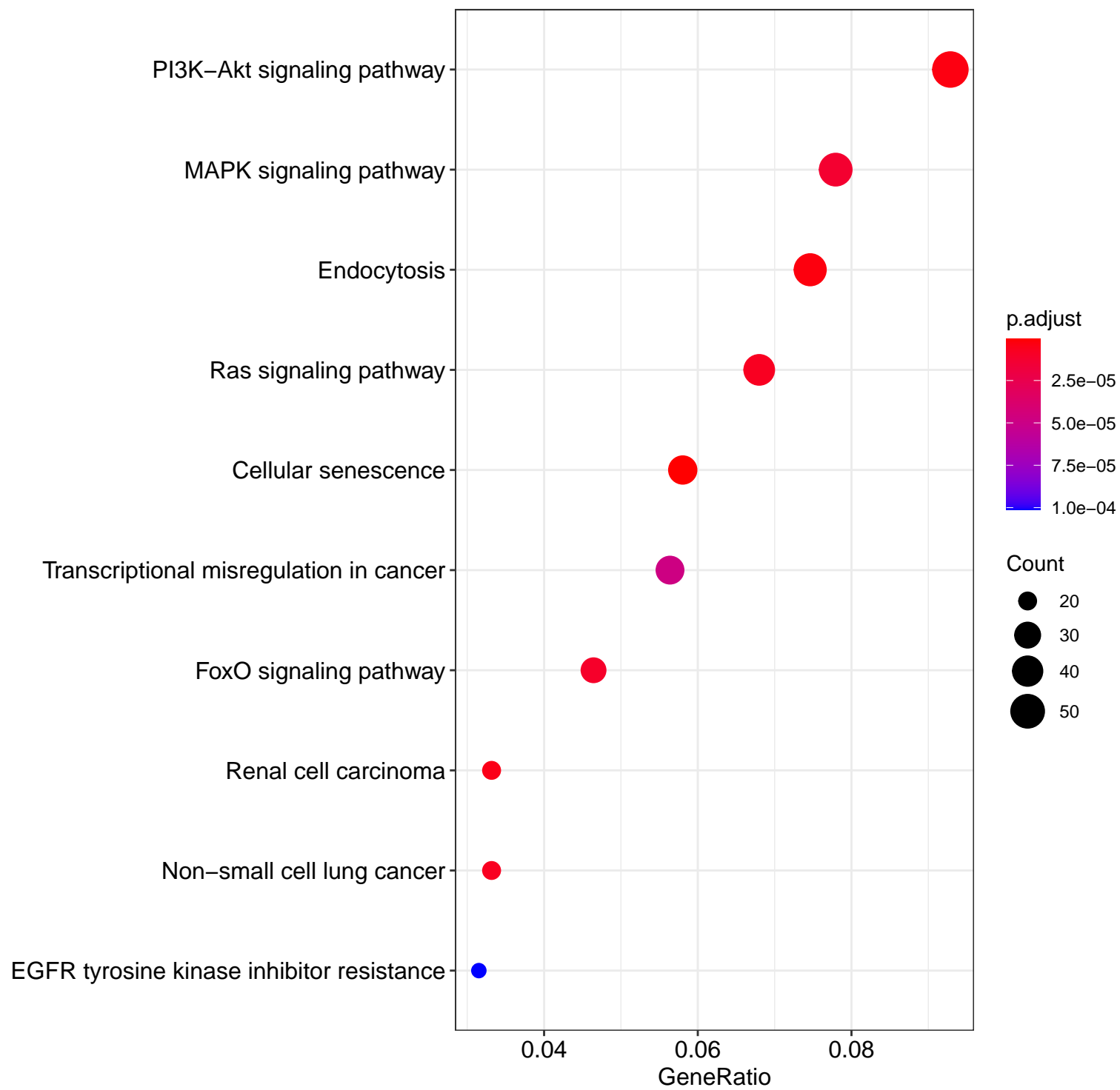

Supplement: Supplementary Figure 1 — Description of sRNA libraries in control and SARS-CoV-2-infected Vero cells. (A) Correlation of miRNA reads between two biological replicates in control and SARS-CoV-2-infected (6 hpi/12 hpi) Vero cells. (B) PCA plot of miRNA-seq libraries of control and virus-infected (6 hpi/12 hpi) Vero cells. [file DataSheet_1.zip › Data Sheet 1/SARS-CoV-2 source code & data/SARS-CoV-2 source code/fig3/output/KEGG.pdf]

# GO\_MF Terms

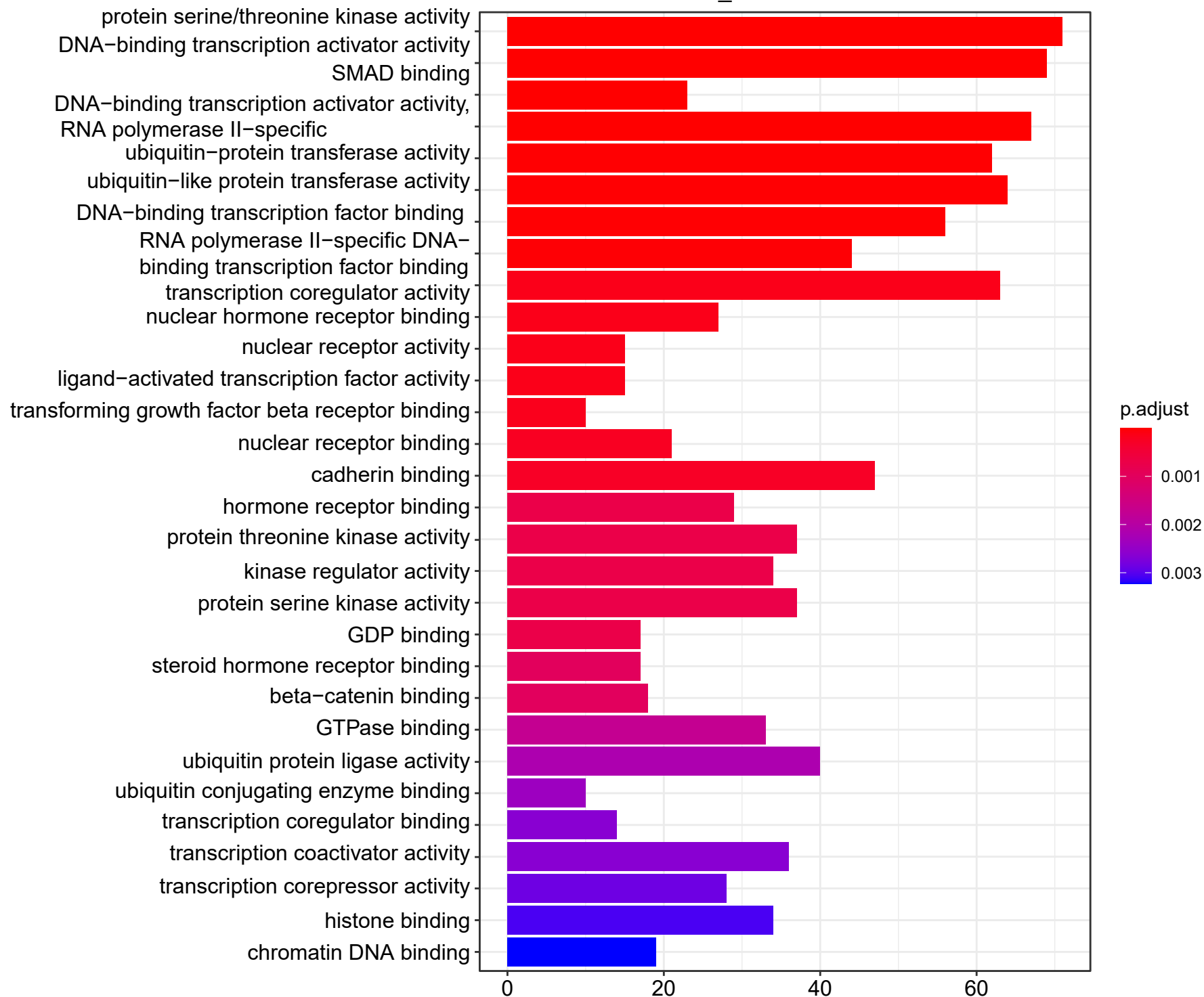

Supplement: Supplementary Figure 1 — Description of sRNA libraries in control and SARS-CoV-2-infected Vero cells. (A) Correlation of miRNA reads between two biological replicates in control and SARS-CoV-2-infected (6 hpi/12 hpi) Vero cells. (B) PCA plot of miRNA-seq libraries of control and virus-infected (6 hpi/12 hpi) Vero cells. [file DataSheet_1.zip › Data Sheet 1/SARS-CoV-2 source code & data/SARS-CoV-2 source code/fig3/output/MF.pdf]

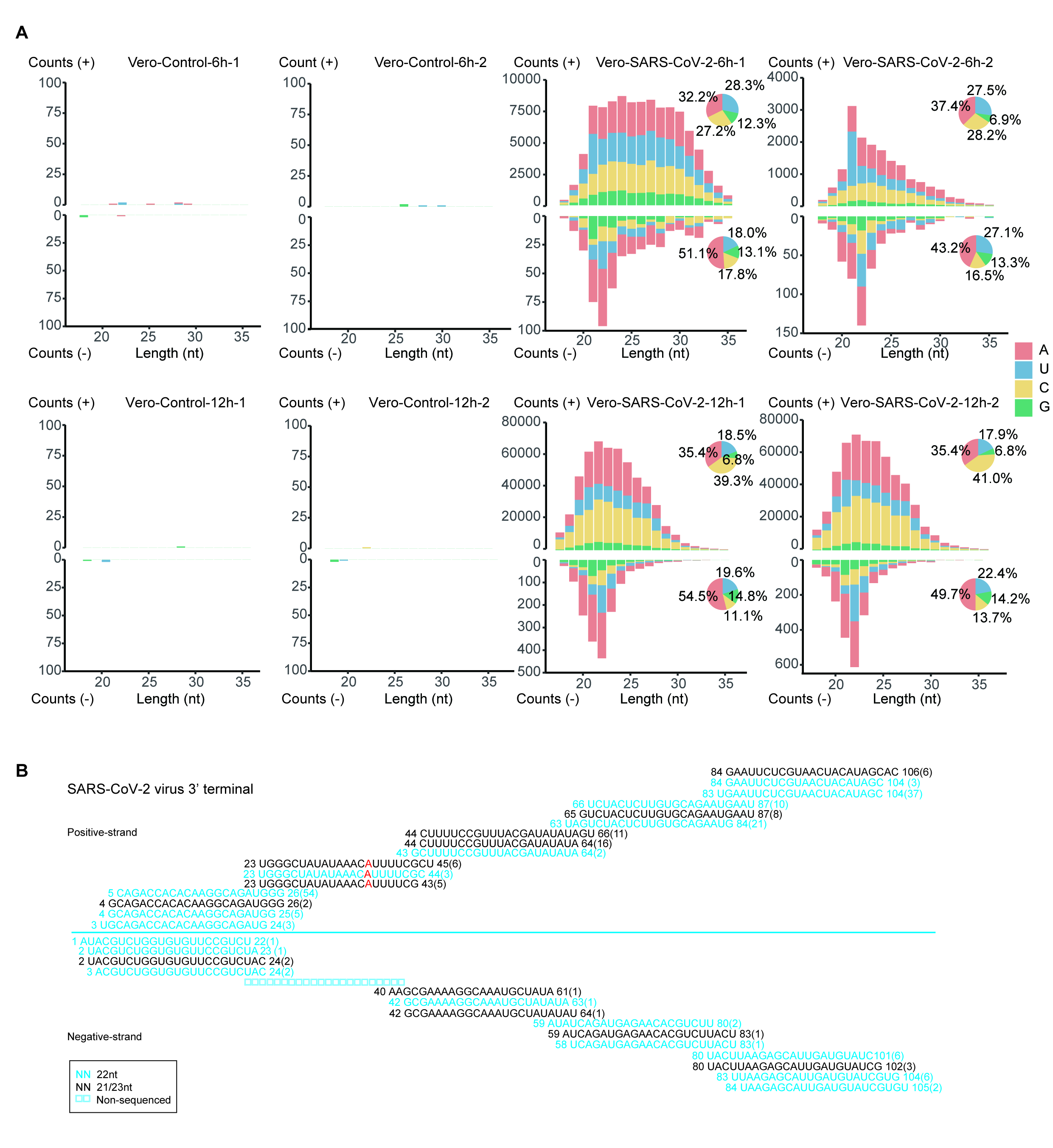

Supplement: Supplementary Figure 1 — Description of sRNA libraries in control and SARS-CoV-2-infected Vero cells. (A) Correlation of miRNA reads between two biological replicates in control and SARS-CoV-2-infected (6 hpi/12 hpi) Vero cells. (B) PCA plot of miRNA-seq libraries of control and virus-infected (6 hpi/12 hpi) Vero cells. [file DataSheet_1.zip › Data Sheet 1/SARS-CoV-2 source code & data/SARS-CoV-2 source code/fig4/Figure4.tif]

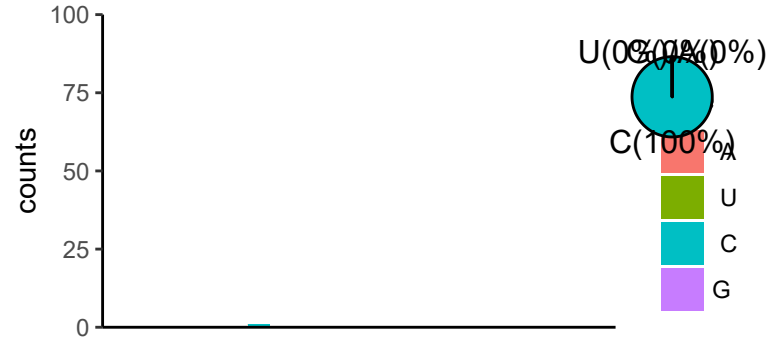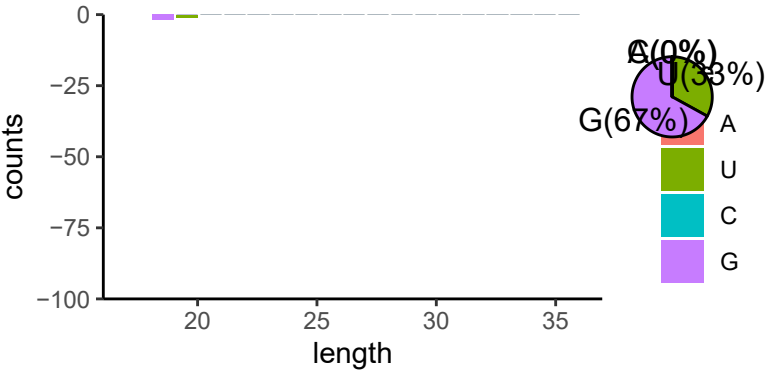

Supplement: Supplementary Figure 1 — Description of sRNA libraries in control and SARS-CoV-2-infected Vero cells. (A) Correlation of miRNA reads between two biological replicates in control and SARS-CoV-2-infected (6 hpi/12 hpi) Vero cells. (B) PCA plot of miRNA-seq libraries of control and virus-infected (6 hpi/12 hpi) Vero cells. [file DataSheet_1.zip › Data Sheet 1/SARS-CoV-2 source code & data/SARS-CoV-2 source code/fig4/output/Vero_control-12h-2.pdf]

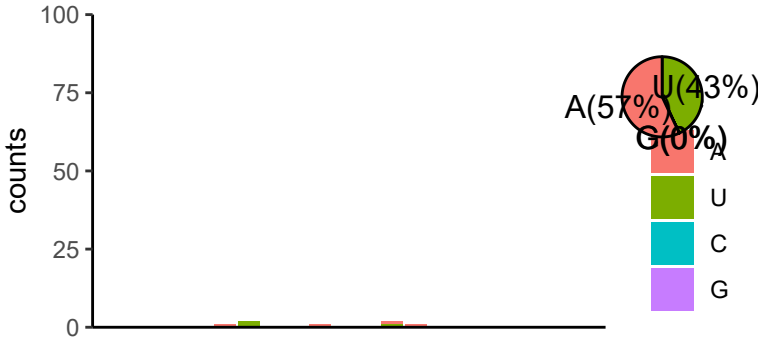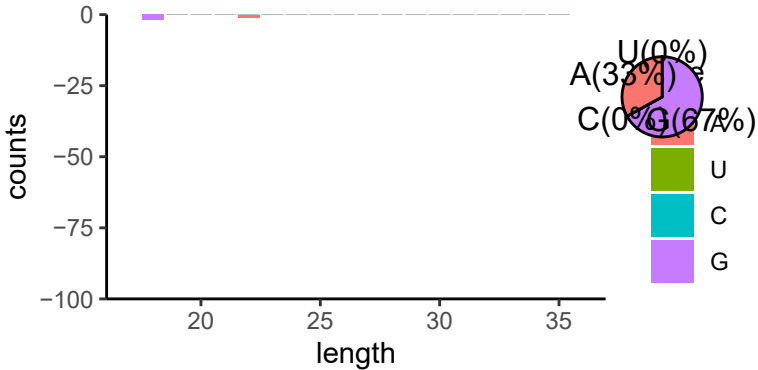

Supplement: Supplementary Figure 1 — Description of sRNA libraries in control and SARS-CoV-2-infected Vero cells. (A) Correlation of miRNA reads between two biological replicates in control and SARS-CoV-2-infected (6 hpi/12 hpi) Vero cells. (B) PCA plot of miRNA-seq libraries of control and virus-infected (6 hpi/12 hpi) Vero cells. [file DataSheet_1.zip › Data Sheet 1/SARS-CoV-2 source code & data/SARS-CoV-2 source code/fig4/output/Vero_control-6h-1.pdf]

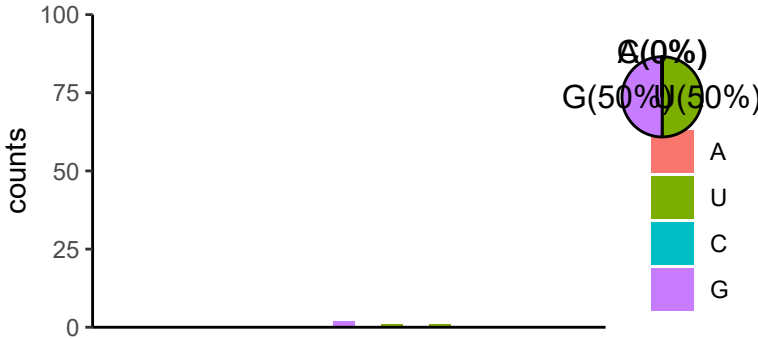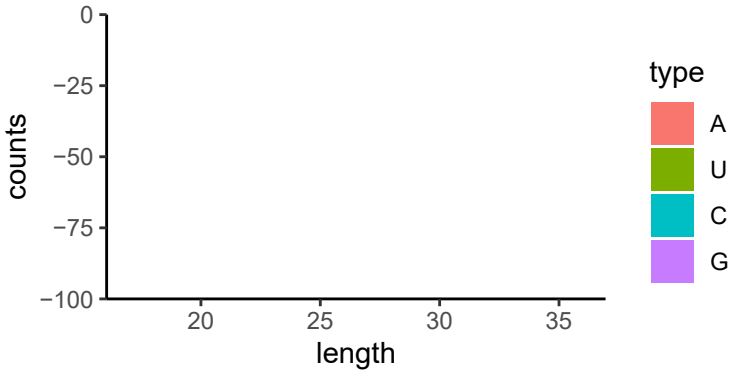

Supplement: Supplementary Figure 1 — Description of sRNA libraries in control and SARS-CoV-2-infected Vero cells. (A) Correlation of miRNA reads between two biological replicates in control and SARS-CoV-2-infected (6 hpi/12 hpi) Vero cells. (B) PCA plot of miRNA-seq libraries of control and virus-infected (6 hpi/12 hpi) Vero cells. [file DataSheet_1.zip › Data Sheet 1/SARS-CoV-2 source code & data/SARS-CoV-2 source code/fig4/output/Vero_control-6h-2.pdf]

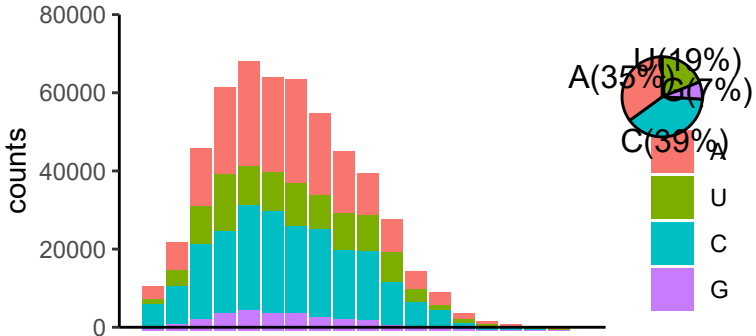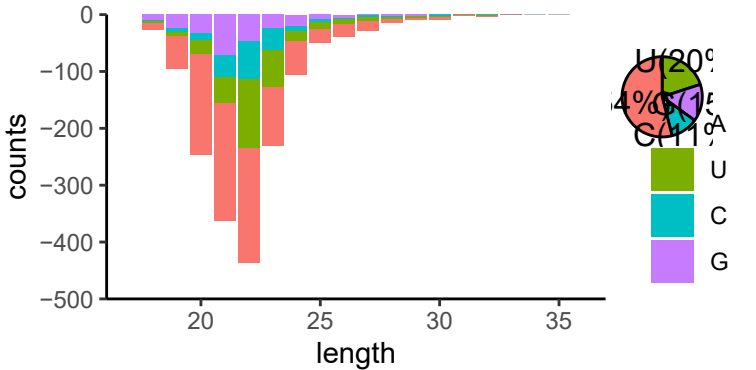

Supplement: Supplementary Figure 1 — Description of sRNA libraries in control and SARS-CoV-2-infected Vero cells. (A) Correlation of miRNA reads between two biological replicates in control and SARS-CoV-2-infected (6 hpi/12 hpi) Vero cells. (B) PCA plot of miRNA-seq libraries of control and virus-infected (6 hpi/12 hpi) Vero cells. [file DataSheet_1.zip › Data Sheet 1/SARS-CoV-2 source code & data/SARS-CoV-2 source code/fig4/output/Vero_SARS-Cov-2-12h-1.pdf]

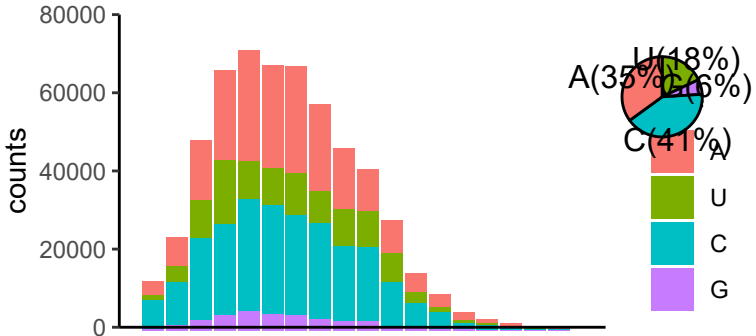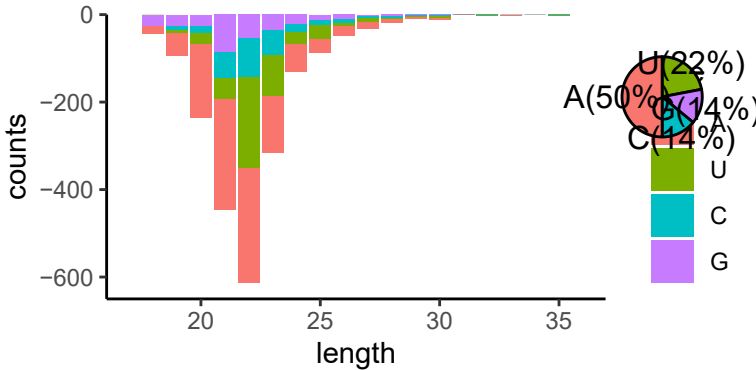

Supplement: Supplementary Figure 1 — Description of sRNA libraries in control and SARS-CoV-2-infected Vero cells. (A) Correlation of miRNA reads between two biological replicates in control and SARS-CoV-2-infected (6 hpi/12 hpi) Vero cells. (B) PCA plot of miRNA-seq libraries of control and virus-infected (6 hpi/12 hpi) Vero cells. [file DataSheet_1.zip › Data Sheet 1/SARS-CoV-2 source code & data/SARS-CoV-2 source code/fig4/output/Vero_SARS-Cov-2-12h-2.pdf]

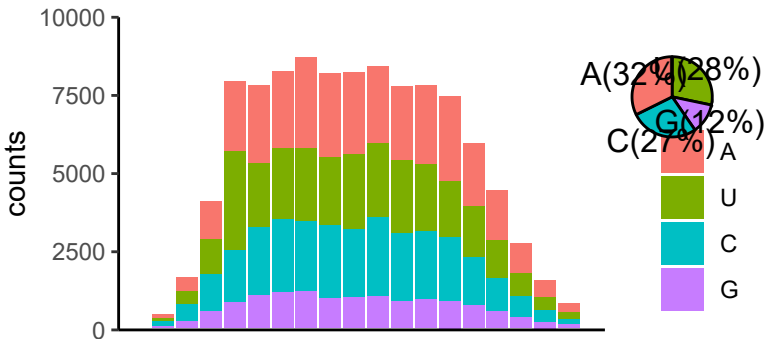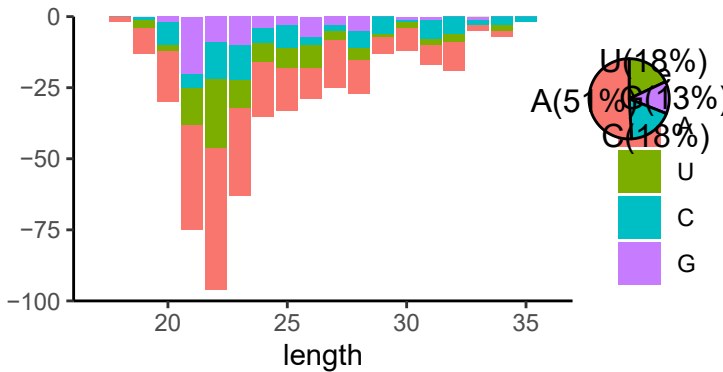

Supplement: Supplementary Figure 1 — Description of sRNA libraries in control and SARS-CoV-2-infected Vero cells. (A) Correlation of miRNA reads between two biological replicates in control and SARS-CoV-2-infected (6 hpi/12 hpi) Vero cells. (B) PCA plot of miRNA-seq libraries of control and virus-infected (6 hpi/12 hpi) Vero cells. [file DataSheet_1.zip › Data Sheet 1/SARS-CoV-2 source code & data/SARS-CoV-2 source code/fig4/output/Vero_SARS-Cov-2-6h-1.pdf]

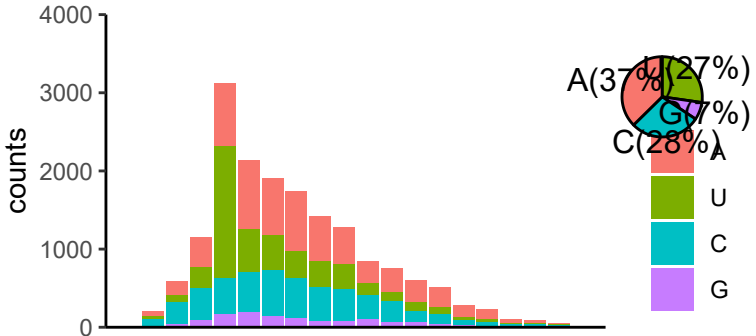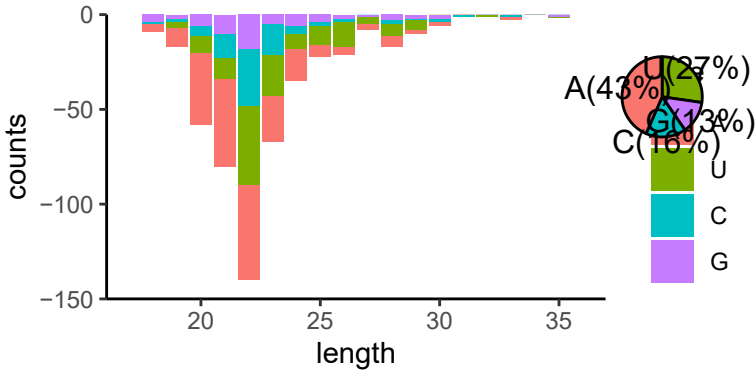

Supplement: Supplementary Figure 1 — Description of sRNA libraries in control and SARS-CoV-2-infected Vero cells. (A) Correlation of miRNA reads between two biological replicates in control and SARS-CoV-2-infected (6 hpi/12 hpi) Vero cells. (B) PCA plot of miRNA-seq libraries of control and virus-infected (6 hpi/12 hpi) Vero cells. [file DataSheet_1.zip › Data Sheet 1/SARS-CoV-2 source code & data/SARS-CoV-2 source code/fig4/output/Vero_SARS-Cov-2-6h-2.pdf]

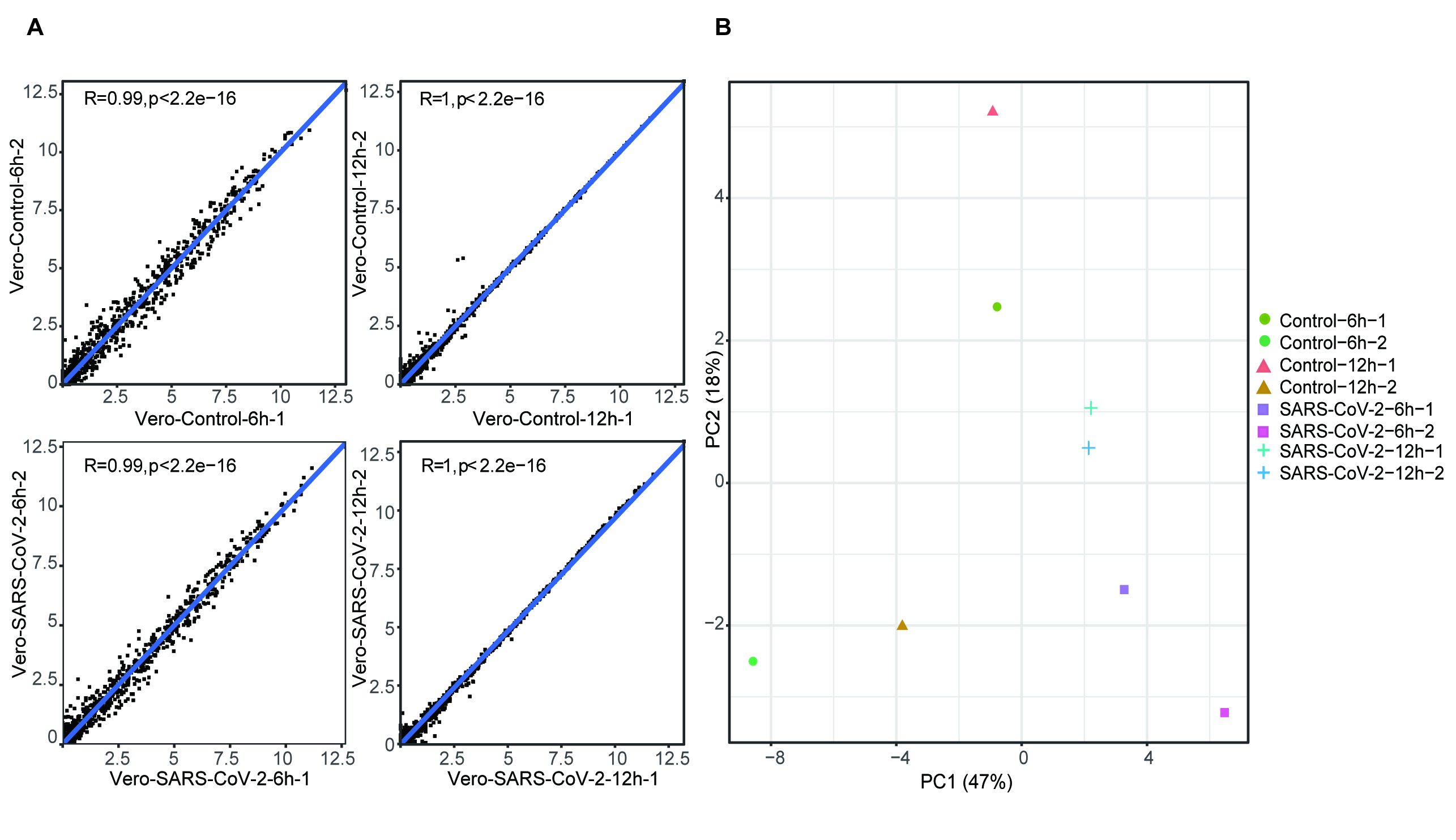

Supplement: Supplementary Figure 1 — Description of sRNA libraries in control and SARS-CoV-2-infected Vero cells. (A) Correlation of miRNA reads between two biological replicates in control and SARS-CoV-2-infected (6 hpi/12 hpi) Vero cells. (B) PCA plot of miRNA-seq libraries of control and virus-infected (6 hpi/12 hpi) Vero cells. [file DataSheet_1.zip › Data Sheet 1/SARS-CoV-2 source code & data/SARS-CoV-2 source code/figs1/FigureS1.tif]

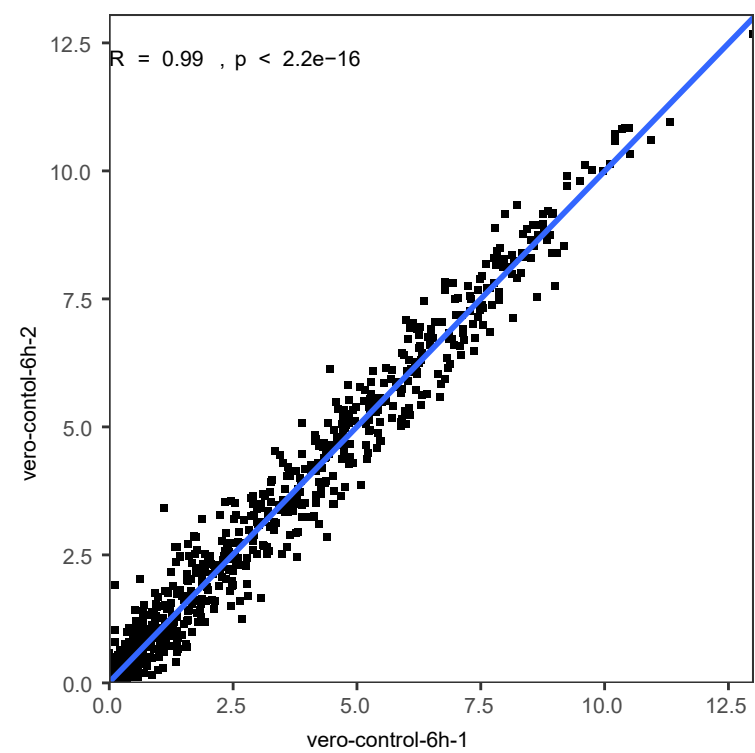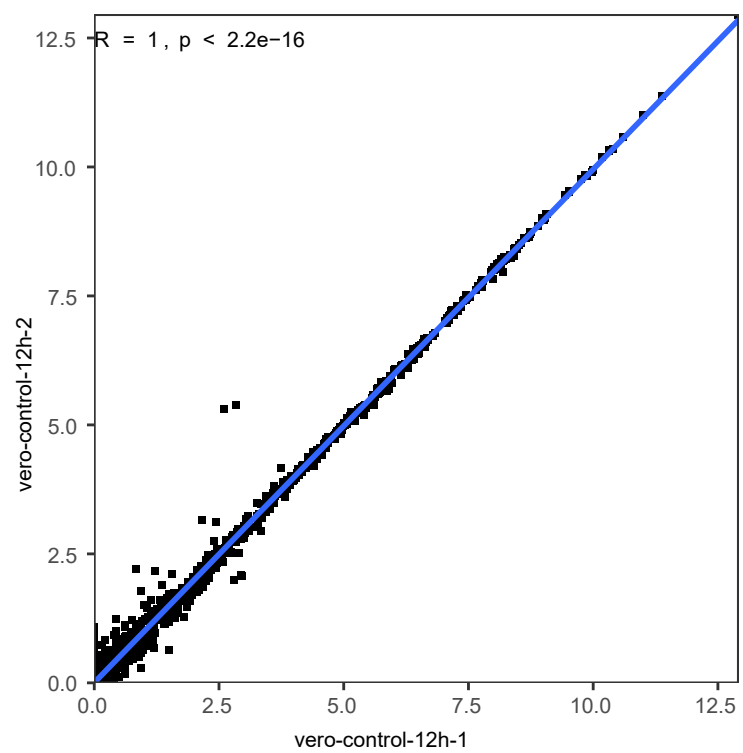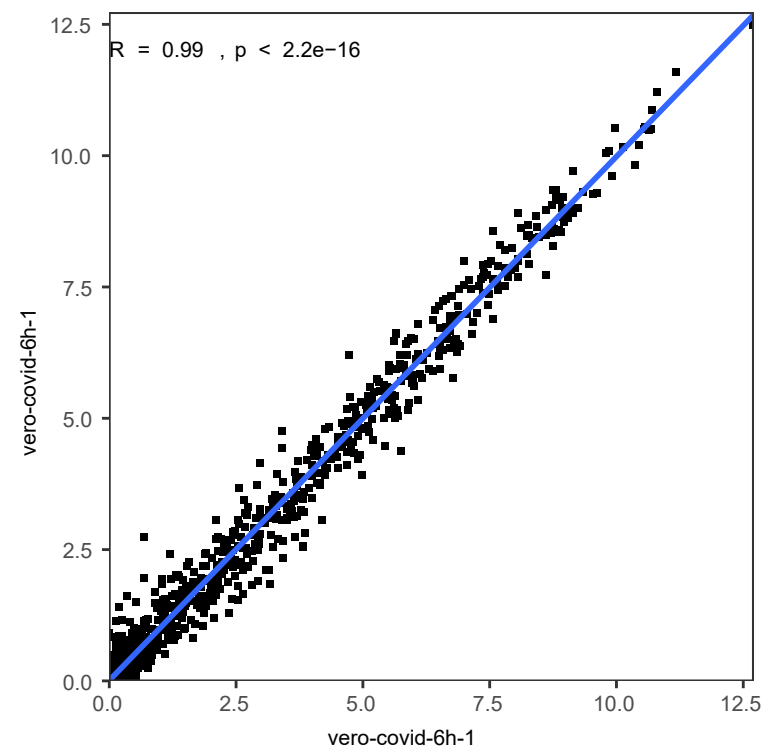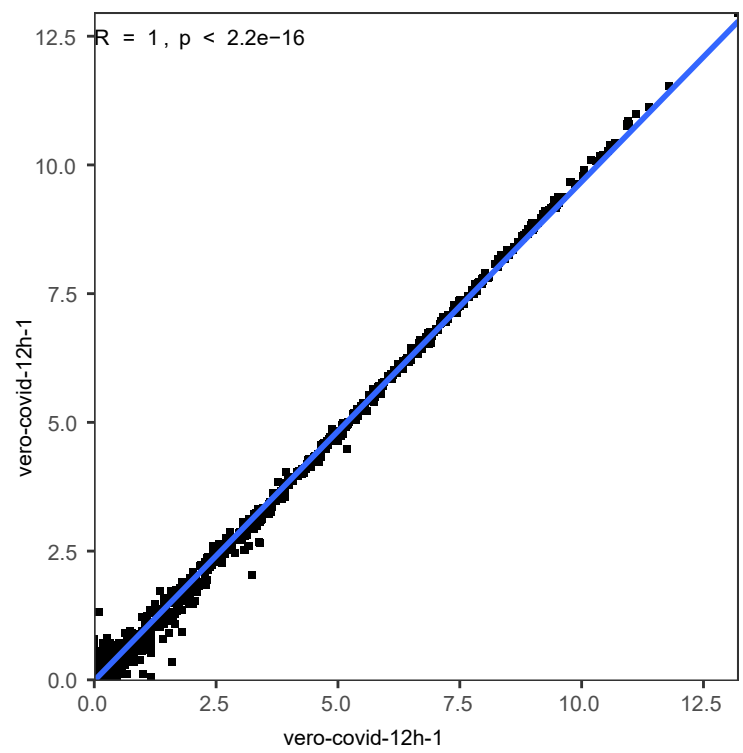

Supplement: Supplementary Figure 1 — Description of sRNA libraries in control and SARS-CoV-2-infected Vero cells. (A) Correlation of miRNA reads between two biological replicates in control and SARS-CoV-2-infected (6 hpi/12 hpi) Vero cells. (B) PCA plot of miRNA-seq libraries of control and virus-infected (6 hpi/12 hpi) Vero cells. [file DataSheet_1.zip › Data Sheet 1/SARS-CoV-2 source code & data/SARS-CoV-2 source code/figs1/output/corelation.pdf]

# DESeq2 PCA

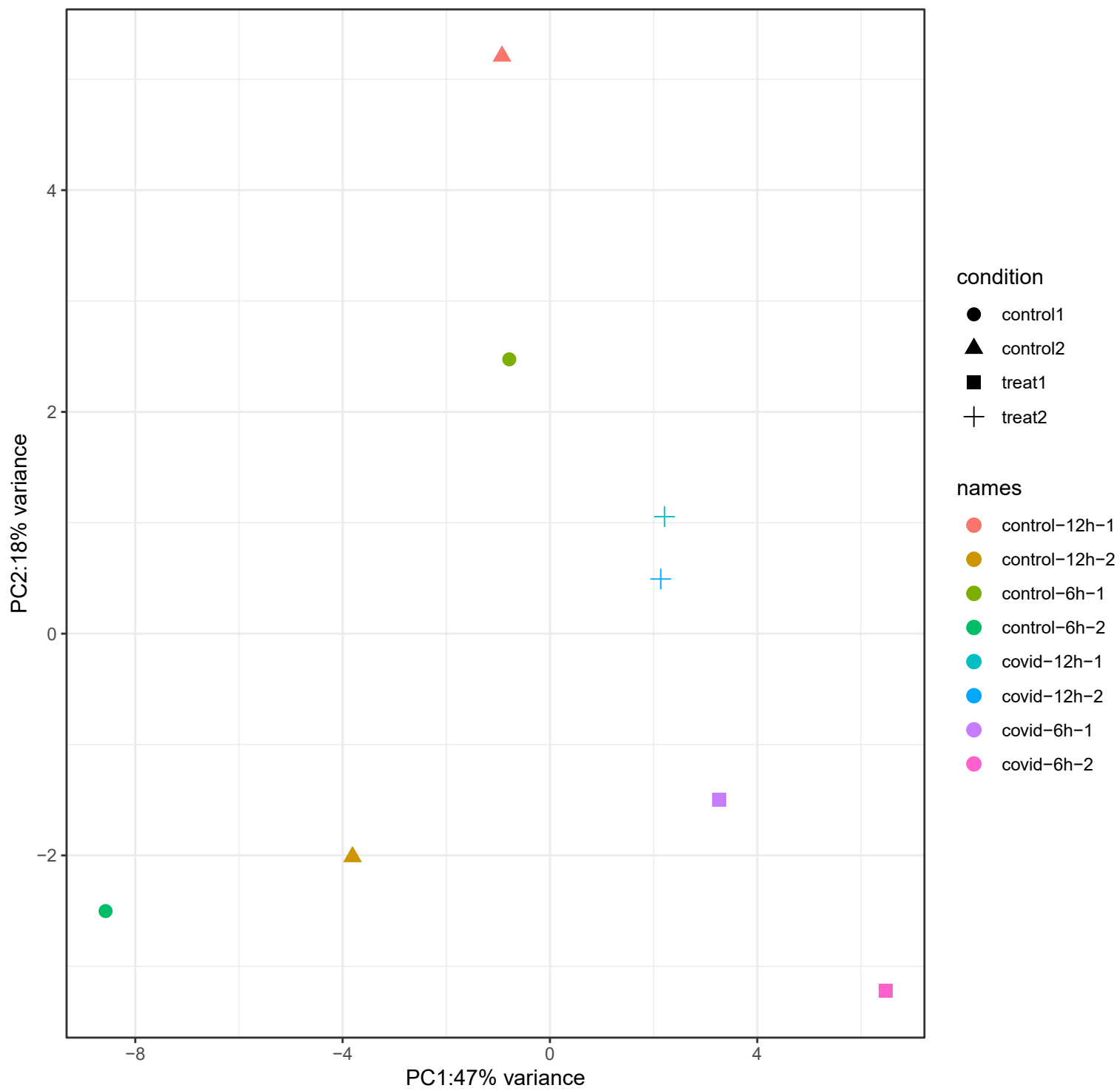

Supplement: Supplementary Figure 1 — Description of sRNA libraries in control and SARS-CoV-2-infected Vero cells. (A) Correlation of miRNA reads between two biological replicates in control and SARS-CoV-2-infected (6 hpi/12 hpi) Vero cells. (B) PCA plot of miRNA-seq libraries of control and virus-infected (6 hpi/12 hpi) Vero cells. [file DataSheet_1.zip › Data Sheet 1/SARS-CoV-2 source code & data/SARS-CoV-2 source code/figs1/output/pca.pdf]

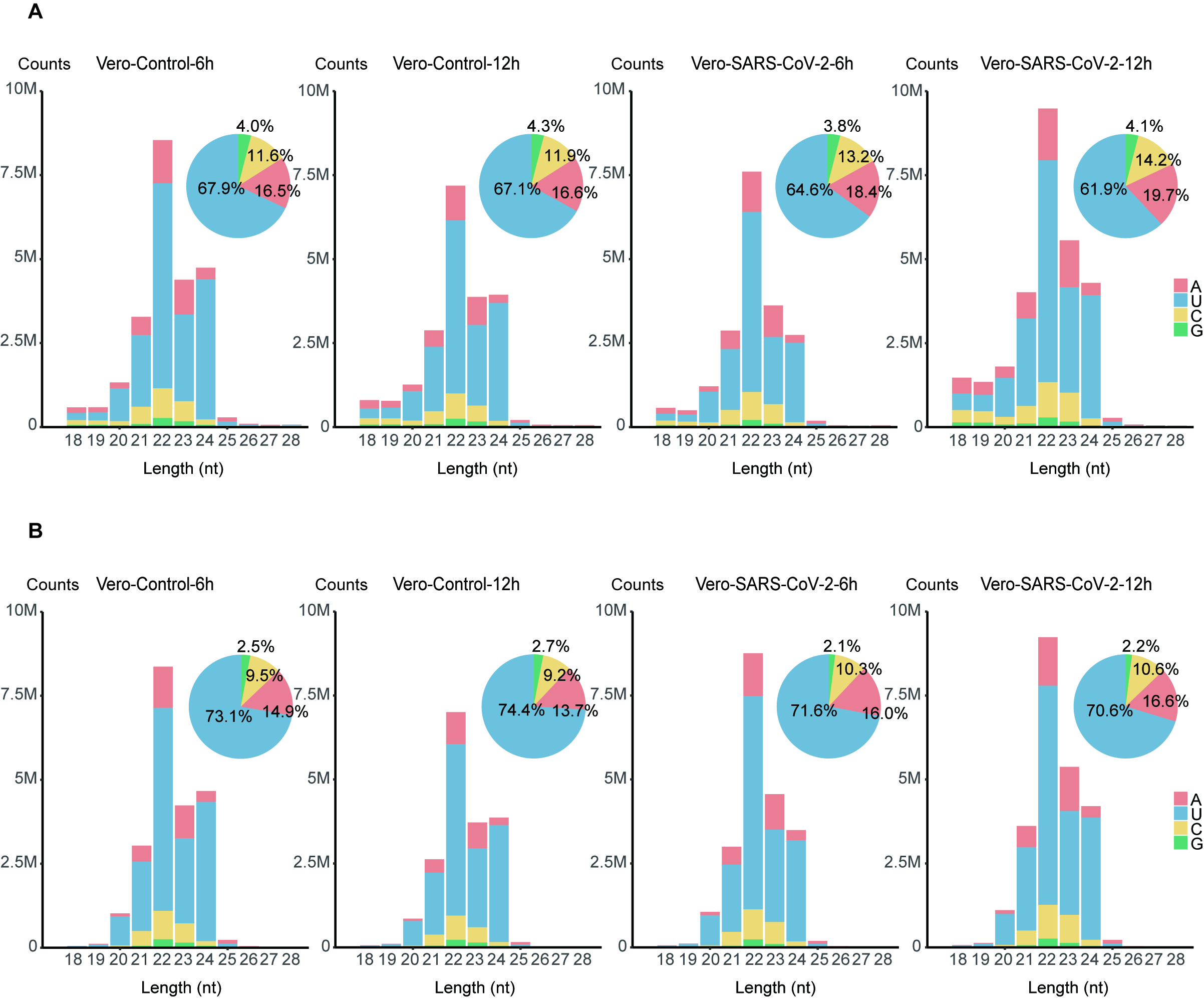

Supplement: Supplementary Figure 1 — Description of sRNA libraries in control and SARS-CoV-2-infected Vero cells. (A) Correlation of miRNA reads between two biological replicates in control and SARS-CoV-2-infected (6 hpi/12 hpi) Vero cells. (B) PCA plot of miRNA-seq libraries of control and virus-infected (6 hpi/12 hpi) Vero cells. [file DataSheet_1.zip › Data Sheet 1/SARS-CoV-2 source code & data/SARS-CoV-2 source code/figs2/FigureS2.tif]

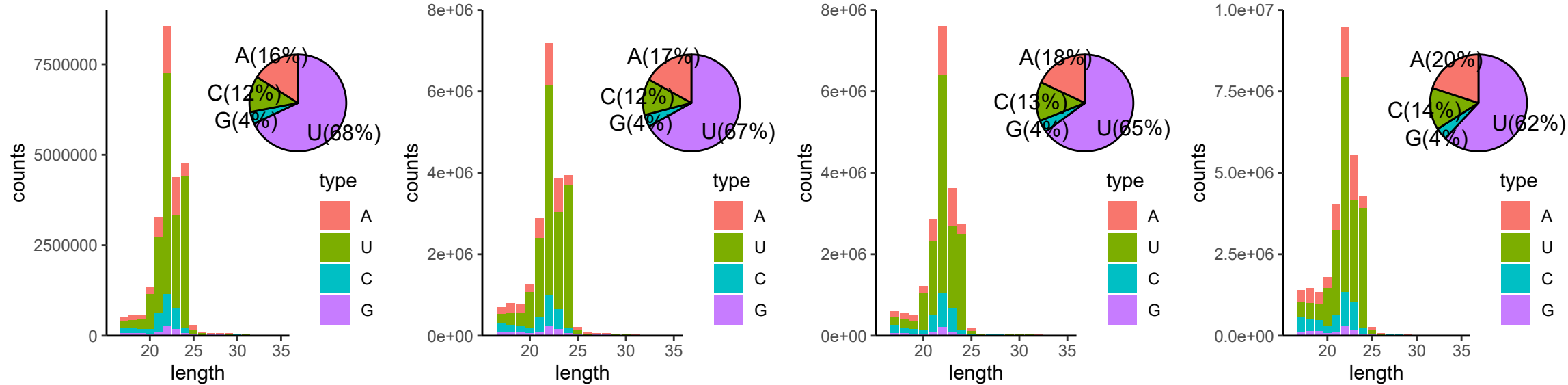

Supplement: Supplementary Figure 1 — Description of sRNA libraries in control and SARS-CoV-2-infected Vero cells. (A) Correlation of miRNA reads between two biological replicates in control and SARS-CoV-2-infected (6 hpi/12 hpi) Vero cells. (B) PCA plot of miRNA-seq libraries of control and virus-infected (6 hpi/12 hpi) Vero cells. [file DataSheet_1.zip › Data Sheet 1/SARS-CoV-2 source code & data/SARS-CoV-2 source code/figs2/output/Figure S2A.pdf]

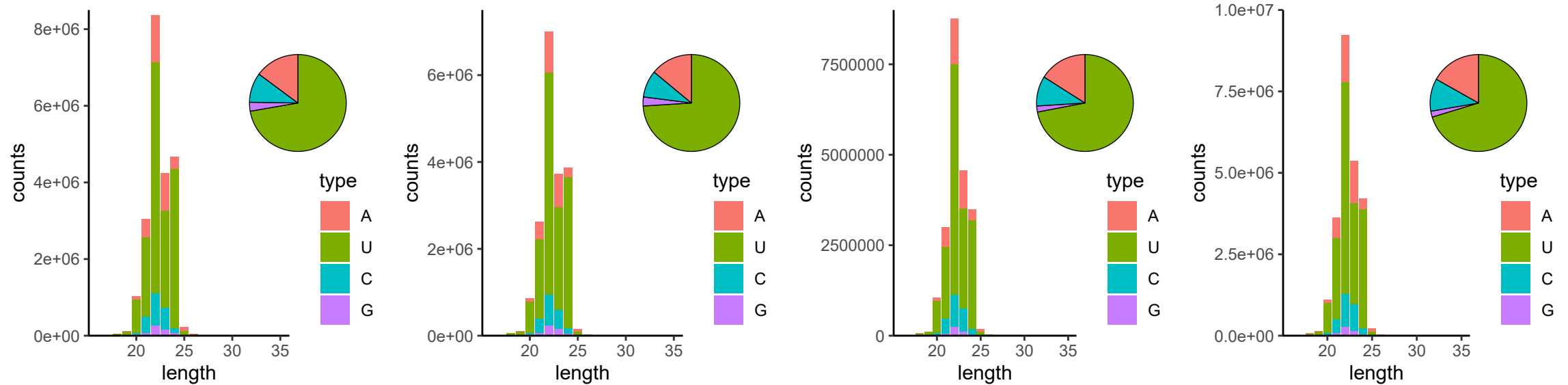

Supplement: Supplementary Figure 1 — Description of sRNA libraries in control and SARS-CoV-2-infected Vero cells. (A) Correlation of miRNA reads between two biological replicates in control and SARS-CoV-2-infected (6 hpi/12 hpi) Vero cells. (B) PCA plot of miRNA-seq libraries of control and virus-infected (6 hpi/12 hpi) Vero cells. [file DataSheet_1.zip › Data Sheet 1/SARS-CoV-2 source code & data/SARS-CoV-2 source code/figs2/output/Figure S2B.pdf]

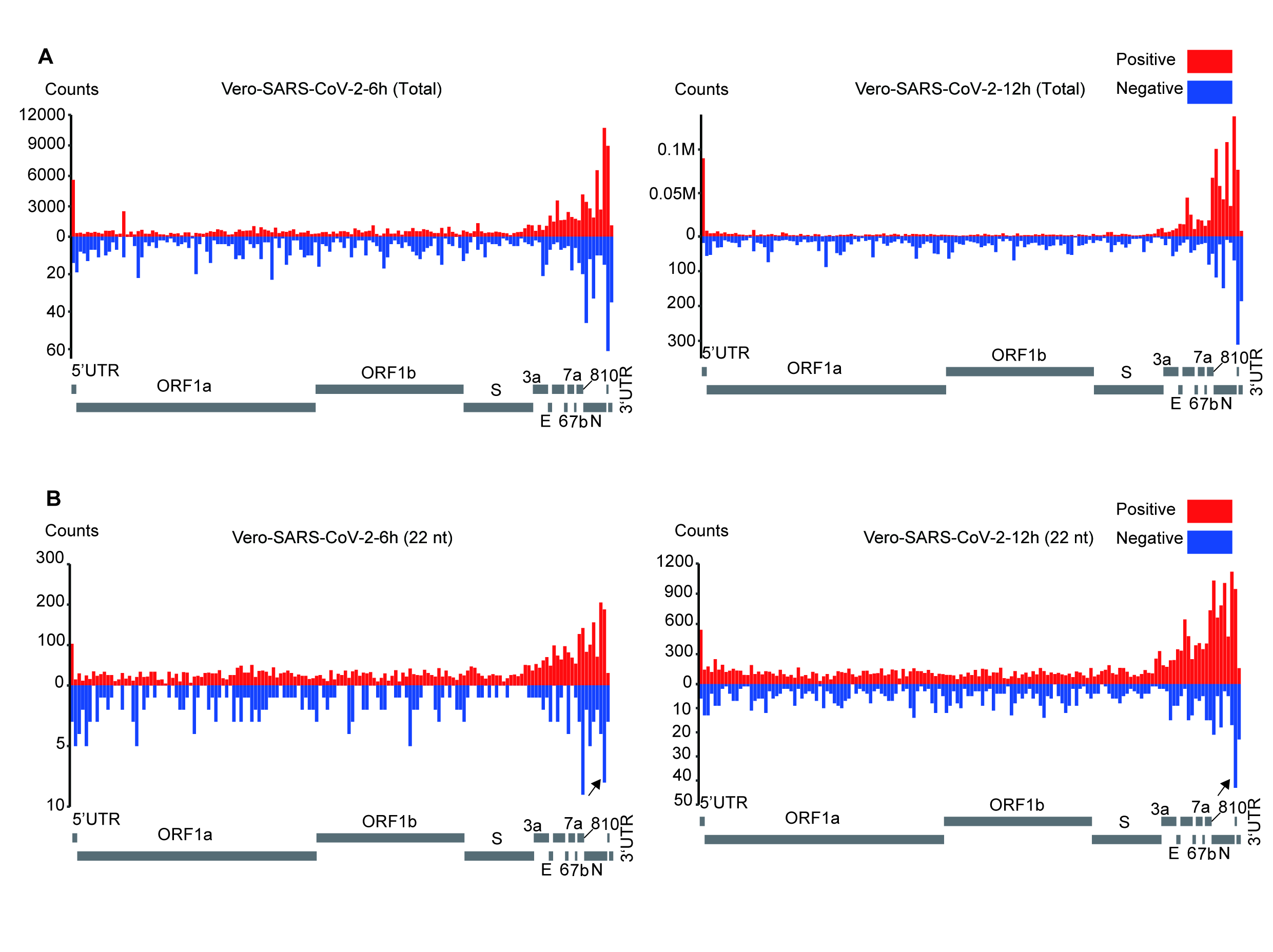

Supplement: Supplementary Figure 1 — Description of sRNA libraries in control and SARS-CoV-2-infected Vero cells. (A) Correlation of miRNA reads between two biological replicates in control and SARS-CoV-2-infected (6 hpi/12 hpi) Vero cells. (B) PCA plot of miRNA-seq libraries of control and virus-infected (6 hpi/12 hpi) Vero cells. [file DataSheet_1.zip › Data Sheet 1/SARS-CoV-2 source code & data/SARS-CoV-2 source code/figs3/FigureS3.tif]

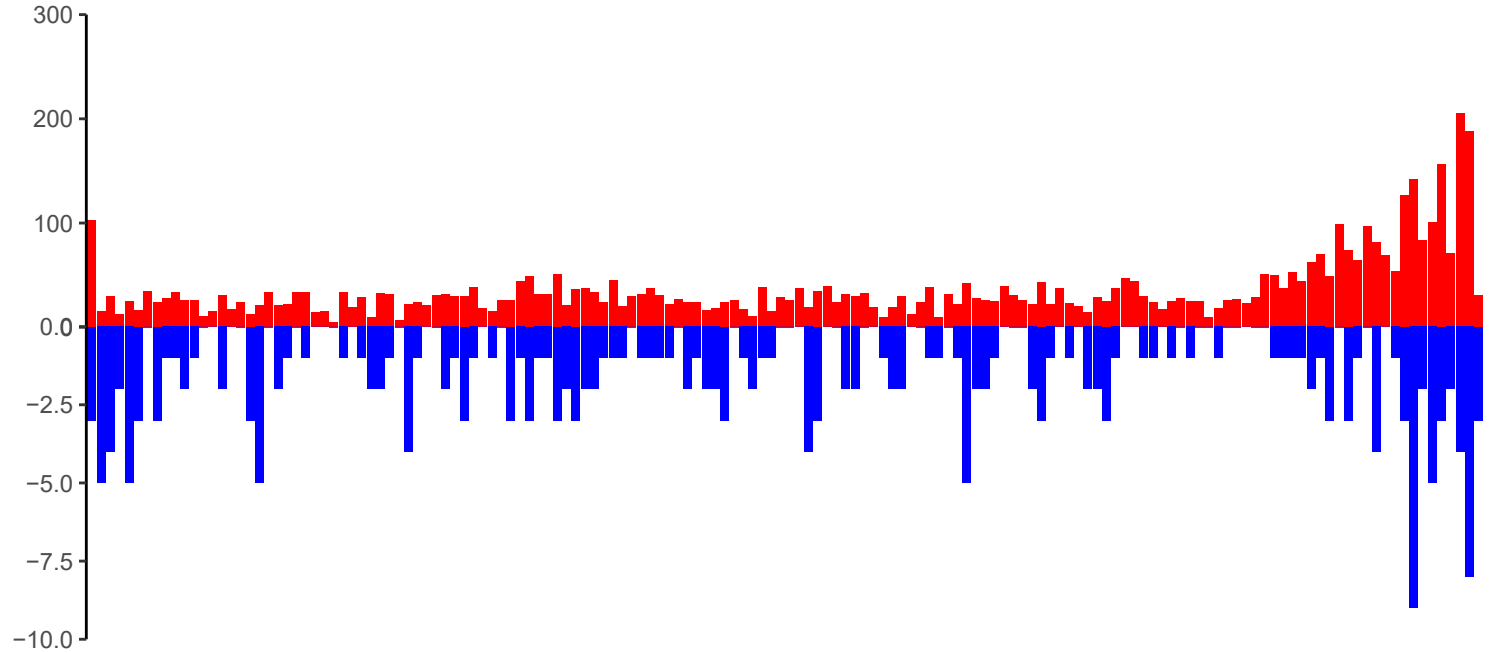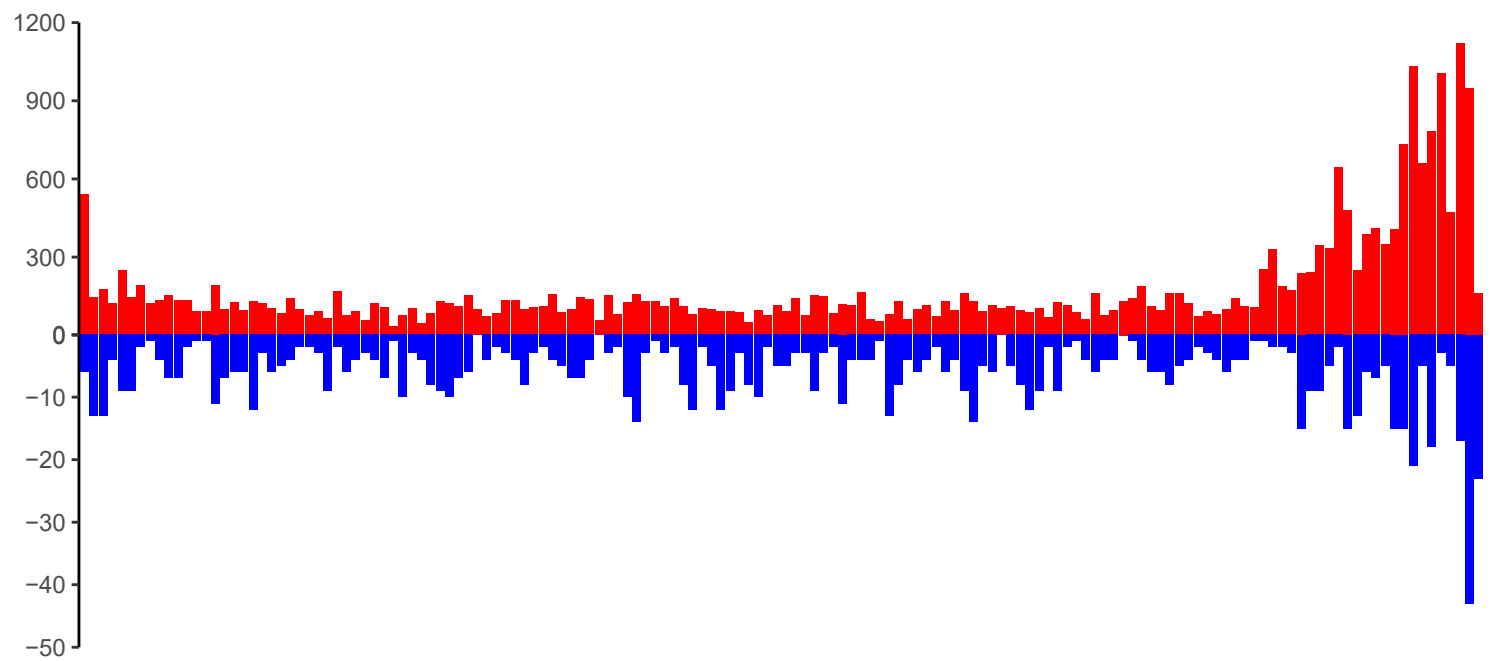

Supplement: Supplementary Figure 1 — Description of sRNA libraries in control and SARS-CoV-2-infected Vero cells. (A) Correlation of miRNA reads between two biological replicates in control and SARS-CoV-2-infected (6 hpi/12 hpi) Vero cells. (B) PCA plot of miRNA-seq libraries of control and virus-infected (6 hpi/12 hpi) Vero cells. [file DataSheet_1.zip › Data Sheet 1/SARS-CoV-2 source code & data/SARS-CoV-2 source code/figs3/output/22nt.pdf]

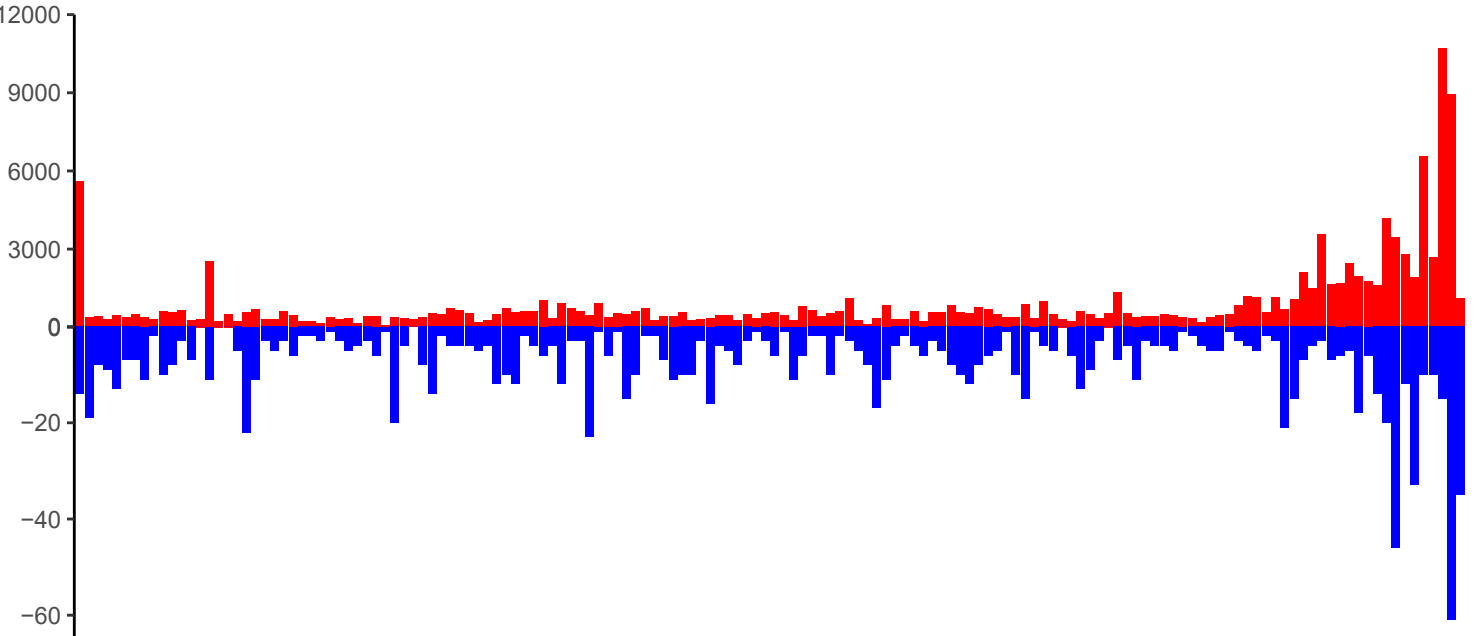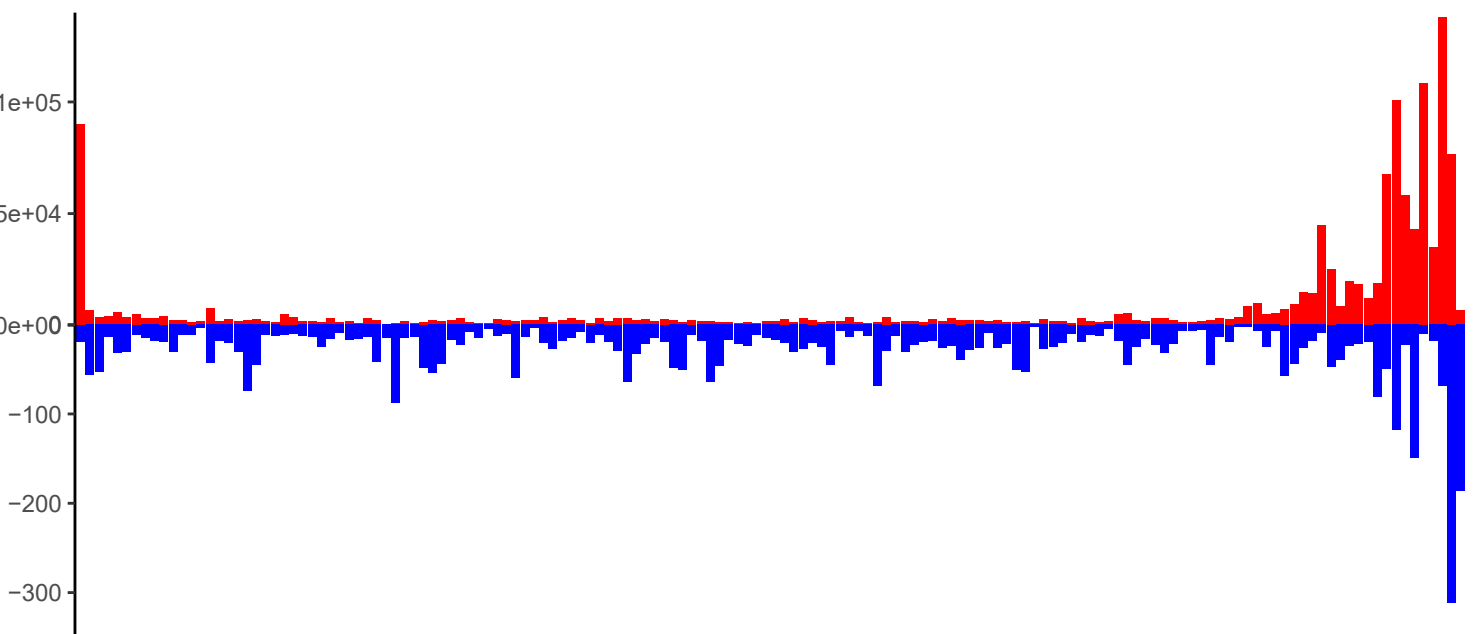

Supplement: Supplementary Figure 1 — Description of sRNA libraries in control and SARS-CoV-2-infected Vero cells. (A) Correlation of miRNA reads between two biological replicates in control and SARS-CoV-2-infected (6 hpi/12 hpi) Vero cells. (B) PCA plot of miRNA-seq libraries of control and virus-infected (6 hpi/12 hpi) Vero cells. [file DataSheet_1.zip › Data Sheet 1/SARS-CoV-2 source code & data/SARS-CoV-2 source code/figs3/output/vsi.pdf]

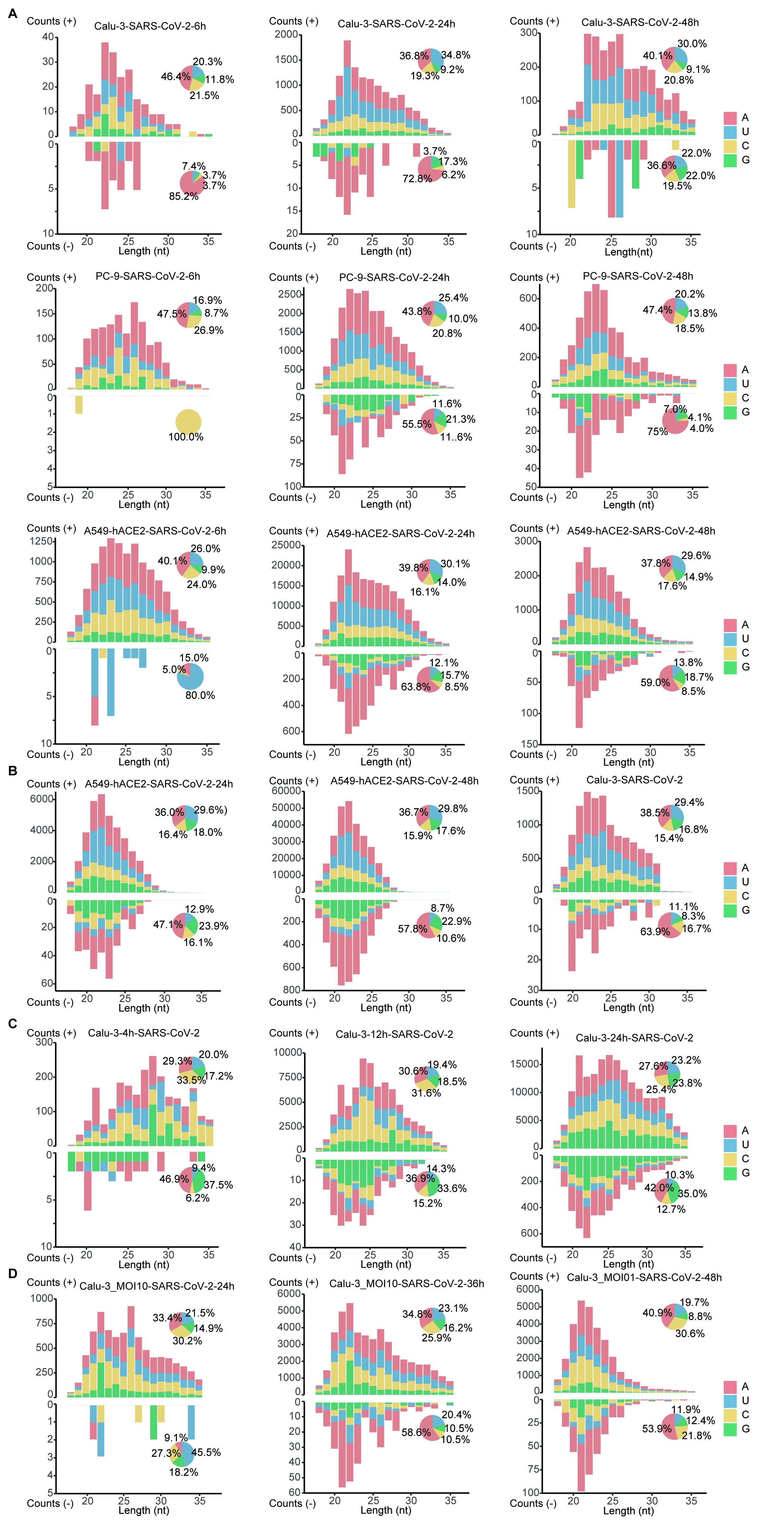

Supplement: Supplementary Figure 1 — Description of sRNA libraries in control and SARS-CoV-2-infected Vero cells. (A) Correlation of miRNA reads between two biological replicates in control and SARS-CoV-2-infected (6 hpi/12 hpi) Vero cells. (B) PCA plot of miRNA-seq libraries of control and virus-infected (6 hpi/12 hpi) Vero cells. [file DataSheet_1.zip › Data Sheet 1/SARS-CoV-2 source code & data/SARS-CoV-2 source code/figs4/FigureS4.tif]

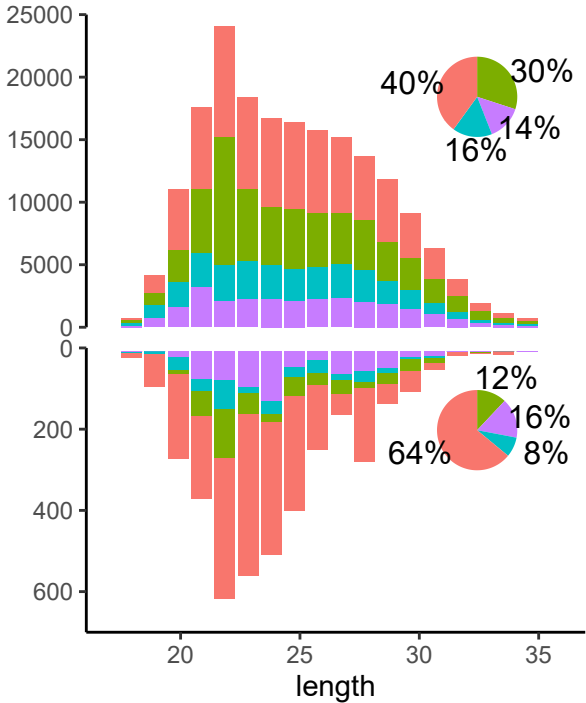

Supplement: Supplementary Figure 1 — Description of sRNA libraries in control and SARS-CoV-2-infected Vero cells. (A) Correlation of miRNA reads between two biological replicates in control and SARS-CoV-2-infected (6 hpi/12 hpi) Vero cells. (B) PCA plot of miRNA-seq libraries of control and virus-infected (6 hpi/12 hpi) Vero cells. [file DataSheet_1.zip › Data Sheet 1/SARS-CoV-2 source code & data/SARS-CoV-2 source code/figs4/output/Figure S4A-A549-hACE2-SARS-CoV-2-24h.pdf]

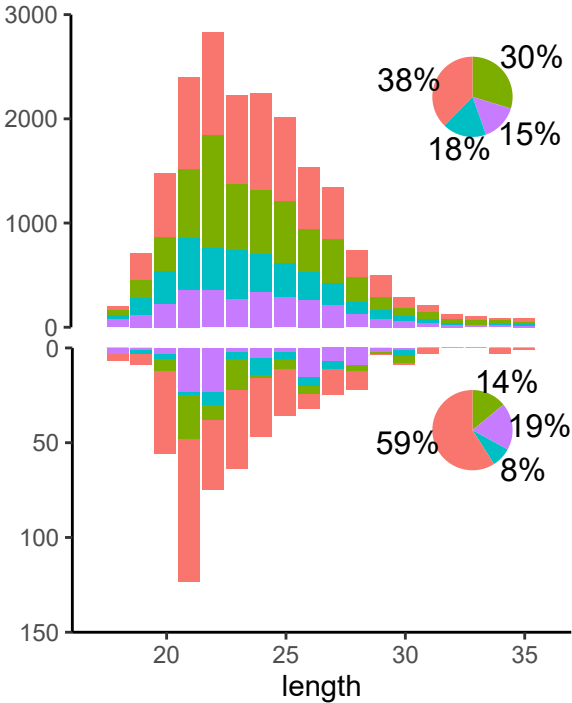

Supplement: Supplementary Figure 1 — Description of sRNA libraries in control and SARS-CoV-2-infected Vero cells. (A) Correlation of miRNA reads between two biological replicates in control and SARS-CoV-2-infected (6 hpi/12 hpi) Vero cells. (B) PCA plot of miRNA-seq libraries of control and virus-infected (6 hpi/12 hpi) Vero cells. [file DataSheet_1.zip › Data Sheet 1/SARS-CoV-2 source code & data/SARS-CoV-2 source code/figs4/output/Figure S4A-A549-hACE2-SARS-CoV-2-48h.pdf]

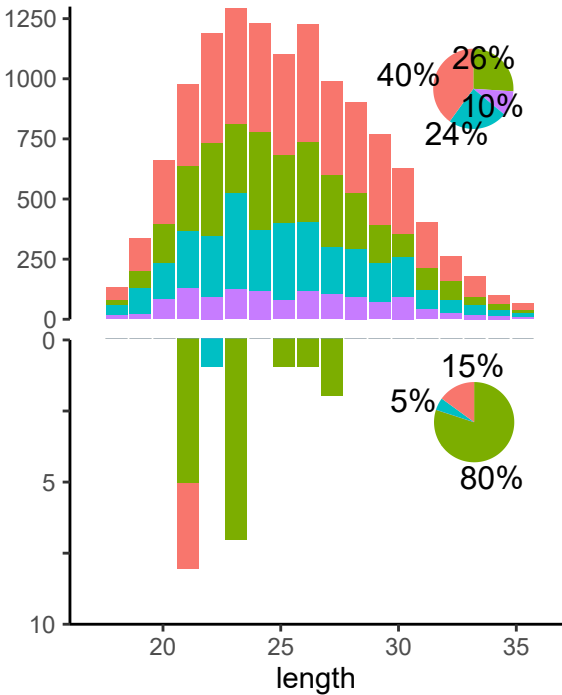

Supplement: Supplementary Figure 1 — Description of sRNA libraries in control and SARS-CoV-2-infected Vero cells. (A) Correlation of miRNA reads between two biological replicates in control and SARS-CoV-2-infected (6 hpi/12 hpi) Vero cells. (B) PCA plot of miRNA-seq libraries of control and virus-infected (6 hpi/12 hpi) Vero cells. [file DataSheet_1.zip › Data Sheet 1/SARS-CoV-2 source code & data/SARS-CoV-2 source code/figs4/output/Figure S4A-A549-hACE2-SARS-CoV-2-6h.pdf]

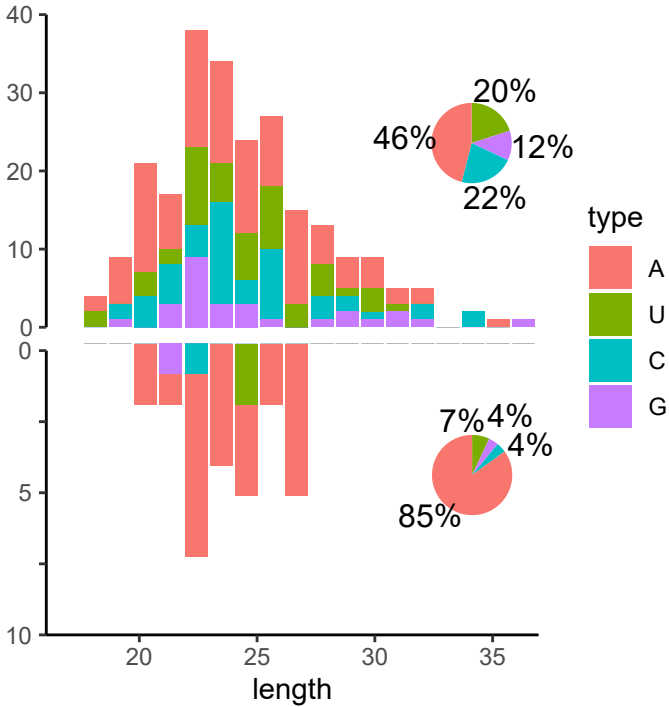

Supplement: Supplementary Figure 1 — Description of sRNA libraries in control and SARS-CoV-2-infected Vero cells. (A) Correlation of miRNA reads between two biological replicates in control and SARS-CoV-2-infected (6 hpi/12 hpi) Vero cells. (B) PCA plot of miRNA-seq libraries of control and virus-infected (6 hpi/12 hpi) Vero cells. [file DataSheet_1.zip › Data Sheet 1/SARS-CoV-2 source code & data/SARS-CoV-2 source code/figs4/output/Figure S4A-Calu-3-SARS-CoV-2-6h.pdf]

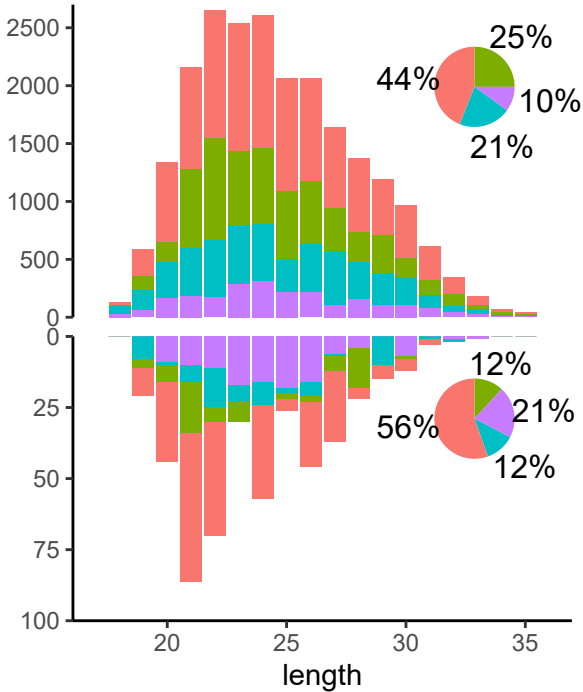

Supplement: Supplementary Figure 1 — Description of sRNA libraries in control and SARS-CoV-2-infected Vero cells. (A) Correlation of miRNA reads between two biological replicates in control and SARS-CoV-2-infected (6 hpi/12 hpi) Vero cells. (B) PCA plot of miRNA-seq libraries of control and virus-infected (6 hpi/12 hpi) Vero cells. [file DataSheet_1.zip › Data Sheet 1/SARS-CoV-2 source code & data/SARS-CoV-2 source code/figs4/output/Figure S4A-PC-9-SARS-CoV-2-24h.pdf]

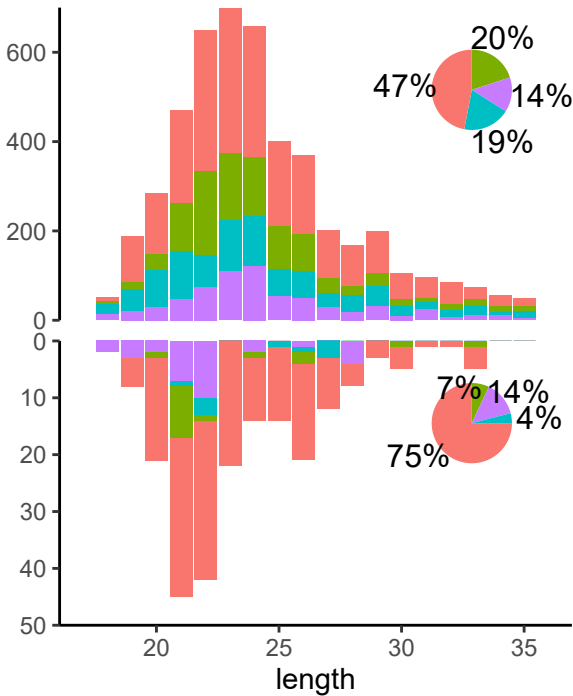

Supplement: Supplementary Figure 1 — Description of sRNA libraries in control and SARS-CoV-2-infected Vero cells. (A) Correlation of miRNA reads between two biological replicates in control and SARS-CoV-2-infected (6 hpi/12 hpi) Vero cells. (B) PCA plot of miRNA-seq libraries of control and virus-infected (6 hpi/12 hpi) Vero cells. [file DataSheet_1.zip › Data Sheet 1/SARS-CoV-2 source code & data/SARS-CoV-2 source code/figs4/output/Figure S4A-PC-9-SARS-CoV-2-48h.pdf]

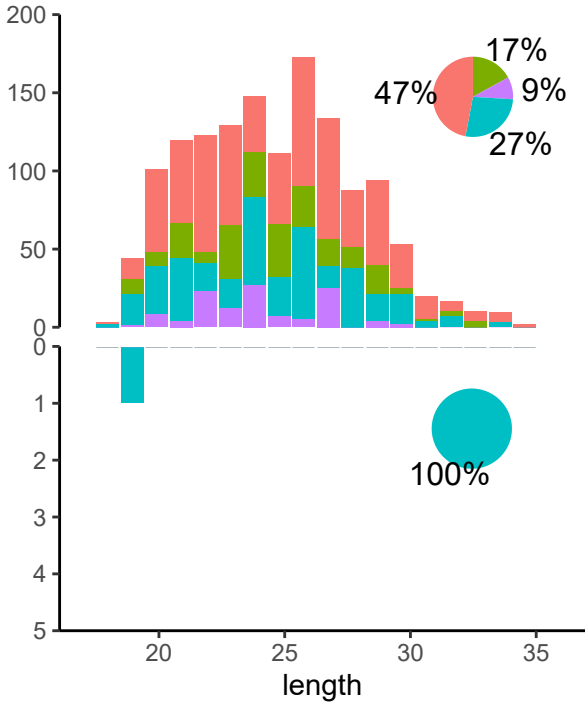

Supplement: Supplementary Figure 1 — Description of sRNA libraries in control and SARS-CoV-2-infected Vero cells. (A) Correlation of miRNA reads between two biological replicates in control and SARS-CoV-2-infected (6 hpi/12 hpi) Vero cells. (B) PCA plot of miRNA-seq libraries of control and virus-infected (6 hpi/12 hpi) Vero cells. [file DataSheet_1.zip › Data Sheet 1/SARS-CoV-2 source code & data/SARS-CoV-2 source code/figs4/output/Figure S4A-PC-9-SARS-CoV-2-6h.pdf]

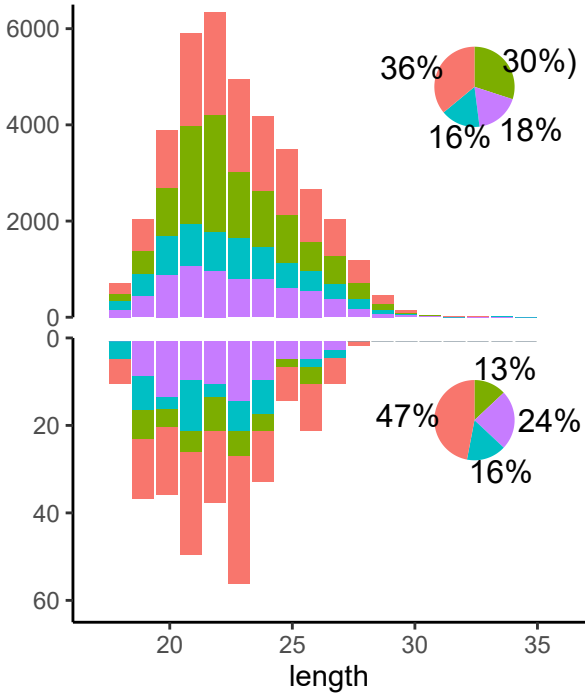

Supplement: Supplementary Figure 1 — Description of sRNA libraries in control and SARS-CoV-2-infected Vero cells. (A) Correlation of miRNA reads between two biological replicates in control and SARS-CoV-2-infected (6 hpi/12 hpi) Vero cells. (B) PCA plot of miRNA-seq libraries of control and virus-infected (6 hpi/12 hpi) Vero cells. [file DataSheet_1.zip › Data Sheet 1/SARS-CoV-2 source code & data/SARS-CoV-2 source code/figs4/output/Figure S4B-A549-hACE2-SARS-CoV-2-24h.pdf]

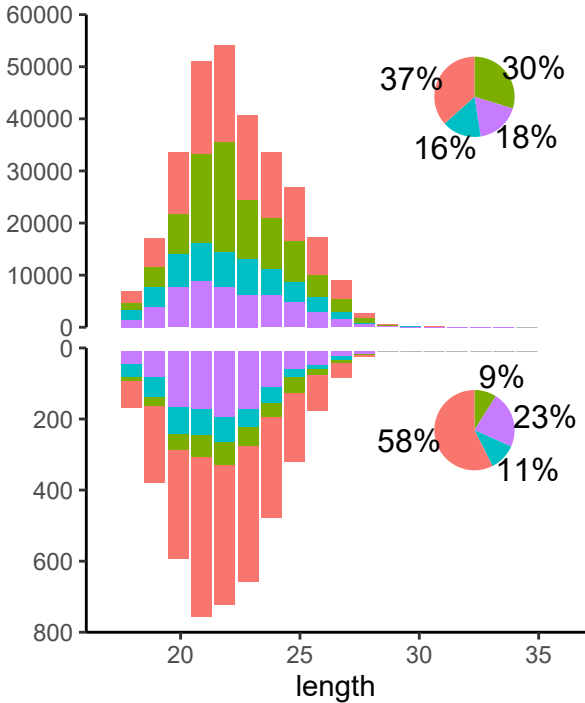

Supplement: Supplementary Figure 1 — Description of sRNA libraries in control and SARS-CoV-2-infected Vero cells. (A) Correlation of miRNA reads between two biological replicates in control and SARS-CoV-2-infected (6 hpi/12 hpi) Vero cells. (B) PCA plot of miRNA-seq libraries of control and virus-infected (6 hpi/12 hpi) Vero cells. [file DataSheet_1.zip › Data Sheet 1/SARS-CoV-2 source code & data/SARS-CoV-2 source code/figs4/output/Figure S4B-A549-hACE2-SARS-CoV-2-48h.pdf]

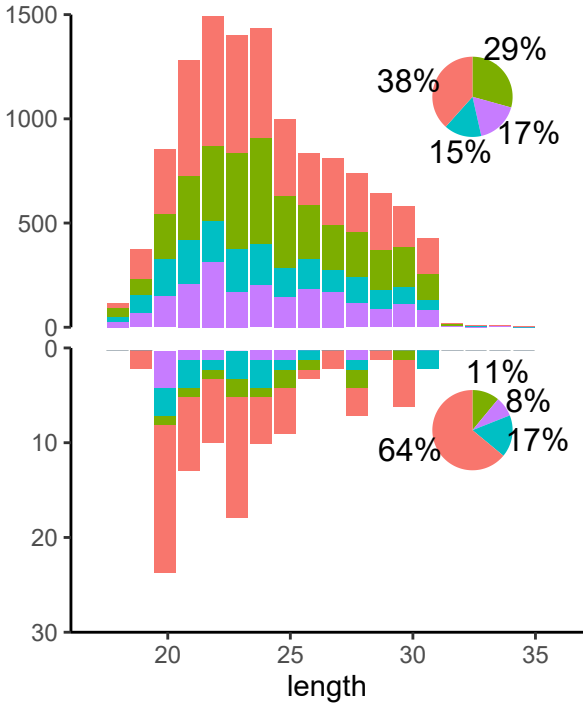

Supplement: Supplementary Figure 1 — Description of sRNA libraries in control and SARS-CoV-2-infected Vero cells. (A) Correlation of miRNA reads between two biological replicates in control and SARS-CoV-2-infected (6 hpi/12 hpi) Vero cells. (B) PCA plot of miRNA-seq libraries of control and virus-infected (6 hpi/12 hpi) Vero cells. [file DataSheet_1.zip › Data Sheet 1/SARS-CoV-2 source code & data/SARS-CoV-2 source code/figs4/output/Figure S4B-Calu-3-SARS-CoV-2.pdf]

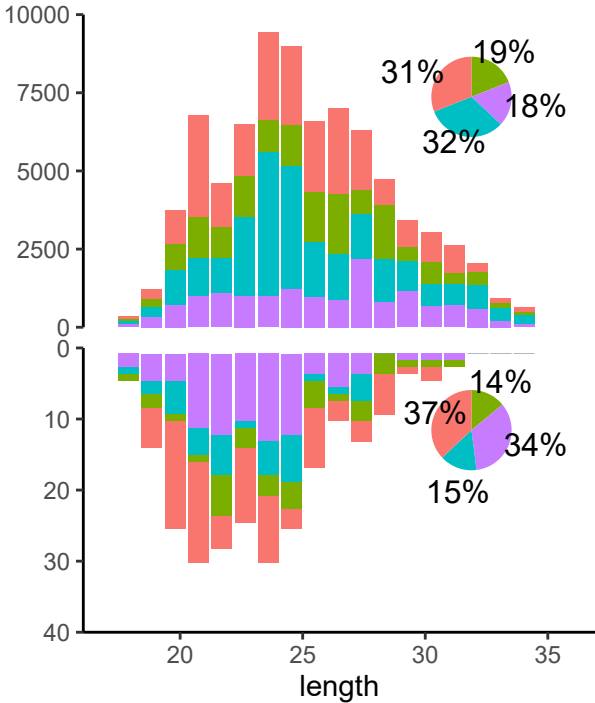

Supplement: Supplementary Figure 1 — Description of sRNA libraries in control and SARS-CoV-2-infected Vero cells. (A) Correlation of miRNA reads between two biological replicates in control and SARS-CoV-2-infected (6 hpi/12 hpi) Vero cells. (B) PCA plot of miRNA-seq libraries of control and virus-infected (6 hpi/12 hpi) Vero cells. [file DataSheet_1.zip › Data Sheet 1/SARS-CoV-2 source code & data/SARS-CoV-2 source code/figs4/output/Figure S4C-Calu-3-12h-SARS-CoV-2.pdf]

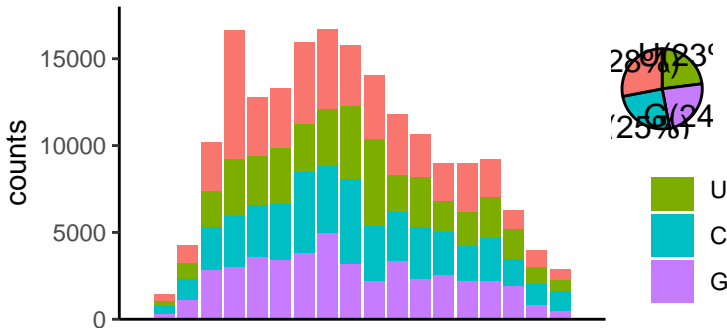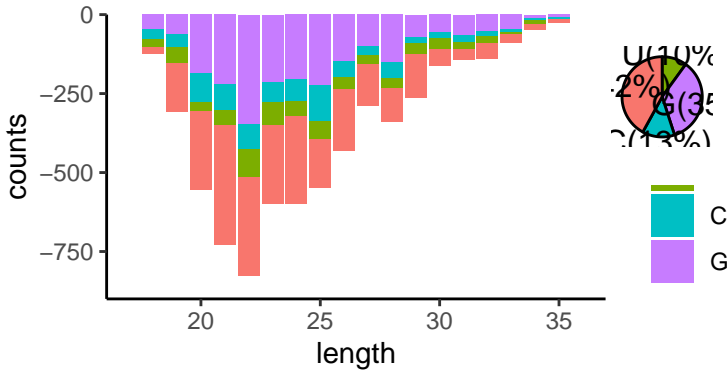

Supplement: Supplementary Figure 1 — Description of sRNA libraries in control and SARS-CoV-2-infected Vero cells. (A) Correlation of miRNA reads between two biological replicates in control and SARS-CoV-2-infected (6 hpi/12 hpi) Vero cells. (B) PCA plot of miRNA-seq libraries of control and virus-infected (6 hpi/12 hpi) Vero cells. [file DataSheet_1.zip › Data Sheet 1/SARS-CoV-2 source code & data/SARS-CoV-2 source code/figs4/output/Figure S4C-Calu-3-24h-SARS-CoV-2.pdf]

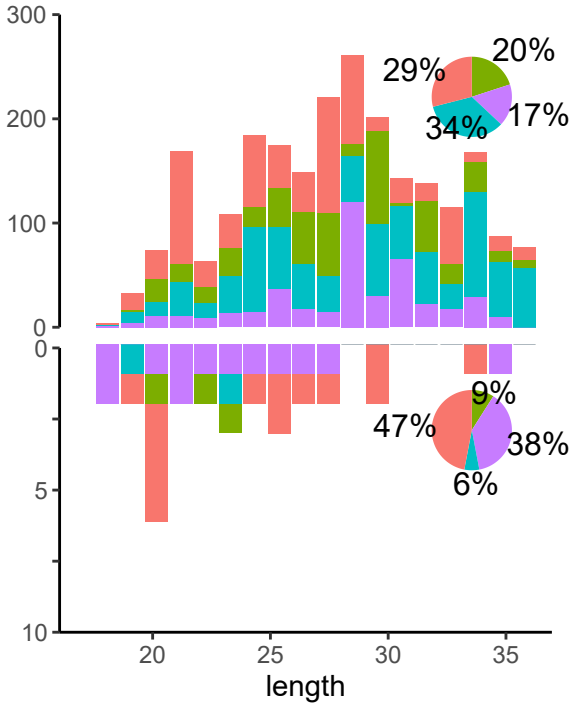

Supplement: Supplementary Figure 1 — Description of sRNA libraries in control and SARS-CoV-2-infected Vero cells. (A) Correlation of miRNA reads between two biological replicates in control and SARS-CoV-2-infected (6 hpi/12 hpi) Vero cells. (B) PCA plot of miRNA-seq libraries of control and virus-infected (6 hpi/12 hpi) Vero cells. [file DataSheet_1.zip › Data Sheet 1/SARS-CoV-2 source code & data/SARS-CoV-2 source code/figs4/output/Figure S4C-Calu-3-4h-SARS-CoV-2.pdf]

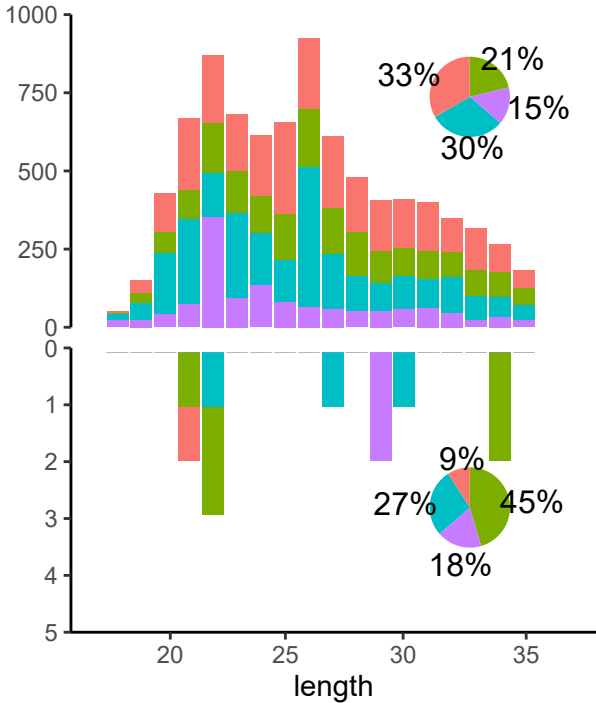

Supplement: Supplementary Figure 1 — Description of sRNA libraries in control and SARS-CoV-2-infected Vero cells. (A) Correlation of miRNA reads between two biological replicates in control and SARS-CoV-2-infected (6 hpi/12 hpi) Vero cells. (B) PCA plot of miRNA-seq libraries of control and virus-infected (6 hpi/12 hpi) Vero cells. [file DataSheet_1.zip › Data Sheet 1/SARS-CoV-2 source code & data/SARS-CoV-2 source code/figs4/output/Figure S4D-Calu-3_MOI10-SARS-CoV-2-24h.pdf]

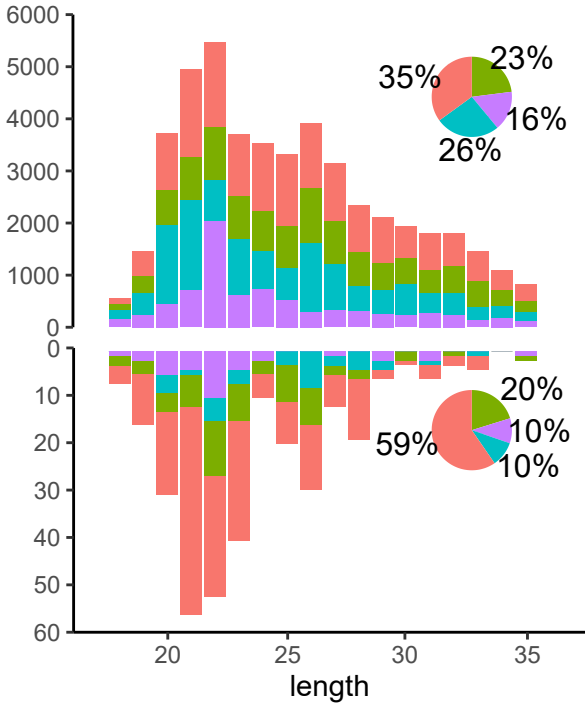

Supplement: Supplementary Figure 1 — Description of sRNA libraries in control and SARS-CoV-2-infected Vero cells. (A) Correlation of miRNA reads between two biological replicates in control and SARS-CoV-2-infected (6 hpi/12 hpi) Vero cells. (B) PCA plot of miRNA-seq libraries of control and virus-infected (6 hpi/12 hpi) Vero cells. [file DataSheet_1.zip › Data Sheet 1/SARS-CoV-2 source code & data/SARS-CoV-2 source code/figs4/output/Figure S4D-Calu-3_MOI10-SARS-CoV-2-36h.pdf]

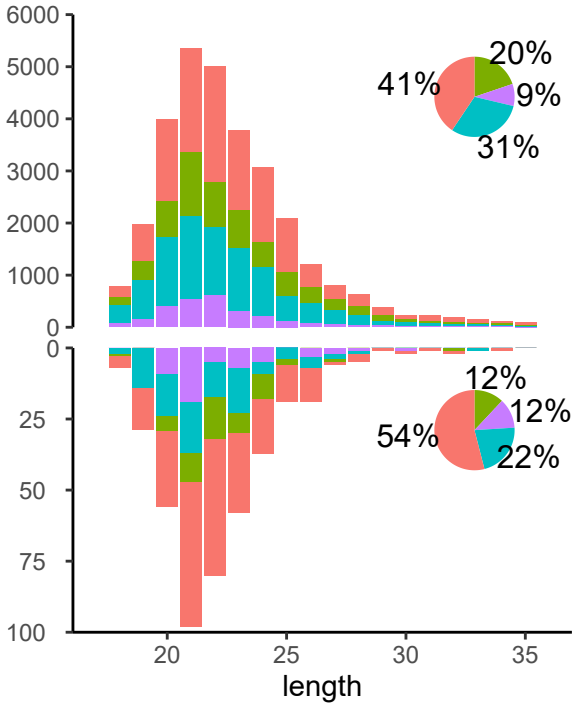

Supplement: Supplementary Figure 1 — Description of sRNA libraries in control and SARS-CoV-2-infected Vero cells. (A) Correlation of miRNA reads between two biological replicates in control and SARS-CoV-2-infected (6 hpi/12 hpi) Vero cells. (B) PCA plot of miRNA-seq libraries of control and virus-infected (6 hpi/12 hpi) Vero cells. [file DataSheet_1.zip › Data Sheet 1/SARS-CoV-2 source code & data/SARS-CoV-2 source code/figs4/output/Figure S4D-Calu-3_MOI10-SARS-CoV-2-48h.pdf]
