# Supplementary material for: SARS-CoV-2 RNAs are processed into 22-nt vsRNAs in Vero cells
Source: Front Immunol. 2022 Oct 28;13:1008084. doi: 10.3389/fimmu.2022.1008084 (PMC9650353; doi:10.3389/fimmu.2022.1008084)
Supplement: Supplementary Figure 1 — Description of sRNA libraries in control and SARS-CoV-2-infected Vero cells. (A) Correlation of miRNA reads between two biological replicates in control and SARS-CoV-2-infected (6 hpi/12 hpi) Vero cells. (B) PCA plot of miRNA-seq libraries of control and virus-infected (6 hpi/12 hpi) Vero cells. [file Vero_control-12h-1.pdf › Data Sheet 1/SARS-CoV-2 source code & data/SARS-CoV-2 source code/fig4/output/Vero_control-12h-1.pdf]

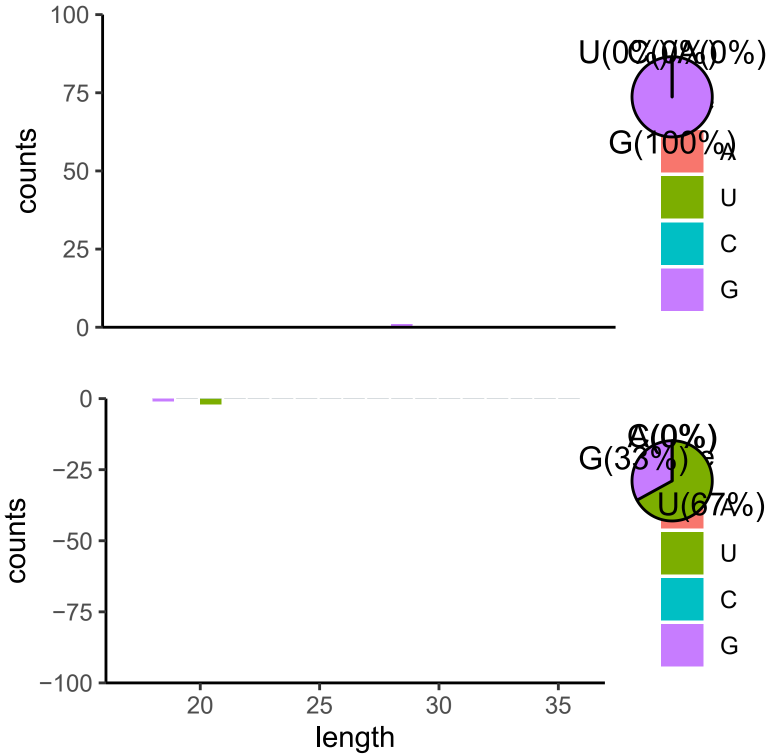

100
75
50
25
0
counts
U(0%)
C(0%)
A(0%)
G(100%)
A
U
C
G
0
−25
−50
−75
−100
counts
C(0%)
A(0%)
G(33%)
U(67%)
A
U
C
G
20
25
30
35
length
